# What constitutes a community? A co-occurrence exploration of the Costa Rican avifauna

**DOI:** 10.1080/23766808.2023.2204549

**Published:** 2023-04-27

**Authors:** Mélusine F. Velde, Elizabeth M. Besozzi, Billi A. Krochuk, Kate M. Henderson, Brian R. Tsuru, Sara Velásquez Restrepo, Holly M. Garrod, Jacob C. Cooper

**Affiliations:** a Division of Birds, Negaunee Integrative Research Center, Chicago, IL, USA; b Faculty of Natural Sciences, Imperial College London Silwood Park, Ascot, UK; c Biological Sciences Division, The College at University of Chicago, Chicago, IL, USA; d Department of Biology, University of Oklahoma, Norman, OK, USA; e Biodiversity Research Centre and Department of Zoology, University of British Columbia, Vancouver, BC, Canada; f Department of Environmental Biology, State University of New York College of Environmental Science and Forestry, Syracuse, NY, USA; g School of Environment and Natural Resources, Ohio State University, Columbus, OH, USA; h Departamento de Ciencias Biológicas, Universidad EAFIT, Medellín, Colombia; i BirdsCaribbean, Natick, MA, USA; j Department of Biology, Villanova University, Villanova, PA, USA; k Biodiversity Institute & Natural History Museum, University of Kansas, Lawrence, KS, USA; l Committee on Evolutionary Biology, University of Chicago, Chicago, IL, USA

**Keywords:** Ecological niche modeling, community assembly, neutral theory, community turnover, co-occurrence

## Abstract

The concept of a “community” as a form of organization for natural biological systems is both widespread and widely accepted within the ecological and biological sciences. Communities have been defined as groups of organisms that interact in ways that denote interdependence between individuals and taxa (e.g. as defined by “food webs”) but they have also been defined as groups of co-occurring organisms that are assumed to interact by virtue of their shared spatiotemporal existence. The latter definition has been debated and challenged in the literature, with mounting evidence for co-occurrence being more indicative of coincident ecological niches in space and time rather than being evidence of ecological interaction or dependency. Using a dataset of 460 Costa Rican bird species divided into breeding and non-breeding season datasets, we empirically demonstrate the ways in which co-occurrence can create illusory communities based on similar occupied ecological niches and similar patterns of co-occurrence at different times of year. We discuss the importance of discerning coincidental co-occurrence from true ecological interactions that would manifest a true community, and further address the importance of differentiating communities of co-occurrence from communities of demonstrable ecological interaction. While co-occurrence is a necessary aspect of interspecific interactions, we discuss and demonstrate here that such co-occurrence does not make a community, nor should explicit patterns of co-occurrence be seen as evidence for evolutionarily important ecological interactions.

## 1. Introduction

Community ecology is a relatively recent branch of ecological inquiry that has been both shaped and plagued by semantic inconsistencies [1,2] related to the complexity of its underlying processes (reviewed in McIntosh [3]). The susceptibility of the focal unit of community ecology, the ecological community, to inconsistent usage is an apt example of how imprecise definitions can influence the trajectory of a field. Ecological communities are typically characterized as groups of organisms that coexist in time and space. While this definition is succinct, it is also contentious, as the exact qualifications for coexistence are debatable and subject to variation across spatial scales [1].

Communities are typically defined as independent co-occurrences among multiple species in a given geographic space [4–6], but are also often defined as integrated, interacting groupings of species [7]. In a research context, the former is best represented by communities or ecological networks inferred from co-occurrence data [8,9], and the latter by specific studies on interactions between individual organisms.

The degree of intersection between spatiotemporal definitions of community and species co-occurrence hinges on ecological interactions, with proponents suggesting that co-occurrence can be used to predict ecological interactions, or at least act as a proxy for ecological interactions [10,11]. Critics, however, argue that co-occurrence is a purely random phenomenon; in other words the *possibility* of interaction fostered by co-occurrence is not necessarily related to the *probability* of interaction among co-occurring species [9,12]. Despite the lack of consensus, co-occurrence is still considered an important metric, especially at local scales for within-clade studies, and at large phylogenetic or spatial scales for determining community assembly [6,8,13–15].

Well-studied examples of species’ co-occurrence exist with evidence for extreme levels of interaction between co-occurring taxa, whether via commensal or amensal interactions. These co-occurring taxa exist in tightly-knit, spatiotemporally-consistent communities, with coterminous or nearly coterminous home ranges at the local level, as is found in Neotropical mixed-species bird flocks [16,17]. However, even these tightly-knit flocks, which can operate as “meta-organisms” within their environments, experience faunal turnover both spatially (e.g. different faunal components in spanning geographic regions [16,18]) and temporally (e.g. individual replacement, microhabitat selection, and seasonal contributions from migrants [19]). Interactions between individual species and analysis of the effects of life histories on these interactions can be examined on a local scale within these flocks; however, community assembly and turnover is not necessarily influenced by these dynamics. Therefore, while co-occurrence and local (i.e. within community) interactions do exist, what constitutes a true, manifested “community” appears debatable and highly dependent on the spatiotemporal context of the group of organisms in question. This emphasis on spatial co-occurrence as a metric for determining community composition is one reason why the “disintegration” of classic communities has been proposed, as co-occurrence does not imply closely aligned community dynamics [1].

To understand how patterns of co-occurrence may inform community dynamics and shifts through space and time, we performed multiple clustering analyses for the Costa Rican bird community, which is both well-sampled and well-studied. Specifically, we used these clusters to analyze patterns of community cohesion, or how little communities change through time, and we quantified niche stability throughout the annual cycle by assessing these co-occurrence patterns derived from ecological niche models. We created two parallel pipelines for these data, one taking biogeographic restrictions on distribution into account, and another model looking at “neutral” dynamics of ecological niche diversification without presumed geographic barriers to determine community turnover [13]. These methods allow for a rigorous evaluation of co-occurrence for a large, well-documented avifauna throughout the year to demonstrate whether true community cohesion exists at coarse scales.

## 2. Methods

### 2.1. Terminology

To clarify discordant terminology, we unambiguously define a community as a set of spatiotemporally co-occurring species that exhibit cohesion with regard to species composition throughout the year. This definition, which deviates from traditional usage, captures the assumption that direct or indirect interaction is a fundamental feature of communities, an assumption that is both implied [1] and explicit [2] in the literature. We refer to those sets of organisms that may exhibit spatiotemporal co-occurrence but not necessarily cohesion as ensembles, a term rarely used in the literature [2] and that is therefore unlikely to confer field-specific biases. Stroud *et. al* [2] treat the original definition of ensemble proposed in Fauth *et. al* [20] as redundant with that of community and assemblage. However, we believe the advantages of co-opting a term already in the community ecology lexicon (and a term that is so rarely used as to have only negligible existing connotations) outweigh the detriments of potential redundancy. We avoid the term assemblage due to its phylogenetic connotations.

### 2.2. Analytical tools

We used *R* versions 1.3.959 and 4.0.4 [21] as well as QGIS versions 3.10.6 and 3.18 [22]. We used the *R* packages *data.table* [23], *gridextra* [24], *tidyverse* [25], and *viridis* [26] for general data manipulation. General image manipulation was performed with *Inkscape* [27] and *ImageMagick* [28]. We used species distribution models (SDMs) from a previous manuscript in this project [29]; we also provide an overview of the data processing pipeline here.

### 2.3. Study area

We chose Costa Rica as our study area because of its relatively small size (*c*. 51100 km^2^), its topographically diverse landscape (with Caribbean and Pacific lowlands and central cordilleras reaching 3819 masl), and its well-documented avifauna [29], which has generated a large volume of distributional data accessible via the community science data repository eBird [30,31]. Furthermore, the phenomenon of elevational migration among resident Costa Rican species is well-documented in the literature, providing ample context for analyzing community cohesion throughout the annual cycle [32].

### 2.4. Data cleaning

We used a previous dataset of all eBird observations from Costa Rica and Panama (downloaded from version ebd_relAug-2019) that was used to model species in those areas [29]. Data from eBird are classified using the eBird/Clements checklist to the birds of the world, with the data download corresponding to a 2019 edition of the list [33] and information in this manuscript reflecting a more recent taxonomic update [34]. This dataset removed checklists that were >10 km in distance and >500 minutes in duration to avoid spatiotemporal biases in effort. eBird checklists were filtered by month, creating a dataset of June observations and a dataset of December observations. This allowed us to compare patterns of species’ co-occurrence and account for latitudinal migrant turnover in the Costa Rican avifauna during the Northern Hemisphere summer and winter, which correspond to the Neotropical rainy season (June), and dry season (December), respectively. We spatially thinned the data to minimize pseudoreplication by removing points within 1 km (i.e. the distance of a single grid cell) of other points for each species, and then removing species with fewer than 10 observations, the minimum number of points required for our modeling pipeline [35].

### 2.5. Species distribution models

Using the same dataset, we created two parallel sets of SDMs for both a northern temperate summer dataset (June) and a northern temperate winter dataset (December). These datasets utilized all available eBird points with the exception of two artificial absences of points within 10 km of Montes del Oca, NE of San José (San José Province) and the Costa Rica Bird Observatories’ Madre Selva Station (San José and Cartago Provinces); these absences were holdovers from previous research with this dataset [29]. June and December were selected as these are the months of the opposite solstices and the months during which communities are relatively “stable”, with little overturn in species composition from latitudinal and elevational migration. Occurrence points for each species were correlated with 9 environmental datalayers with a grid cell size (i.e. resolution) of 1 km^2^ (Table 1, for methods, see [29,36]). Ecological niche models were created using minimum volume ellipsoids (MVEs) trained on the entire cloud of data points [36,37]. These ellipsoids are sometimes characterized as having the center of the ellipsoid represent the most suitable habitats available given the data presented [36–39], although further research has shown the center of the ellipsoid does not necessarily reflect that species’ niche centroid [39,40]. To remove environmental outliers that could represent genuine vagrants or misidentifications, minimum-volume ellipsoids used a 75% data inclusion threshold for conversion to binary species’ distribution models, as this threshold was found to be most accurate within the region for predicting species occurrence *en masse* compared to other thresholds [29]. In this study, we restricted these ecological niche models (ENMs) to the boundaries of Costa Rica, with the resulting SDMs predicting the areas in which species could be found within the same boundaries. Thus, models were created using the whole of the Costa Rica-Panama region (a unique, biogeographic area where many taxa are endemic or represented by endemic subspecies), and subsequently restricted to our primary study region of Costa Rica. We focused this study on Costa Rica specifically as this is where a majority of the data used to train the models were collected, and because this is an area where the local avifauna is more well-known.

### 2.6. Presence-absence matrix creation

In general, ENMs fail to exclude suitable but inaccessible habitat where specific taxa do not occur. To account for “neutral” dynamics and those informed by biogeographic barriers, we generated two sets of models: one unconstrained, including the entire country of Costa Rica, and one constrained to biogeographic regions within Costa Rica (**M**s, *sensu* Soberón and Peterson [41]) [42,43]. Because our SDMs were constructed using presence-only data and therefore did not require pseudo-absences or confirmed absences for training, we fitted biogeographic regions *post hoc*. We used the *R* packages *raster* [44], *rgdal* [45], *rgeos* [46], and *sf* [47] to create a secondary set of distribution rasters clipped to two biogeographic regions: the Pacific and Caribbean slopes of Costa Rica, divided approximately along the crest of the central highlands. We completed the remaining steps both for SDMs limited to individual biogeographic regions, and for “neutral” models that lacked any biogeographic restrictions.

For both June and December, we overlaid occurrence points onto the two biogeographic regions and clipped the output distribution rasters to these regions using the aforementioned *R* packages and *dismo* [48], creating biogeographically restricted range estimations. We then fit a hexagonal sampling array over the study area with 372 hexagonal cells with distances between centroids of *c*. 13.6 km (based on centroid coordinates) to create a presence-absence matrix (PAM) with a reduced resolution to facilitate downstream clustering analyses and to account for minor spatial uncertainties between birds that occur in the same geographic area. This hexagonal grid possessed cells of *c*. 164.8 km^2^. We opted for a hexagonal grid because they efficiently cover spatial areas and have been used in other studies utilizing eBird data [49]. We extracted hexagonal array species coverage using *velox* [50], and constructed sliding windows of distribution grid cell coverage for inclusion in the derived presence-absence matrix based on the number of SDM grid cells within each hexagonal PAM array cell. Hexagons were considered to include any SDM grid cells for which the centroids fell within the hexagon. As these geometries and sizes do not match up exactly, even when scaled, the number of SDM grid cells per hexagon varied greatly. To convert from the high-resolution SDM grid cells to the lower-resolution hexagonal grid, we counted a hexagon as “present” for the species if 70% of SDM grid cells were “present” when a hexagon overlapped with 10 or fewer SDM grid cells, but we changed this ratio to 50% presence when the number of overlapping SDM grid cells was between 11 and 40, and 30% presence if more than 40 SDM grid cells overlapped with a hexagonal array cell (maximum number of SDM cells per array is *c*. 645).

### 2.7. Ecostructure

To understand broad spatial patterns of occurrence, we passed biogeographically restricted data and “neutral” data through the program *ecostructure* [51]. This program is similar to the algorithm *structure*, which is designed for the analysis of genetic data; ecostructure applies algorithms usually used for single-nucleotide polymorphisms (SNPs) to presence-absence matrices of species occurrence across geographic space [51–53]. Thus, the output of ecostructure is a representative of the relative “motif” contribution to each individual geographic cell, where each motif is a community of co-occurring taxa. We used a custom loop code to apply the same conditions to each biogeographic or “neutral” scenario, running each model with tolerance of 0.1 for 10 iterations, with the number of clusters *K* varying between 2 and 14. We used basemap data from *rnaturalearth* [54] to plot these data within the spatial extent of Costa Rica.

### 2.8. Co-occurrence analyses & niche similarity

We adapted code from Cooper [55] to examine clustering within the dataset, using aforementioned packages as well as *ape* [56,57] to visualize and work with clusters and cluster dendrograms. We determined the optimal *K*-value for *K*-means cluster numbers using gap-statistic analysis with the *factoextra* command “fviz_nbclust” [58]. We passed biogeographically trained and “neutral” data separately through a pipeline that determined cluster assignments for each species based on this optimal *K*-value, using the *R* function *kmeans* [21]. We visualized cluster assignments using the hierarchical, bottom-up unweighted pair group method with arithmetic mean (UPGMA) method in *R* using *hclust* [21,59], but did not use this clustering method for downstream analyses.

To explore community stability between June and December, species lists with *kmeans* cluster assignments needed to contain only year-round residents of Costa Rica. We excluded migrants at this stage because migrants are only present at one time of year, and perturbations or cohesive movements by resident species in response to migrants should be visible even without their direct inclusion. Seasonally biased occurrence records (e.g. too few records during a certain season) were also cause for exclusion, and are likely attributable to unequal year-round observer effort in the region (Table S1 [29]). We cross-compared clusters between June and December to determine how each species’ cluster assignment changed across seasons. Given that the ideal number of clusters differed between seasons, we categorized clusters as stable (>66% of taxa co-occurring within a cluster in both seasons), split (33–66% of taxa co-occurring between different seasons), or diffuse (no more than 33% of taxa co-occurring between seasons). These cross-comparisons excluded migratory taxa, and only included species present all year round within Costa Rica. We also visually inspected the stacked SDMs (i.e. richness maps for each cluster) for each season to observe spatial patterns.

We calculated niche similarity metrics for all species between June and December. We used the *R* package *dismo* [48,60] to compute Schoener’s *D*, a metric comparing similarity in geographic distribution, from the continuous outputs of the ENMs to determine the similarity between summer and winter niches for each species. Using this metric, we examined the distribution of seasonal niche differentiation for each species, and compared species distributions across clusters. We randomized Schoener’s *D* by comparing random species’ models from summer and winter to determine whether the observed distribution of *D* statistics differed significantly from a random expectation. *D* statistics were also compared to the difference in the number of points between June and December to explore the relationship between observed niche differentiation and seasonal bias in observation records.

To account for the role of migrant taxa in shaping regional patterns of community assembly and niche stability, we subsetted our data into: 1) taxonomic clades that contain migrant taxa during some portion of the year, and 2) 500 randomly subsampled groups equal in size to the migrant species group (*n* = 162, Table S2). The migrant and random subsamples were compared using the same niche equivalency (i.e. niche similarity) techniques described above, and were binned into the same categories as mentioned above (stable, split, or diffuse) or into an unknown category for groups with <5 taxa. We performed Fisher’s Exact Tests using the *R* function *fisher.test* [21] to determine whether the behavior of clusters with migrants differed from random subsets of the data, or from the distribution of the entire dataset.

## 3. Results

**Figure 1.**
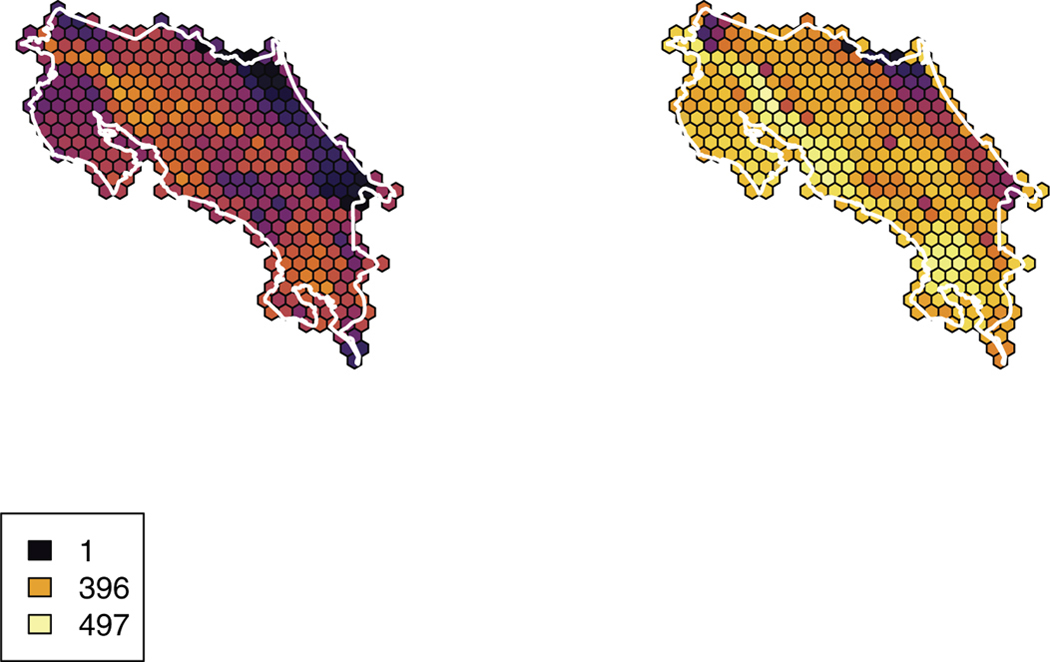
Species richness (i.e. gross number of species predicted to occur) during June (left) and December (right) in Costa Rica. Legend displays values for no taxa, the summer maximum, and the winter maximum. Includes all taxa, including those removed from cluster analyses.

**Figure 2.**
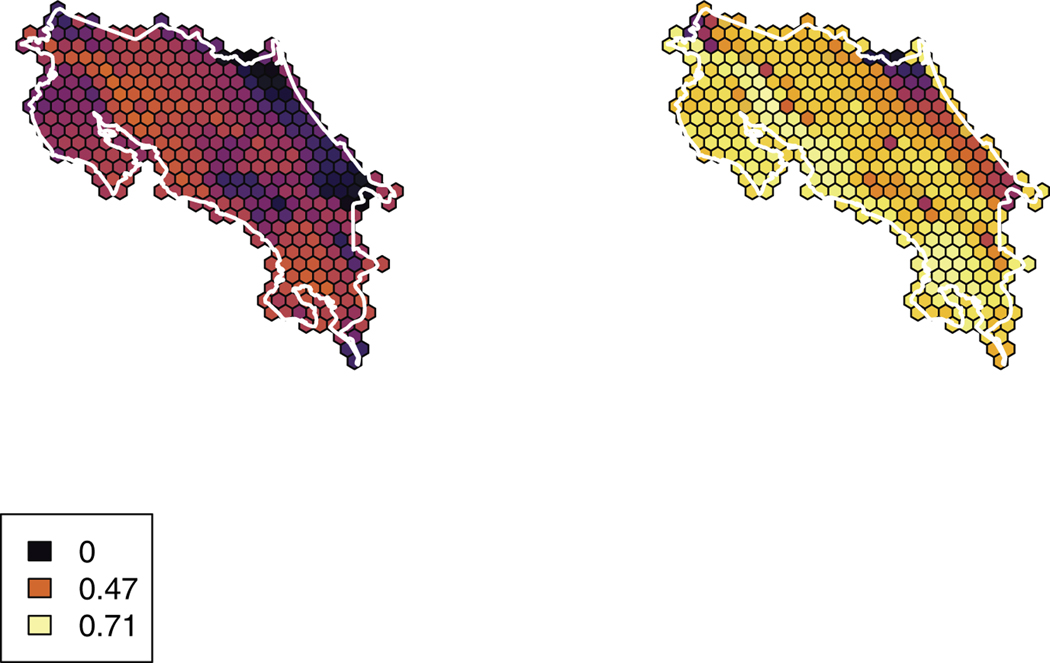
Beta diversity (i.e. average range size per cell) for birds in Costa Rica in June (left) and December (right). Legend displays values for no taxa, the summer maximum, and the winter maximum. Includes all taxa, including those removed from cluster analyses.

We obtained a list of 719 regularly occurring species in Costa Rica (Table S1). We manually omitted some migratory taxa (specifically, those that may not be fully removed via other cleaning steps) and taxa that are not considered “landbirds” (e.g. Magnificent Frigatebird (*Fregata magnificens*); *n* = 142). We then omitted taxa that had insufficient sampling during one time of year (either due to migratory habit or lack of observations; *n* = 117) including nine resident and one migrant taxa due to databasing errors related to annotating taxa for exclusion. We therefore resulted in a list of 460 species in our overall co-occurrence dataset with 162 species in our migratory subset (i.e. latitudinal migrants and their close relatives). Species richness (*α* diversity) and species’ average range size (*β* diversity) were higher in the December dataset than the June dataset, and these values were also higher for the Pacific Slope than for the Atlantic Slope (Figures 1,2).

### 3.1. Biogeographically-constrained data

Our gap-analysis established 13 clusters for the December dataset and 16 for the June dataset. We found that 4 clusters were stable between time periods, 2 split into different groups, and 10 clusters were diffuse. Stable clusters were found in the Atlantic Lowlands, the southern Pacific Lowlands, the northern Pacific Lowlands, and the high elevations of the Talamanca Cordillera. Split and diffuse groups were much more geographically widespread, and often expanded from more conserved areas in June to more widespread areas in December or vice versa (e.g. one cluster found in the foothills in summer was widespread in the foothills and lowlands during the winter). Comparing the distribution of niche similarity between cluster categories via Wilcoxon rank sum tests did not yield any significant results (smallest *p* = 0.13, stable vs. diffuse), suggesting that there is no significant difference in seasonal ecological similarity between groups of differing cohesiveness through the annual cycle. For biogeographically-constrained models, the distribution of *D* statistics were significantly higher, thus denoting more niche similarity, than randomly generated *D* values.

### 3.2. Neutral models

Gap-analyses on neutral datasets (i.e. no training areas constraining SDMs) recovered 11 distinct clusters for December and 18 distinct clusters for June. For neutral models, we found 3 stable clusters (though 2 would be considered split if viewed from winter into summer instead of summer into winter), 3 split clusters, and 12 diffuse clusters. Two stable clusters corresponded to groups of birds found in the northwestern Pacific lowlands and the third was localized in the higher elevations of Costa Rica’s cordilleras, being more geographically localized in June than in December. Two split clusters were widespread across the country showing little pattern, but the third illustrated a shift from highlands to highlands plus adjacent lowlands in December. Notably, comparisons of *D* statistic distributions (i.e. niche similarity) were more significant when examining cluster categories of neutral communities than for shifts within the constrained models. All comparisons had *p* ≤ 0.05 (highest *p* = 0.05 for stable vs. split clusters), in the cases of stable vs. diffuse (Wilcoxon rank sum test, *W* = 653, *p* = 0.04), split vs. diffuse (*W* = 582, *p* << 0.05), and stable vs. split (*W* = 354, *p* = 0.05). The mean similarity of communities across the annual cycle (shown here with ±95% CI) was lowest (i.e. niches were more different between seasons) for diffuse communities (*D* = 0.865 ± 0.014) followed by stable communities (*D* = 0.888 ± 0.017) and lastly split communities (*D* = 0.908 ± 0.017). For random iterations of neutral models, the distribution of D statistics were significantly higher, thus denoting more similarity, than randomly generated *D* values.

### 3.3. Geographic structuring

**Figure 3.**
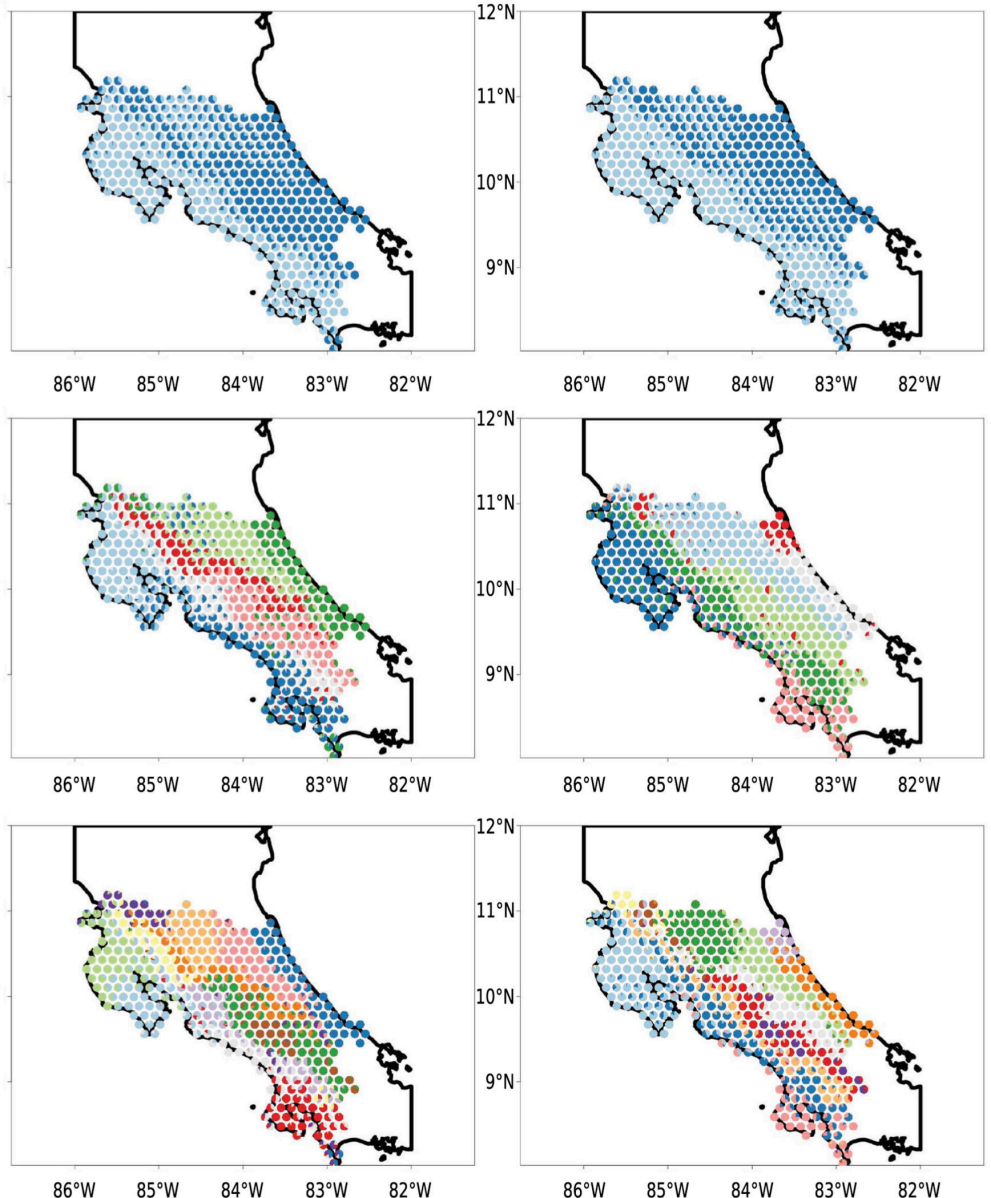
Plots from *ecostructure* showing results for June (left) and December (right) with K values of 2 (top), 7 (middle), and 13 (bottom). Note that the plots become structured in a similar fashion and display patterns reminiscent of the richness and beta diversity plots, indicating common patterns of distribution structuring the geographic motifs.

Analyses with the *R* package *ecostructure* found motifs corresponding to clades identified in the aforementioned clustering analyses. As we increased the number of groups (*K*), *ecostructure* first identified community splits corresponding to the Pacific and Atlantic slopes of Costa Rica, with the second major split (i.e. *K* = 3) involving the central highlands. With increasing *K*, more precise ecological regions within Costa Rica were identified as distinct groups. We limited *ecostructure* analyses to *K* = 14, in part because of the processing time required to run the models, and in part because fine-scale *ecostructure* models do not partition space in the same way as hierarchical clustering does (in *ecostructure*, each individual site can be assigned to multiple biogeographic motifs). For the higher values of *K*, we recovered many recognized biogeographic areas within the landscape, for example the marshlands of northern Costa Rica where the Nicaraguan Grackle (*Quiscalus nicaraguensis*) occurs, and elevational stratification between highland and foothill bird communities, both in eastern and western Costa Rica (Figure 3). Group demarcation differed between summer and winter, with different distributions for clusters in all geographic regions varying by season (though lower-order classifications, such as *K* = 2, were more similar than higher order classifications). Biogeographic breaks were apparent but not consistent when comparing outputs between seasons and for different levels of *K*, reflecting variable amounts of overlap amongst species and between seasons.

### 3.4. Niche comparisons & migrant subsetting

Niche similarity across seasons showed similar patterns for both neutral and biogeographically trained models. The least conserved niches year-round belonged to taxa in the lowlands (e.g. the Chestnut-colored Woodpecker (*Celeus castaneus*), *D* = 0.645) and some foothill and montane taxa (e.g. the Azure-hooded Jay (*Cyanolyca cucullata*), *D* = 0.706), while the most similar niches year-round belonged to widespread taxa (e.g. the Rufous-collared Sparrow (*Zonotrichia capensis*), *D* = 0.937) and taxa that specialize in particular habitats (e.g. the Ruddy-capped Nightingale-Thrush (*Catharus frantzii*), *D* = 0.938). Subsetting biogeographically-constrained data into groups of residents and migrants did not change the overall results, with non-randomized *D* statistics being more similar than randomized statistics for niche similarity (*t* = −32.06, *df* = 627.11, *p* <<< 0.05). Comparisons of both stable and split communities were not significantly different from each other, but both approached the threshold for significance when compared with diffuse communities (*p* = 0.07 and *p* = 0.06, respectively). We found that the assignment of migrants to different groups was no different than assignments given to random subsets of the data (Fisher’s Exact Test, *p* = 0.53).

## 4. Discussion

Species distributions of the Costa Rican avifauna illustrate how communities can randomly emerge from spatial coincidence given similar ecological or physiological requirements. In Costa Rica, this manifests in areas like the Talamanca Highlands, where the taxa with northern and southern biogeographic origins overlap locally, and whose populations and close relatives overlap in few, if any, other geographic localities in the region. Even when birds apparently show close ecological associations (e.g. recognizing and responding to each other in mixed-species flocks), the occurrence of one species does not necessarily predicate the occurrence of any other member of that association [16,17,19,61]. This observation aligns with studies of species distributions in past glacial cycles, where species could co-occur and be part of the same community in historical climates, but occur in geographically disparate regions today [62]. Indeed, many dynamics of community co-occurrence could be equally attributable to coincidental convergence of ecological and physiological needs over direct relationships or any form of community cohesion or interaction [9].

Explanations for patterns of co-occurrence are inextricably tied to the multitude of niche concepts intended to explain species distributions (reviewed in Sales *et al*. [63]). The diversity of overlapping niche concepts fall along axes of biotic to abiotic predictors (e.g. Elton [64] vs Grinnell [65]), individual- to species- or population-level possession of the niche itself (e.g. Chase and Leibold [66] vs. Soberón and Peterson [41]), and the extent to which the environment either shapes or is shaped by the niche (e.g. MacArthur and Levins [67] vs. Hutchinson [68]). Concepts such as these can be juxtaposed with neutral models of species assembly, which posit that existing species distributions and patterns of co-occurrence are the product of random processes through time, such as ecological drift, speciation, and dispersal capacity [69,70]. However, these influences appear to vary with regards to changing spatial scales, suggesting that we may be falsely dichotomizing an underlying scale-dependent continuum of niche-to-neutral drivers of species distribution [71]. Within the Costa Rican avifauna, it is clear that some species are linked to specific Grinnellian (i.e. “habitat”) niches, such as the páramo, but that the overall regional community lacks cohesion and does not maintain co-occurrence (i.e. community cohesion) throughout the year.

In many instances where we see direct interaction between at least two species within a community, as is the case in Interspecific Social Dominance Mimicry (ISDM), the beneficial community trait (in this case mimicry) and the species that possesses it are not necessarily restricted to regions of geographic co-occurrence. In North America, the Pileated Woodpecker (*Dryocopus pileatus*), widely regarded as a mimic of the now extinct Ivory-billed Woodpecker (*Campephilus principalis*), has a distribution that extends far beyond where *Campephilus* ever occurred, suggesting that the factors driving this association were not necessary for the survival of *D. pileatus* in communities where it occurred in the absence of *Campephilus* [72]. Likewise, other widespread mimics, like Hairy and Downy Woodpeckers (*Dryobates [Leuconotopicus] villosus* and *Dryobates pubescens*, respectively), show differential responses to different ecological factors, confirming that ecological requirements for taxa are unlinked from real community interactions [73].

For the most part, we find stable community composition in regionally unique habitats that differ from the surrounding geographic matrix, such as the montane highlands or the northern Caribbean lowlands (Figure 3). These regions host many species restricted to their respective habitats and environments, such that a cohesive “community” can be inferred from shared patterns of co-occurrence that are more than likely coincidental. For example, we recover Red-tailed Hawk (*Buteo jamaicensis*) and Buffy-crowned Wood-Partridge (*Dendrortyx leucophrys*) in the same stable community within the biogeographically-constrained data. Our analyses show these taxa are largely found in similar areas of Costa Rica; however, in a continental context, it is clear these taxa are not part of a community, as *B. jamaicensis* is a widespread North American taxon that becomes increasingly montane as one moves south, and *D. leucophrys* is exclusively montane in southern North America [74]. Microhabitat selection could further serve to separate taxa such as these, especially in areas where foraging habitats and cover selection differ. Therefore, although co-occurrence enables potential community interaction, it is probable that the community association recovered here is the result of similar realized niches at the local level and not the result of a holistic, regional community dynamic [75]. It is therefore plausible that many widespread taxa, such as *B. jamaicensis*, never truly belong to any given community of species, but rather that they are able to exploit ecological niches that overlap with a variety of different species’ pools and reside within a species’ geographically accessible niche space [41].

The existence of stable and diffuse communities within our Costa Rican dataset, and the fact that the proportions of these communities in a biologically relevant subset of our data are indiscernible from random subsets of the data, illustrate the danger in treating co-occurring organisms as a community. While ecological interactions do exist and are extremely important for species at the local (e.g. individual or population) level, these interactions are somewhat mutable, such that even the classic mixed-species flocks of the Neotropical lowlands are perhaps better considered a character of specific taxonomic groups rather than stable and defined communities that exist across a broad swath of territory. The role of ecology in bolstering the illusion of community cohesion is reinforced by the significance of ecological niche differences within the neutral dataset, but not within the biogeographically-constrained dataset. Neutral models, which allowed species to occupy all suitable habitats, recovered significant differences between communities, likely because species with similar ecologies were allowed to co-occur more broadly across the study region. Constrained models, which separated ecologically similar, but geographically isolated taxa (e.g. the Blue-tailed Emerald (*Chlorostilbon mellisugus*) and the Garden Emerald (*C. assimilis*)), lacked the differences in niche similarity between different classifications of community. Neutral theory, wherein species can colonize and move freely about a matrix, may therefore be more informative in certain contexts for differentiating communities assembled only through overlapping ecological niches at coarse scales [69].

Regardless of the use of biogeographic training areas, we do find strong evidence for an “ecoregion” view of Costa Rica. Our partitions of Costa Rican communities between biogeographic community motifs reflects the patterns shown in existing ecoregion assessments [76]. These partitions, often based on unique habitats and the specific flora and fauna that comprise them, are a perfect example of biogeographic restrictions compounding co-occurrence among taxa that are already potentially co-occurring only as a result of “neutral” processes. Within these communities, additional compounding factors exist, such as metabolism, disease, and temperature, that may be restricting species from truly exploring or colonizing their entire accessible ecological matrix [77]. Such “communities” are perhaps best seen as conglomerations of species honing in on similar ecological niches and therefore evolving in tandem, rather than taxa that are reliant on each other as part of a holistic community.

While this study did not incorporate phylogenetics to examine how community composition changes across space in time, a parallel study could be done to understand how phylogenetic niche conservatism fits into community stability and turnover. According to patterns of niche conservatism, closely-related species share niche-related traits and therefore are more likely to occupy the same niche. If communities experience high turnover in space and time, they can more easily maintain their patterns of niche conservatism by reducing the consistent competition that closely-related species face when confronted with similar niches [78]. Phylogenetic analyses of the communities that have been examined in this paper could further contribute to our understanding of the effects of phylogenetic niche conservatism on community assembly and stability.

This study supports the “disintegration of the ecological community,” wherein macroecological studies cannot (and should not) incorporate interaction into their inferences or conclusions [1]. Studies of community dynamics with spatial components warrant rigorous bases for the incorporation of specific interaction information, or need to acknowledge the uncertainty associated with biotic variables such as NDVI [9,79]. We concur that the idea of cohesive communities as interacting units is incorrect at coarse spatial scales or at the level of most species’ distributions, and that macroecological examinations of community should consider more strongly the importance of coincidental ecological niches in driving patterns of co-occurrence. Future research will hopefully expand upon the general framework presented here, and continue to identify the ways in which species co-occurrence manifests through time.

## 5. Conclusion

It is often easy (and, for macroecological studies, practical) to consider co-occurring species as a “community” for understanding patterns of richness and for identifying potential ecological interactions. However, it is important to understand that every species within a community may be operating under its own unique and independent biogeographic histories, and that communities may simply be a human construct to understand coincidental spatiotemporal co-occurrence. Using our case study of the Costa Rican avifauna, we empirically demonstrate that apparent community cohesion drawn from co-occurrence can also be explained by ecological niche similarity between non-interacting taxa, a pattern that is even more prominent when viewed through a neutral biogeographic lens. Our study underscores the importance of classical ecological studies within co-occurring taxa to truly understand which taxa possess ecological interactions of the magnitude classically associated with ecological communities.

## Supplementary Material

Supplementary Material

## Figures and Tables

**Table 1.** Environmental variables used to create ecological niche models and their derived species distribution models.

Model Layer	Description	Source

Thornthwaite aridity index	Index pertaining to the “degree of water deficit below water need.”	Title & Bemmels 2018
Continentality	The difference in average temperature between the warmest and coldest months.	Title & Bemmels 2018
embergerQ	Emberger’s pluviothermic quotient, a layer that discerns between Mediterranean climates.	Title & Bemmels 2018
Maximum coldest temperature	The maximum temperature during the coldest month.	Title & Bemmels 2018
Minimum warmest temperature	The minimum temperature during the warmest month.	Title & Bemmels 2018
PET driest quarter	The average potential evapotranspiration (PET) during the driest quarter.	Title & Bemmels 2018
PET wettest quarter	The average potential evapotranspiration (PET) during the wettest quarter.	Title & Bemmels 2018
Avg, Max, NDVI	NASA Earth Data, MODIS/Terra Vegetation Indices 16-Day L3 Global 1 km SIN Grid V006 tiles, 2000–2019	urs.earthdata.nasa.gov
Avg. Min, NDVI	NASA Earth Data, MODIS/Terra Vegetation Indices 16-Day L3 Global 1 km SIN Grid V006 tiles, 2000–2019	urs.earthdata.nasa.gov

## Data Availability

Our code files have been uploaded to Github and are available at https://github.com/jacobccooper/costa_rica_community
