# Supplementary material for: What constitutes a community? A co-occurrence exploration of the Costa Rican avifauna
Source: Neotrop Biodivers. Author manuscript; Available in PMC 2023 Jun 2. (PMC10237366; doi:10.1080/23766808.2023.2204549)
Supplement: Supplementary Material [file NIHMS1893357-supplement-Supplementary_Material.zip › Appendix_1.pdf]

Appendix 1: What constitutes a community?

Mélusine F. Velde, Jacob C. Cooper, et al.

23 March 2023

Contents

1 Overview 2
1.1 Background . . . 3
1.2 Required packages . . . 3

2 Ecological Niche Modeling 5

3 Presence-absence matrices 5
3.1 Winter Models . . . 5
Clipping Rasters . . . 8
3.3 Summer models . . . 16
Clipping Rasters . . . 19
3.3 Full unclipped summer models . . . 21
3.4 Full unclipped winter models . . . 24

4 *Ecostructure* analyses: all species 26
4.1 Example run of models . . . 28
Example usage for one level of K . . . 28

5 Creating clusters 28
5.1 Example usage of cluster pipeline . . . 28
December PAM . . . 28
Clustering Code . . . 30

6 Cluster comparisons 36
6.1 Loading models, assigning class . . . 36
Stable Clusters . . . 39
Dispersed Clusters . . . 42
Split Clusters . . . 54
Differences in range between seasons . . . 57
Randomized D Stats . . . 63
Number of points . . . 65

1 Overview

Velde and Cooper are listed as co-first authors and are thus denoted as the main authors for this document. Due to space requirements, the full authorlist has been moved to here. Current (primary) affiliations shown as of March 2023; see publication for more information.

1. Mélusine F. Velde Negaunee Integrative Research Center, Division of Birds Field Museum 1400 S du Sable Lake Shore Drive Chicago, IL 60605 United States
2. Elizabeth M. Besozzi Department of Biology University of Oklahoma 660 Parrington Oval Norman, OK 73019 United States
3. Billi A. Krochuk Biodiversity Research Centre and Dept. of Zoology University of British Columbia 2212 Main Mall Vancouver, BC V6T 1Z4 Canada
4. Kate M. Henderson College of Environmental Science and Forestry State University of New York 1 Forestry Drive Syracuse, NY 13210 United States
5. Brian R. Tsuru School of Environment and Natural Resources Ohio State University 2021 Coffey Road Columbus, OH 43210 United States
6. Sara Velásquez Restrepo Universidad EAFIT Carrera 49 #7 sur 50 Medellín Colombia
7. Holly M. Garrod BirdsCaribbean 841 Worchester Street Natick, MA 01760 United States
8. Jacob C. Cooper Biodiversity Institute & Natural History Museum University of Kansas 1345 Jayhawk Boulevard Lawrence, KS 66045 United States

1.1 Background

This document is a concatenation of codes used to create "What constitutes a community? A co-occurrence exploration of the Costa Rican avifauna". The code was formatted by Jacob C. Cooper and is presented *as is* and will require manipulation by users for use on their own machines. This manuscript relies heavily on data from M. F. Velde's undergraduate thesis at the University of Chicago: "Testing the accuracy of species distribution models based on community science data". These models were created using minimum volume ellipsoids (MVEs) and will be made available in a future publication.

Please note that many of these analyses utilize algorithms that change with each iteration; thus, results presented herein may not match exactly with those in the manuscript.

1.2 Required packages

Please note that due to constant updates, these may not be the exact version numbers used in the manuscript.

```
library(ape)
library(data.table)
library(dismo)
```

```
## Loading required package: raster
```

```
## Loading required package: sp
```

```
library(ecostructure)
```

```
## Loading required package: ggplot2
```

```
library(factoextra)
```

```
## Welcome! Want to learn more? See two factoextra-related books at https://goo.gl/ve3WBa
```

```
library(ggplot2)
library(gridExtra)
library(raster)
library(rgdal)
```

```
## Please note that rgdal will be retired during 2023,
## plan transition to sf/stars/terra functions using GDAL and PROJ
## at your earliest convenience.
## See https://r-spatial.org/r/2022/04/12/evolution.html and https://github.com/r-spatial/evolution
## rgdal: version: 1.6-5, (SVN revision 1199)
## Geospatial Data Abstraction Library extensions to R successfully loaded
## Loaded GDAL runtime: GDAL 3.4.1, released 2021/12/27
## Path to GDAL shared files: /usr/share/gdal
## GDAL binary built with GEOS: TRUE
## Loaded PROJ runtime: Rel. 8.2.1, January 1st, 2022, [PJ_VERSION: 821]
## Path to PROJ shared files: /home/jccooper/.local/share/proj:/usr/share/proj
## PROJ CDN enabled: FALSE
## Linking to sp version:1.6-0
## To mute warnings of possible GDAL/OSR exportToProj4() degradation,
## use options("rgdal_show_exportToProj4_warnings"="none") before loading sp or rgdal.
```

```
library(rgeos)
```

```
## rgeos version: 0.6-2, (SVN revision 693)
##  GEOS runtime version: 3.10.2-CAPI-1.16.0
##  Please note that rgeos will be retired during 2023,
## plan transition to sf functions using GEOS at your earliest convenience.
```

```
##  GEOS using OverlayNG
##  Linking to sp version: 1.6-0
##  Polygon checking: TRUE
```

```
library(rnaturalearth)
library(sf)
```

```
## Linking to GEOS 3.10.2, GDAL 3.4.1, PROJ 8.2.1; sf_use_s2() is TRUE
```

```
library(tidyverse)
```

```
## -- Attaching core tidyverse packages ------------------------ tidyverse 2.0.0 --
## v dplyr     1.1.1     v readr     2.1.4
## v forcats   1.0.0     v stringr   1.5.0
## v lubridate 1.9.2     v tibble    3.2.1
## v purrr     1.0.1     v tidyr     1.3.0
```

```
## -- Conflicts ------------------------------------------ tidyverse_conflicts() --
## x dplyr::between()     masks data.table::between()
## x dplyr::combine()     masks gridExtra::combine()
## x tidyr::extract()     masks raster::extract()
## x dplyr::filter()      masks stats::filter()
## x dplyr::first()       masks data.table::first()
## x lubridate::hour()    masks data.table::hour()
## x lubridate::isoweek() masks data.table::isoweek()
## x dplyr::lag()         masks stats::lag()
## x dplyr::last()        masks data.table::last()
## x lubridate::mday()    masks data.table::mday()
## x lubridate::minute()  masks data.table::minute()
## x lubridate::month()   masks data.table::month()
## x lubridate::quarter() masks data.table::quarter()
## x lubridate::second()  masks data.table::second()
## x dplyr::select()      masks raster::select()
## x dplyr::symdiff()     masks rgeos::symdiff()
## x purrr::transpose()   masks data.table::transpose()
## x lubridate::wday()    masks data.table::wday()
## x lubridate::week()    masks data.table::week()
## x dplyr::where()       masks ape::where()
## x lubridate::yday()    masks data.table::yday()
## x lubridate::year()    masks data.table::year()
## i Use the conflicted package (<http://conflicted.r-lib.org/>) to force all conflicts to become error
```

```
library(vegan)
```

```
## Loading required package: permute
## Loading required package: lattice
## This is vegan 2.6-4
```

```
# not available for updated R
# used in this analysis
# library(velox)

library(viridis)
```

```
## Loading required package: viridisLite
```

2 Ecological Niche Modeling

This study utilizes ecological niche models (ENMs) created by MF Velde for her undergraduate thesis project at the University of Chicago (Velde 2021). These models use minimum volume ellipsoids (MVEs) to create suitability outputs and apply varying thresholds to the data. These data were created using two different datasets, that had two 10 km radius artificial absences in each to test their effectiveness at modeling species distributions (Velde 2021).

3 Presence-absence matrices

The following is an overview of the pipeline used to create the presence absence matrix derived from MFV's data.

3.1 Winter Models

The MVE models were created, trained, and projected to the entirety of Costa Rica and Panama. For this study, we are explicitly looking at Costa Rica, given the density of data and how well known the bird communities are in the country. Additionally, the MVE models were projected out without regard to biogeographic barriers. Here, we are correcting for these changes and ensuring that all species are restricted to their appropriate biogeographic zones to create more accurate species distribution models from which other metrics can be calculated.

In this particular study, we used only two biogeographic regions - Pacific Slope and Caribbean Slope - as our preliminary explorations demonstrated that further subdivisions biased *ecostructure* outputs.

```
# get list of files with points

gpkgs=list.files(shp.filepath,pattern=".gpkg")

y=readOGR(paste0(shp.filepath,gpkgs[2]))
```

```
## Warning: OGR support is provided by the sf and terra packages among others

## Warning: OGR support is provided by the sf and terra packages among others

## Warning: OGR support is provided by the sf and terra packages among others

## Warning: OGR support is provided by the sf and terra packages among others

## Warning: OGR support is provided by the sf and terra packages among others

## Warning: OGR support is provided by the sf and terra packages among others

## Warning: OGR support is provided by the sf and terra packages among others

## OGR data source with driver: GPKG
## Source: "/home/jccooper/Dropbox/motmots/shapefiles/Pacific.gpkg", layer: "Pacific"
## with 1 features
## It has 0 fields
```

```
crs.x=crs(y)

plot(y)
```

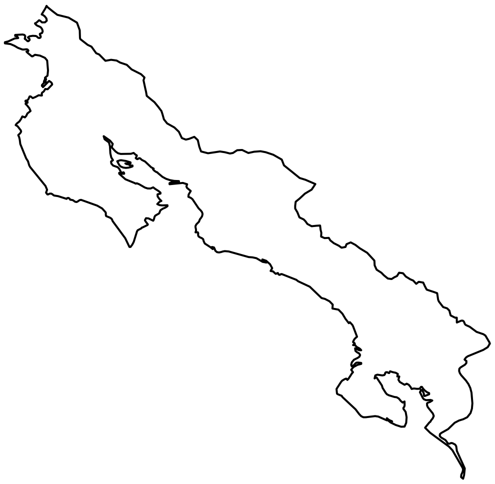

```r
# We create training areas based on winter data for most
# winter is the time of dispersal and vagrancy for many taxa
# summer species not dealt with here are dealt with later.

species=list.files(paste0(sdm.filepath,
                          "all.species"),
                   pattern="*.csv")
```

```r
# preload shapefiles since we have only four

y1=readOGR(paste0(shp.filepath,gpkgs[1]))
y2=readOGR(paste0(shp.filepath,gpkgs[2]))
#y3=readOGR(paste0(shp.filepath,gpkgs[3]))
#y4=readOGR(paste0(shp.filepath,gpkgs[4]))

for(i in 1:length(species)){
  x=read.csv(paste0(sdm.filepath,"all.species/",species[i]))
  x2=x%>%dplyr::select(LONGITUDE,LATITUDE)
  x.data2=SpatialPoints(x2,proj4string = crs.x)

  shp.list=NULL

  # for variable number of gpkgs files

  #for(k in 1:length(gpkgs)){
  #  y=readOGR(paste0(shp.filepath,gpkgs[k]))
```

```
#   inside.m=sum(!is.na(over(x.data2,as(y,"SpatialPolygons"))))
#   if(inside.m>0){shp.list[k]=1}else{shp.list[k]=0}
#}

# for this instance, since there are only five

shp.list[1]=sum(!is.na(over(x.data2,as(y1,"SpatialPolygons"))))
shp.list[2]=sum(!is.na(over(x.data2,as(y2,"SpatialPolygons"))))
#shp.list[3]=sum(!is.na(over(x.data2,as(y3,"SpatialPolygons"))))
#shp.list[4]=sum(!is.na(over(x.data2,as(y4,"SpatialPolygons"))))

shps=which(shp.list>0)

if(length(shps)==0){next}

if(length(shps)==1){
  # x.u.1=readOGR(paste0(shp.filepath,gpkgs[shps]))
  if(shps==1){assign('x.u.1',y1)}
  if(shps==2){assign('x.u.1',y2)}
  #if(shps==3){assign('x.u.1',y3)}
  #if(shps==4){assign('x.u.1',y4)}
}else{
  # merge shapefiles
  #x.u.1=readOGR(paste0(shp.filepath,gpkgs[shps[1]]))
  #for(k in 2:length(shps)){
  #   x.u.2=readOGR(paste0(shp.filepath,gpkgs[shps[k]]))
  #   x.u.1=gUnion(x.u.1,x.u.2)
  #}

  # memory saver below
  for(k in 1:length(shps)){
    if(k==1){
      if(shps[k]==1){assign('x.u.1',y1)}
      if(shps[k]==2){assign('x.u.1',y2)}
      #if(shps[k]==3){assign('x.u.1',y3)}
      #if(shps[k]==4){assign('x.u.1',y4)}
      #if(shps[k]==5){assign('x.u.1',y5)}
    }else{
      if(shps[k]==1){assign('x.u.2',y1)}
      if(shps[k]==2){assign('x.u.2',y2)}
      #if(shps[k]==3){assign('x.u.2',y3)}
      #if(shps[k]==4){assign('x.u.2',y4)}
      #if(shps[k]==5){assign('x.u.2',y5)}

      x.u.1=gUnion(x.u.1,x.u.2)
    }
  }
}

split.name=strsplit(species[i],"[.]")[[1]][1]

x.union=st_as_sf(x.u.1)
```

```
  st_write(x.union,
           paste0(shp.filepath,"training_areas/",split.name,".gpkg"),
           split.name)
}

print('done')
```

Clipping Rasters

Next, we need to clip all rasters to the aforementioned training areas and ensure that we have the proper biogeographic envelopes applied for each species.

Note the SDM subdirectories are `output_all/75` for the whole files thresholded with 75% confidence and `clipped_SDM` for the clipped rasters.

```
sdm.list=list.files(paste0(sdm.path,"output_all/75"),pattern="*.tif")
shp.list=list.files(paste0(shp.path),pattern="*.gpkg")
```

We will not be clipping training areas for each species to correct for species richness patterns etc.

```
## Warning: OGR support is provided by the sf and terra packages among others

## Warning: OGR support is provided by the sf and terra packages among others

## Warning: OGR support is provided by the sf and terra packages among others

## Warning: OGR support is provided by the sf and terra packages among others

## Warning: OGR support is provided by the sf and terra packages among others

## Warning: OGR support is provided by the sf and terra packages among others

## Warning: OGR support is provided by the sf and terra packages among others

## OGR data source with driver: GPKG
## Source: "/home/jccooper/Dropbox/motmots/shapefiles/training_areas/Acanthidops-bairdi.gpkg", layer: "
## with 1 features
## It has 0 fields
```

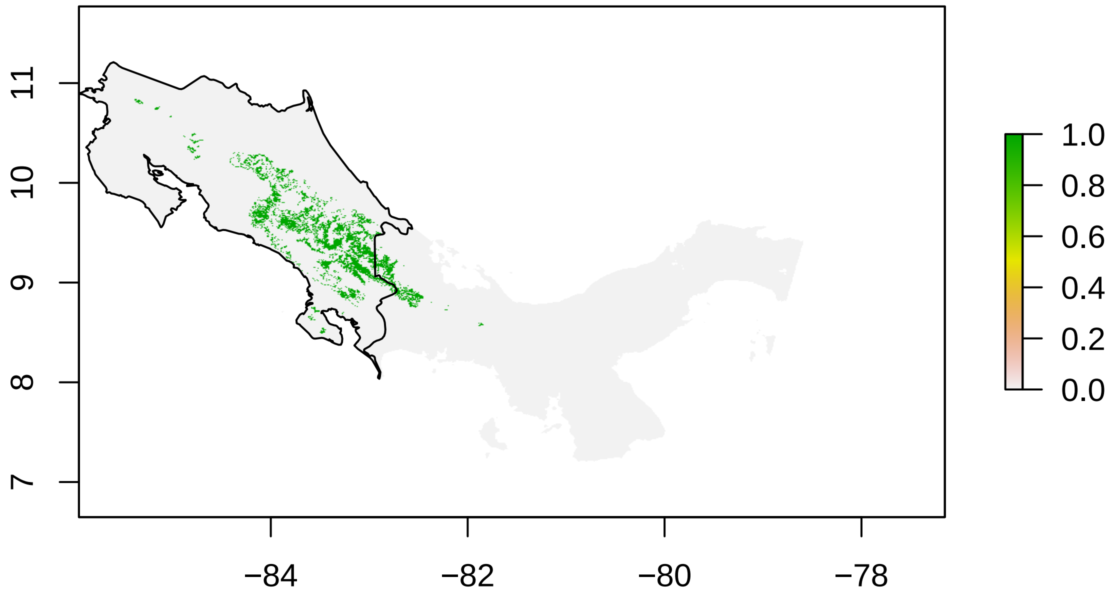

Above is an illustration of , where the areas where points have been recorded are highlighted in black, and areas outside of the Costa Rica training area or where the species truly does not occur are outside of this biogeographic envelope.

In order to ensure everything is cropped correctly, we will use a 'reference raster' from a widespread species.

```
## Warning: OGR support is provided by the sf and terra packages among others
```

```
## Warning: OGR support is provided by the sf and terra packages among others
```

```
## Warning: OGR support is provided by the sf and terra packages among others
```

```
## Warning: OGR support is provided by the sf and terra packages among others
```

```
## Warning: OGR support is provided by the sf and terra packages among others
```

```
## Warning: OGR support is provided by the sf and terra packages among others
```

```
## Warning: OGR support is provided by the sf and terra packages among others
```

```
## OGR data source with driver: GPKG
## Source: "/home/jccooper/Dropbox/motmots/shapefiles/training_areas/Amazilia-tzacatl.gpkg", layer: "Ama
## with 1 features
## It has 0 fields
```

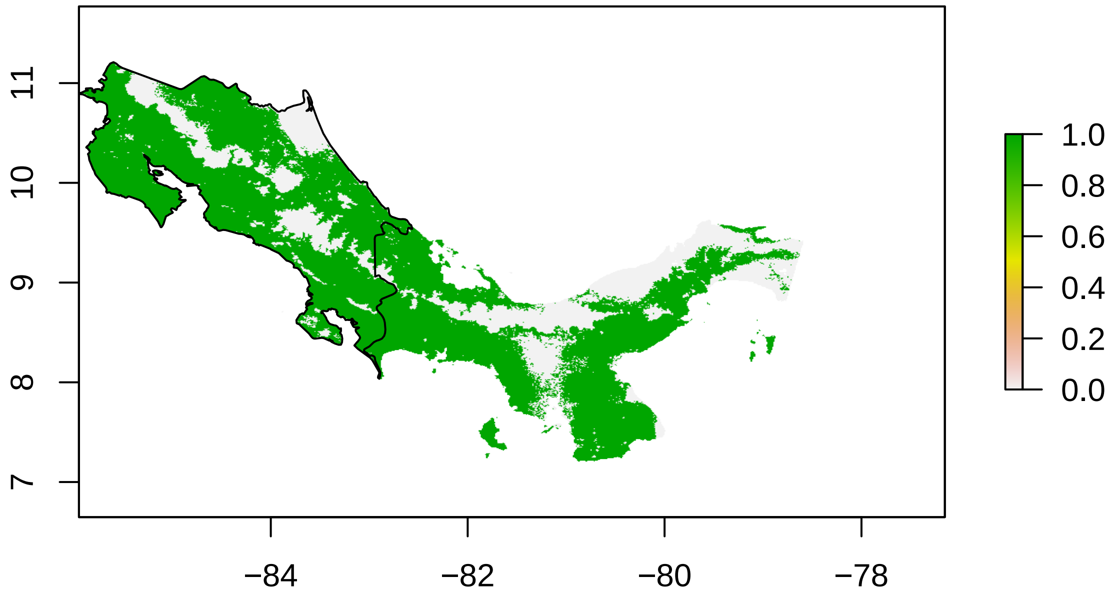

Above is *Amazilia tzacatl*, a widespread Neotropical hummingbird, as predicted by the ecological niche models.Since it occurs in every part of Costa Rica, we can use it to create a default projection raster.

```
# creating template from Amazilia

cr1=crop(x,y)
cr2=raster::mask(cr1,y)
crs(cr2)=crs(x.temp)
cr3=projectRaster(cr2,x.temp,method='ngb',
                  res=(res(x)),crs=crs(x))

cr3[cr3>0]=0

x.temp=cr3

plot(cr3)
```

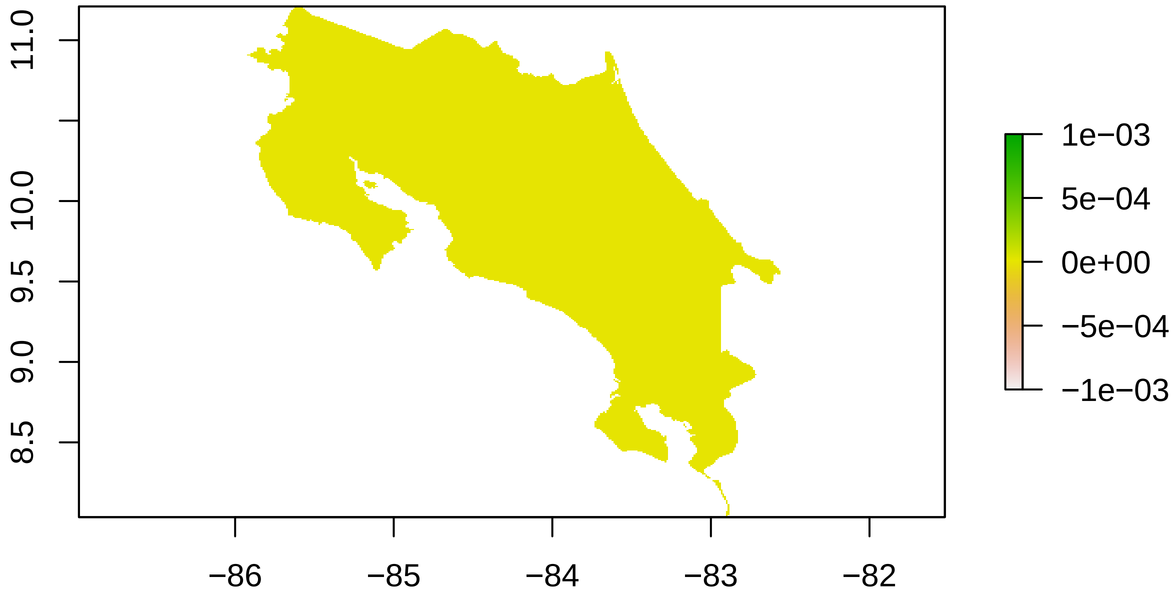

We can see here that the template extent works. No we can start clipping everything. The template extent importantly has zero values for the entire land, so stacking and summing will create maps that cover the entire country.

Furthermore, we want to create hexbins of everything so we can perform ecostructure analyses.

Important note: due to issues with the hex polygons and classes I did all of this in memory at the same time.

```
# create hexbin over Zonotrichia training
y2=as(y,"Spatial")
CR.buff=gBuffer(y,width=0.05)
CR.hex=spsample(CR.buff,type='hexagonal',cellsize=0.125)
hex=HexPoints2SpatialPolygons(CR.hex)

Pam.coords=as.data.frame(CR.hex@coords)
Locality=paste0("ID",1:nrow(Pam.coords))

locality_metadata=cbind(Locality,Pam.coords)
colnames(locality_metadata)=c('Locality','Longitude','Latitude')
write.csv(locality_metadata,paste0(sdm.path,"locality_metadata.csv"),
          quote=F,row.names = F)

sf.hex=st_as_sf(CR.hex)
```

```
plot(y)
plot(hex,add=T)
```

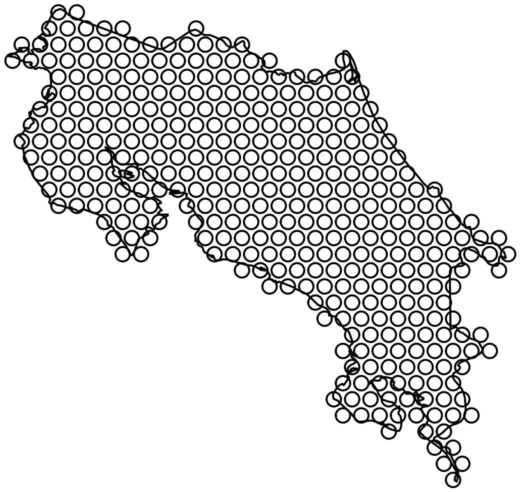

Now we have a hex grid created. Our goals are to create 1) a biogeographic SDM and 2) a hex grid representation of that SDM for the creation of our PAM.

```
## Warning: OGR support is provided by the sf and terra packages among others

## Warning: OGR support is provided by the sf and terra packages among others

## Warning: OGR support is provided by the sf and terra packages among others

## Warning: OGR support is provided by the sf and terra packages among others

## Warning: OGR support is provided by the sf and terra packages among others

## Warning: OGR support is provided by the sf and terra packages among others

## Warning: OGR support is provided by the sf and terra packages among others

## OGR data source with driver: GPKG
## Source: "/home/jccooper/Dropbox/motmots/SDMs/hex_mesh_slopes.gpkg", layer: "hex_mesh_slopes"
## with 372 features
## It has 0 fields

## class       : SpatialPointsDataFrame
## features    : 372
## extent      : -85.9219, -82.5469, 8.046986, 11.18633  (xmin, xmax, ymin, ymax)
## crs         : +proj=longlat +datum=WGS84 +no_defs
## variables   : 1
## names       : FID
## min values  :   1
## max values  : 372
```

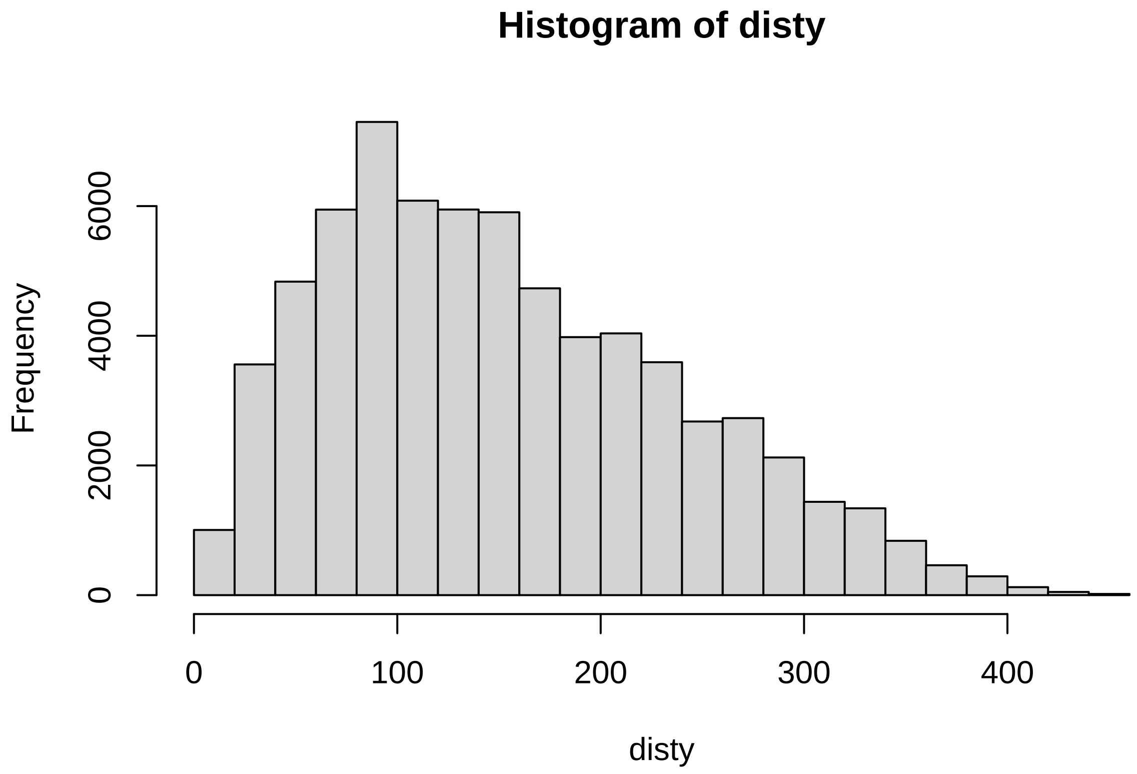


```
## [1] 13.65229

## [1] 645.6569
```

```
PAM=NULL

errors="Errors"

for(i in 412:length(sdm.list)){
  name=strsplit(sdm.list[i],"_")[[1]][1]

  if(length(which(shp.list%flike%name))<1){
    errors=c(errors,name)
    next
  }
  if(length(which(sdm.list%flike%name))<1){
    errors=c(errors,name)
    next
  }

  x=raster(paste0(sdm.path,"output_all/75/",sdm.list[i]))
  y=readOGR(paste0(shp.path,shp.list[which(shp.list%like%name)]))

  cr1=crop(x,y)
  cr2=raster::mask(cr1,y)
  crs(cr2)=crs(x.temp)
  cr3=projectRaster(cr2,x.temp,method='ngb',
```

```
                     res=(res(x)),crs=crs(x))

# make everything zero so land cells become 0 when stacked
cr3[is.na(cr3)]=0

x.stack=stack(cr3,x.temp)
cr4=calc(x.stack,fun=sum)

# performed in previous iteration
if(file.exists(paste0(sdm.path,'clipped_SDM/',
                            name,"_cropped.tif"))==F){
  writeRaster(cr4,
            filename=paste0(sdm.path,'clipped_SDM/',
                            name,"_cropped.tif"))
}


# project to hex
rm(x)
rm(y)
rm(cr1)
rm(cr2)
rm(cr3)
rm(cr4)

v.cr=velox(paste0(sdm.path,'clipped_SDM/',
                            name,"_cropped.tif"))

hex.ext=v.cr$extract(hex)

#hex.ext=raster::extract(cr4,hex)

hex.vals=NULL

for(k in 1:length(hex.ext)){
  n=length(na.omit(hex.ext[[k]]))
  j=sum(na.omit(hex.ext[[k]]))
  if(j==0){
    hex.vals[k]=0
    next
  }
  if(n<11){
    if(j>0.7){hex.vals[k]=1}else{hex.vals[k]=0}
    next
  }
  if(n<41){
    if(j>0.5){hex.vals[k]=1}else{hex.vals[k]=0}
    next
  }
  if(n>40){
    if(j>0.3){hex.vals[k]=1}else{hex.vals[k]=0}
    next
  }
```

```r
  }

  hex.data=as.data.frame(hex.vals)
  row.names(hex.data)=paste0("ID",1:length(hex))

  hex.data=SpatialPolygonsDataFrame(hex,hex.data)

  hex.write=st_as_sf(hex.data)

  st_write(hex.write,
           paste0(sdm.filepath,"hex/",name,".gpkg"))

  if(i==1){
    PAM=as.data.frame(hex.vals)
    colnames(PAM)[i]=name
  }else{
    PAM=cbind(PAM,hex.vals)
    index=which(colnames(PAM)=="hex.vals")
    colnames(PAM)[index]=name
  }
}

print('done')
```

```r
print(errors)

row.names(PAM)=paste0('ID',1:nrow(PAM))
PAM2=t(PAM)

write.csv(PAM,paste0(sdm.path,"eco_PAM.csv"),row.names = T,quote=F)

print("done")
```

```
 [1] "Errors"                        "Amazona-ochrocephala"
 [3] "Anthracothorax-nigricollis" "Ardea-cocoi"
 [5] "Atalotriccus-pilaris"       "Cacicus-cela"
 [7] "Campephilus-melanoleucos"   "Cantorchilus-leucotis"
 [9] "Cercomacra-nigricans"       "Chaetura-brachyura"
[11] "Chaetura-spinicaudus"       "Chalybura-buffonii"
[13] "Cotinga-nattererii"         "Crotophaga-major"
[15] "Dendroplex-picus"           "Euphonia-fulvicrissa"
[17] "Glaucis-hirsutus"           "Icterus-chrysater"
[19] "Juliamyia-julie"            "Lophornis-delattrei"
[21] "Manacus-vitellinus"         "Momotus-subrufescens"
[23] "Myiopagis-gaimardii"        "Myrmeciza-longipes"
[25] "Notharchus-pectoralis"      "Oncostoma-olivaceum"
[27] "Pachysylvia-aurantiifrons"  "Pitangus-lictor"
[29] "Ramphocelus-dimidiatus"     "Rhynchocyclus-olivaceus"
[31] "Sicalis-flaveola"           "Trogon-chionurus"
[33] "Trogon-melanurus"
[1] "done"
```

3.3 Summer models

```
# get list of files with points

gpkgs=list.files(shp.filepath,pattern=".gpkg")

y=readOGR(paste0(shp.filepath,gpkgs[2]))

crs.x=crs(y)
```

```
species=list.files(paste0(sdm.filepath,
                          "all.species"),
                   pattern="*.csv")
```

```
# preload shapefiles since we have only five

y1=readOGR(paste0(shp.filepath,gpkgs[1]))
y2=readOGR(paste0(shp.filepath,gpkgs[2]))
#y3=readOGR(paste0(shp.filepath,gpkgs[3]))
#y4=readOGR(paste0(shp.filepath,gpkgs[4]))
#y5=readOGR(paste0(shp.filepath,gpkgs[5]))

new.files="new.files"

for(i in 1:length(species)){
  x=read.csv(paste0(sdm.filepath,"all.species/",species[i]))

  split.name=strsplit(species[i],"[.]")[[1]][1]

  if(file.exists(paste0(shp.filepath,
                  "training_areas/",split.name,".gpkg"))==T){
      next
    }else{
      new.files=c(new.files,split.name)
    }

  x2=x%>%dplyr::select(LONGITUDE,LATITUDE)
  x.data2=SpatialPoints(x2,proj4string = crs.x)

  shp.list=NULL

  # for variable number of gpkgs files

  #for(k in 1:length(gpkgs)){
  #  y=readOGR(paste0(shp.filepath,gpkgs[k]))
  #  inside.m=sum(!is.na(over(x.data2,as(y,"SpatialPolygons"))))
  #  if(inside.m>0){shp.list[k]=1}else{shp.list[k]=0}
  #}

  # for this instance, since there are only five

  shp.list[1]=sum(!is.na(over(x.data2,as(y1,"SpatialPolygons"))))
  shp.list[2]=sum(!is.na(over(x.data2,as(y2,"SpatialPolygons"))))
  #shp.list[3]=sum(!is.na(over(x.data2,as(y3,"SpatialPolygons"))))
```

```r
  #shp.list[4]=sum(!is.na(over(x.data2,as(y4,"SpatialPolygons"))))
  #shp.list[5]=sum(!is.na(over(x.data2,as(y5,"SpatialPolygons"))))

  shps=which(shp.list>0)

  if(length(shps)==0){next}

  if(length(shps)==1){
    # x.u.1=readOGR(paste0(shp.filepath,gpkgs[shps]))
    if(shps==1){assign('x.u.1',y1)}
    if(shps==2){assign('x.u.1',y2)}
    #if(shps==3){assign('x.u.1',y3)}
    #if(shps==4){assign('x.u.1',y4)}
    #if(shps==5){assign('x.u.1',y5)}
  }else{
    # merge shapefiles
    #x.u.1=readOGR(paste0(shp.filepath,gpkgs[shps[1]]))
    #for(k in 2:length(shps)){
    #   x.u.2=readOGR(paste0(shp.filepath,gpkgs[shps[k]]))
    #   x.u.1=gUnion(x.u.1,x.u.2)
    #}

    # memory saver below
    for(k in 1:length(shps)){
      if(k==1){
        if(shps[k]==1){assign('x.u.1',y1)}
        if(shps[k]==2){assign('x.u.1',y2)}
        #if(shps[k]==3){assign('x.u.1',y3)}
        #if(shps[k]==4){assign('x.u.1',y4)}
        #if(shps[k]==5){assign('x.u.1',y5)}
      }else{
        if(shps[k]==1){assign('x.u.2',y1)}
        if(shps[k]==2){assign('x.u.2',y2)}
        #if(shps[k]==3){assign('x.u.2',y3)}
        #if(shps[k]==4){assign('x.u.2',y4)}
        #if(shps[k]==5){assign('x.u.2',y5)}

        x.u.1=gUnion(x.u.1,x.u.2)
      }
    }
  }

  x.union=st_as_sf(x.u.1)

  st_write(x.union,
           paste0(shp.filepath,"training_areas/",split.name,".gpkg"),
           split.name)
}

print(new.files)
```

```
  [1] "new.files"                    "Amazona-ochrocephala"
  [3] "Androdon-aequatorialis"       "Anthracothorax-nigricollis"
  [5] "Anthus-lutescens"             "Aphanotriccus-audax"
```

```
  [7] "Ara-chloropterus"              "Ara-severus"
  [9] "Ardea-cocoi"                   "Ardenna-grisea"
 [11] "Arremon-atricapillus"          "Atalotriccus-pilaris"
 [13] "Atlapetes-luteoviridis"        "Atticora-tibialis"
 [15] "Basileuterus-ignotus"          "Brachygalba-salmoni"
 [17] "Cacicus-cela"                  "Calidris-fuscicollis"
 [19] "Campephilus-haematogaster"     "Campephilus-melanoleucos"
 [21] "Campylorhynchus-albobrunneus"  "Campylorhynchus-griseus"
 [23] "Cantorchilus-leucopogon"       "Cantorchilus-leucotis"
 [25] "Capito-maculicoronatus"        "Carpodectes-hopkei"
 [27] "Caryothraustes-canadensis"     "Ceratopipra-erythrocephala"
 [29] "Cercomacra-nigricans"          "Chaetura-brachyura"
 [31] "Chaetura-spinicaudus"          "Chalybura-buffonii"
 [33] "Chlorospingus-flavigularis"    "Chlorospingus-inornatus"
 [35] "Chlorospingus-tacarcunae"      "Chlorothraupis-olivacea"
 [37] "Chrysomus-icterocephalus"      "Circus-buffoni"
 [39] "Cnipodectes-subbrunneus"       "Coccycua-minuta"
 [41] "Colaptes-punctigula"           "Conirostrum-leucogenys"
 [43] "Cotinga-nattererii"            "Cranioleuca-dissita"
 [45] "Crotophaga-major"              "Cryptoleucopteryx-plumbea"
 [47] "Cryptopipo-holochlora"         "Cyanerpes-caeruleus"
 [49] "Dacnis-viguieri"               "Dendroplex-picus"
 [51] "Donacobius-atricapilla"        "Euphonia-fulvicrissa"
 [53] "Euphonia-xanthogaster"         "Fluvicola-pica"
 [55] "Forpus-conspicillatus"         "Glaucis-hirsutus"
 [57] "Goethalsia-bella"              "Goldmania-violiceps"
 [59] "Gygis-alba"                    "Haplophaedia-aureliae"
 [61] "Harpia-harpyja"                "Hemithraupis-flavicollis"
 [63] "Herpsilochmus-rufimarginatus"  "Heterospingus-xanthopygius"
 [65] "Icterus-auricapillus"          "Icterus-chrysater"
 [67] "Jacamerops-aureus"             "Juliamyia-julie"
 [69] "Larosterna-inca"               "Larus-californicus"
 [71] "Larus-delawarensis"            "Larus-dominicanus"
 [73] "Larus-fuscus"                  "Leucophaeus-modestus"
 [75] "Lophornis-delattrei"           "Machetornis-rixosa"
 [77] "Manacus-vitellinus"            "Margarornis-bellulus"
 [79] "Momotus-subrufescens"          "Morphnus-guianensis"
 [81] "Myadestes-coloratus"           "Myiodynastes-chrysocephalus"
 [83] "Myiopagis-caniceps"            "Myiopagis-gaimardii"
 [85] "Myrmeciza-longipes"            "Myrmornis-torquata"
 [87] "Myrmotherula-ignota"           "Myrmotherula-pacifica"
 [89] "Nonnula-frontalis"             "Notharchus-pectoralis"
 [91] "Nystalus-radiatus"             "Oncostoma-olivaceum"
 [93] "Pachyramphus-homochrous"       "Pachyramphus-rufus"
 [95] "Pachysylvia-aurantiifrons"     "Patagioenas-leucocephala"
 [97] "Patagioenas-plumbea"           "Pelecanus-erythrorhynchos"
 [99] "Phaethornis-anthophilus"       "Phaetusa-simplex"
[101] "Philydor-fuscipenne"           "Phyllomyias-griseiceps"
[103] "Phylloscartes-flavovirens"     "Piculus-callopterus"
[105] "Piculus-chrysochloros"         "Pilherodius-pileatus"
[107] "Pitangus-lictor"               "Poecilostreptus-palmeri"
[109] "Polioptila-schistaceigula"     "Progne-elegans"
[111] "Psarocolius-guatimozinus"      "Pseudobulweria-rostrata"
[113] "Pyrrhura-picta"                "Quiscalus-lugubris"
```

```
[115] "Ramphocelus-dimidiatus"       "Rhynchocyclus-olivaceus"
[117] "Sapayoa-aenigma"              "Schiffornis-stenorhyncha"
[119] "Selasphorus-ardens"           "Sicalis-flaveola"
[121] "Sicalis-luteola"              "Sirystes-albogriseus"
[123] "Sula-granti"                  "Tangara-fucosa"
[125] "Tersina-viridis"              "Thamnophilus-nigriceps"
[127] "Tolmomyias-flaviventris"      "Touit-dilectissimus"
[129] "Trogon-chionurus"             "Trogon-melanurus"
[131] "Vireolanius-eximius"          "Xenerpestes-minlosi"
[133] "Zentrygon-goldmani"
```

Some of the above are also just species that occur in Panama that are not in Costa Rica; therefore, not fully accurate.

Clipping Rasters

Next, we need to clip all rasters to the aforementioned training areas and ensure that we have the proper biogeographic envelopes applied for each species.

Note the SDM subdirectories are `output_all/75` for the whole files thresholded with 75% confidence and `clipped_SDM` for the clipped rasters.

```
sdm.list=list.files(paste0(sdm.path,"output/75"),pattern="*.tif")
shp.list=list.files(paste0(shp.path),pattern="*.gpkg")
```

We will not be clipping training areas for each species to correct for species richness patterns etc.

```
PAM=NULL

errors="Errors"

for(i in 1:length(sdm.list)){
  name=strsplit(sdm.list[i],"_")[[1]][1]

  if(length(which(shp.list%flike%name))<1){
    errors=c(errors,name)
    next
  }
  if(length(which(sdm.list%flike%name))<1){
    errors=c(errors,name)
    next
  }

  x=raster(paste0(sdm.path,"output/75/",sdm.list[i]))
  y=readOGR(paste0(shp.path,shp.list[which(shp.list%like%name)]))

  cr1=crop(x,y)
  cr2=raster::mask(cr1,y)
  crs(cr2)=crs(x.temp)
  cr3=projectRaster(cr2,x.temp,method='ngb',
                    res=(res(x)),crs=crs(x))

  # make everything zero so land cells become 0 when stacked
  cr3[is.na(cr3)]=0

  x.stack=stack(cr3,x.temp)
```

```r
cr4=calc(x.stack,fun=sum)

# performed in previous iteration
if(file.exists(paste0(sdm.path,'clipped_SDM/',
                           name,"_cropped.tif"))==F){
  writeRaster(cr4,
            filename=paste0(sdm.path,'clipped_SDM/',
                           name,"_cropped.tif"))
}

# project to hex
# project to hex
rm(x)
rm(y)
rm(cr1)
rm(cr2)
rm(cr3)
rm(cr4)

v.cr=velox(paste0(sdm.path,'clipped_SDM/',
                           name,"_cropped.tif"))

hex.ext=v.cr$extract(hex)

#hex.ext=raster::extract(cr4,hex)

hex.vals=NULL

for(k in 1:length(hex.ext)){
  n=length(na.omit(hex.ext[[k]]))
  j=sum(na.omit(hex.ext[[k]]))
  if(j==0){
    hex.vals[k]=0
    next
  }
  if(n<11){
    if(j>0.7){hex.vals[k]=1}else{hex.vals[k]=0}
    next
  }
  if(n<41){
    if(j>0.5){hex.vals[k]=1}else{hex.vals[k]=0}
    next
  }
  if(n>40){
    if(j>0.3){hex.vals[k]=1}else{hex.vals[k]=0}
    next
  }
}

hex.data=as.data.frame(hex.vals)
row.names(hex.data)=paste0("ID",1:length(hex))

hex.data=SpatialPolygonsDataFrame(hex,hex.data)
```

```
  hex.write=st_as_sf(hex.data)

  st_write(hex.write,
           paste0(sdm.filepath,"hex/",name,".gpkg"))

  #st_write(hex.data,
  #          paste0(shp.filepath,"training_areas/",split.name,".gpkg"),
  #          split.name)
  #writeOGR(hex.data,
  #          dsn=paste0(sdm.path,"hex/",name,"_hex-grid.gpkg"),
  #          layer=name,
  #          driver="SQLite")

  if(i==1){
    PAM=as.data.frame(hex.vals)
    colnames(PAM)[i]=name
  }else{
    PAM=cbind(PAM,hex.vals)
    index=which(colnames(PAM)=="hex.vals")
    colnames(PAM)[index]=name
  }
}

print("Done.")
```

```
print(errors)

row.names(PAM)=paste0('ID',1:nrow(PAM))
PAM2=t(PAM)

write.csv(PAM,paste0(sdm.path,"eco_PAM.csv"),row.names = T,quote=F)
```

```
 [1] "Errors"                      "Amazona-ochrocephala"
 [3] "Atalotriccus-pilaris"        "Cacicus-cela"
 [5] "Campephilus-melanoleucos"    "Cantorchilus-leucotis"
 [7] "Chaetura-brachyura"          "Chaetura-spinicaudus"
 [9] "Chalybura-buffonii"          "Crotophaga-major"
[11] "Dendroplex-picus"            "Euphonia-fulvicrissa"
[13] "Glaucis-hirsutus"            "Icterus-chrysater"
[15] "Juliamyia-julie"             "Manacus-vitellinus"
[17] "Momotus-subrufescens"        "Myiopagis-gaimardii"
[19] "Myrmeciza-longipes"          "Notharchus-pectoralis"
[21] "Oncostoma-olivaceum"         "Pachysylvia-aurantiifrons"
[23] "Pitangus-lictor"             "Ramphocelus-dimidiatus"
[25] "Sicalis-flaveola"            "Trogon-chionurus"
[27] "Trogon-melanurus"
```

3.3 Full unclipped summer models

```
sdm.path="~/Dropbox/motmots/SDMs/SanJose_june/"

sdm.list=list.files(paste0(sdm.path,"output/75"),pattern="*.tif")
```

```
PAM=NULL

errors="Errors"

for(i in 1:length(sdm.list)){
  name=strsplit(sdm.list[i],"_")[[1]][1]

  if(length(which(shp.list%flike%name))<1){
    errors=c(errors,name)
    next
  }
  if(length(which(sdm.list%flike%name))<1){
    errors=c(errors,name)
    next
  }

  #x=raster(paste0(sdm.path,"output_all/75/",sdm.list[i]))
  #y=readOGR(paste0(shp.path,shp.list[which(shp.list%like%name)]))

  #cr1=crop(x,y)
  #cr2=raster::mask(cr1,y)
  #crs(cr2)=crs(x.temp)
  #cr3=projectRaster(cr2,x.temp,method='ngb',
  #                  res=(res(x)),crs=crs(x))

  # make everything zero so land cells become 0 when stacked
  #cr3[is.na(cr3)]=0

  #x.stack=stack(cr3,x.temp)
  #cr4=calc(x.stack,fun=sum)

  #writeRaster(cr4,
  #            filename=paste0(sdm.path,'clipped_SDM/',
  #                            name,"_cropped.tif"))

  # project to hex
  # project to hex
  #rm(x)
  #rm(y)
  #rm(cr1)
  #rm(cr2)
  #rm(cr3)
  #rm(cr4)

  v.cr=velox(paste0(sdm.path,'output/75/',
                              sdm.list[i]))

  hex.ext=v.cr$extract(hex)

  #hex.ext=raster::extract(cr4,hex)

  hex.vals=NULL
```

```
  for(k in 1:length(hex.ext)){
    n=length(na.omit(hex.ext[[k]]))
    j=sum(na.omit(hex.ext[[k]]))
    if(j==0){
      hex.vals[k]=0
      next
    }
    if(n<11){
      if(j>0.7){hex.vals[k]=1}else{hex.vals[k]=0}
      next
    }
    if(n<41){
      if(j>0.5){hex.vals[k]=1}else{hex.vals[k]=0}
      next
    }
    if(n>40){
      if(j>0.3){hex.vals[k]=1}else{hex.vals[k]=0}
      next
    }
  }

  hex.data=as.data.frame(hex.vals)
  row.names(hex.data)=paste0("ID",1:length(hex))

  hex.data=SpatialPolygonsDataFrame(hex,hex.data)

  #hex.write=st_as_sf(hex.data)

  st_write(hex.write,
           paste0(sdm.filepath,"noclip/",name,".gpkg"))

  #st_write(hex.data,
  #          paste0(shp.filepath,"training_areas/",split.name,".gpkg"),
  #          split.name)
  #writeOGR(hex.data,
  #          dsn=paste0(sdm.path,"hex/",name,"_hex-grid.gpkg"),
  #          layer=name,
  #          driver="SQLite")

  if(i==1){
    PAM=as.data.frame(hex.vals)
    colnames(PAM)[i]=name
  }else{
    PAM=cbind(PAM,hex.vals)
    index=which(colnames(PAM)=="hex.vals")
    colnames(PAM)[index]=name
  }
}

print("Done")
```

```
print(errors)

row.names(PAM)=paste0('ID',1:nrow(PAM))
```

```
PAM2=t(PAM)

write.csv(PAM,paste0(sdm.path,"eco_null_PAM.csv"),row.names = T,quote=F)
```

```
 [1] "Errors"                   "Amazona-ochrocephala"
 [3] "Atalotriccus-pilaris"     "Cacicus-cela"
 [5] "Campephilus-melanoleucos" "Cantorchilus-leucotis"
 [7] "Chaetura-brachyura"       "Chaetura-spinicaudus"
 [9] "Chalybura-buffonii"       "Crotophaga-major"
[11] "Dendroplex-picus"         "Euphonia-fulvicrissa"
[13] "Glaucis-hirsutus"         "Icterus-chrysater"
[15] "Juliamyia-julie"          "Manacus-vitellinus"
[17] "Momotus-subrufescens"     "Myiopagis-gaimardii"
[19] "Myrmeciza-longipes"       "Notharchus-pectoralis"
[21] "Oncostoma-olivaceum"      "Pachysylvia-aurantiifrons"
[23] "Pitangus-lictor"          "Ramphocelus-dimidiatus"
[25] "Sicalis-flaveola"         "Trogon-chionurus"
[27] "Trogon-melanurus"
```

3.4 Full unclipped winter models

```
sdm.path="~/Dropbox/motmots/SDMs/MadreSelva_december/"

sdm.list=list.files(paste0(sdm.path,"output_all/75"),pattern="*.tif")
```

```
PAM=NULL

errors="Errors"

for(i in 411:length(sdm.list)){
  name=strsplit(sdm.list[i],"_")[[1]][1]

  if(length(which(shp.list%flike%name))<1){
    errors=c(errors,name)
    next
  }
  if(length(which(sdm.list%flike%name))<1){
    errors=c(errors,name)
    next
  }

  #x=raster(paste0(sdm.path,"output_all/75/",sdm.list[i]))
  #y=readOGR(paste0(shp.path,shp.list[which(shp.list%like%name)]))

  #cr1=crop(x,y)
  #cr2=raster::mask(cr1,y)
  #crs(cr2)=crs(x.temp)
  #cr3=projectRaster(cr2,x.temp,method='ngb',
  #                  res=(res(x)),crs=crs(x))

  # make everything zero so land cells become 0 when stacked
  #cr3[is.na(cr3)]=0

  #x.stack=stack(cr3,x.temp)
```

```
#cr4=calc(x.stack,fun=sum)

#writeRaster(cr4,
#            filename=paste0(sdm.path,'clipped_SDM/',
#                            name,"_cropped.tif"))

# project to hex
# project to hex
#rm(x)
#rm(y)
#rm(cr1)
#rm(cr2)
#rm(cr3)
#rm(cr4)

v.cr=velox(paste0(sdm.path,'output_all/75/',
                            sdm.list[i]))

hex.ext=v.cr$extract(hex)

#hex.ext=raster::extract(cr4,hex)

hex.vals=NULL

for(k in 1:length(hex.ext)){
  n=length(na.omit(hex.ext[[k]]))
  j=sum(na.omit(hex.ext[[k]]))
  if(j==0){
    hex.vals[k]=0
    next
  }
  if(n<11){
    if(j>0.7){hex.vals[k]=1}else{hex.vals[k]=0}
    next
  }
  if(n<41){
    if(j>0.5){hex.vals[k]=1}else{hex.vals[k]=0}
    next
  }
  if(n>40){
    if(j>0.3){hex.vals[k]=1}else{hex.vals[k]=0}
    next
  }
}

hex.data=as.data.frame(hex.vals)
row.names(hex.data)=paste0("ID",1:length(hex))

hex.data=SpatialPolygonsDataFrame(hex,hex.data)

hex.write=st_as_sf(hex.data)

st_write(hex.write,
```

```
          paste0(sdm.path,"noclip/",name,".gpkg"))

  #st_write(hex.data,
  #         paste0(shp.filepath,"training_areas/",split.name,".gpkg"),
  #         split.name)
  #writeOGR(hex.data,
  #         dsn=paste0(sdm.path,"hex/",name,"_hex-grid.gpkg"),
  #         layer=name,
  #         driver="SQLite")

  if(i==1){
    PAM=as.data.frame(hex.vals)
    colnames(PAM)[i]=name
  }else{
    PAM=cbind(PAM,hex.vals)
    index=which(colnames(PAM)=="hex.vals")
    colnames(PAM)[index]=name
  }
}

print("Done")
```

```
print(errors)

row.names(PAM)=paste0('ID',1:nrow(PAM))
PAM2=t(PAM)

write.csv(PAM,paste0(sdm.path,"eco_null_PAM.csv"),row.names = T,quote=F)
```

```
 [1] "Errors"                        "Amazona-ochrocephala"
 [3] "Anthracothorax-nigricollis" "Ardea-cocoi"
 [5] "Atalotriccus-pilaris"       "Cacicus-cela"
 [7] "Campephilus-melanoleucos"   "Cantorchilus-leucotis"
 [9] "Cercomacra-nigricans"       "Chaetura-brachyura"
[11] "Chaetura-spinicaudus"       "Chalybura-buffonii"
[13] "Cotinga-nattererii"         "Crotophaga-major"
[15] "Dendroplex-picus"           "Euphonia-fulvicrissa"
[17] "Glaucis-hirsutus"           "Icterus-chrysater"
[19] "Juliamyia-julie"            "Lophornis-delattrei"
[21] "Manacus-vitellinus"         "Momotus-subrufescens"
[23] "Myiopagis-gaimardii"        "Myrmeciza-longipes"
[25] "Notharchus-pectoralis"      "Oncostoma-olivaceum"
[27] "Pachysylvia-aurantiifrons"  "Pitangus-lictor"
[29] "Ramphocelus-dimidiatus"     "Rhynchocyclus-olivaceus"
[31] "Sicalis-flaveola"           "Trogon-chionurus"
[33] "Trogon-melanurus"
```

4 *Ecostructure* analyses: all species

This section will create *ecostructure* community analyses for models trained to biogegraphic areas and for those based on models created irrespective of biogeography (i.e., neutral).

Prepare the custom loop function for analyses:

```
ecomapper=function(x,k,tol=NULL,n=NULL){
  if(is.null(tol)==T){tol=0.1}
  if(is.null(n)==T){n=10}

  fit=ecos_fit(x,K=k,tol=tol,num_trials=n)

  ord.x=1:nrow(fit$omega)

  palette.x=c('#a6cee3','#1f78b4',
              '#b2df8a','#33a02c',
              '#fb9a99','#e5e5e5',
              '#e31a1c','#fdbf6f',
              '#ff7f00','#cab2d6',
              '#6a3d9a','#ffff99',
              '#b15928','#000000')

  # too many points for blocks
  #

  order_metadata = ord.x,

  features=CountClust::ExtractTopFeatures(fit$theta,
                                          top_features = 5,
                                          method="poisson",
                                          options="max")

  t(apply(features$indices,c(1,2),
        function(x){return(rownames(fit$theta)[x])}))

  # the following step isn't working here
  # out=ecos_nullmodel(x,K=k,null.model = "richness",
  #                      iter_randomized = n,option="BF")

  if(is.na(coords.x)==F){
    #ymin=min(coords.x$Latitude)+0.5
    #ymax=max(coords.x$Latitude)+0.5
    #xmin=min(coords.x$Longitude)+0.5
    #xmax=max(coords.x$Longitude)+0.5
    ecos_plot_pie(omega=fit$omega,
                  lat_lim=c(-8,12),
                  long_lim=c(-86,-82),
                  coords=coords.x,
                  path=paste0(sdm.path, #operates within chunks
                              "costa_rica",
                              "-",k,
                              '-geostructure_plot.png'),
                  color = palette.x,
                  radius=0.05,
                  bgmap_path = map.path) # map path previously defined
    }
}
```

Get metadata.

```
meta.x=read_csv(paste0(sdm.path,"locality_metadata.csv"))
```

```
coords.x=meta.x%>%
  dplyr::select(Longitude,Latitude)%>%
  as.data.frame()

row.names(coords.x)=meta.x$Locality
```

4.1 Example run of models

This example is based on a winter PAM.

```
winter.pam=as.data.frame(read_csv(paste0(sdm.path,"eco_PAM.csv")))
```

```
colnames(winter.pam)[1]="Locality"

winter.pam[is.na(winter.pam)]=0

row.names(winter.pam)=winter.pam$Locality
winter.pam=winter.pam%>%
  dplyr::select(-Locality)
```

Example usage for one level of K

```
ecomapper(x=winter.pam,k=2)
```

5 Creating clusters

The goal of this pipeline is to group species together based on their geographic similarity. Species overlap to varying degrees, allowing us to cluster species together based on their distributional similarities. We will subsequently be abe to look at the distributions of these clusters and the species that comprise them. Niche models are derived from Velde 20201, clustering code from Cooper 2021.

Note that this is an example of the code used; not all output is shown here.

5.1 Example usage of cluster pipeline

December PAM

```
x=as.data.frame(read_csv(paste0(filepath,"SDMs/MadreSelva_december/eco_PAM.csv")))
```

```
## New names:
## Rows: 372 Columns: 684
## -- Column specification
## ---------------------------------------------------------- Delimiter: "," chr
## (1): ...1 dbl (683): Acanthidops-bairdi, Accipiter-bicolor, Accipiter-cooperii,
## Accipi...
## i Use `spec()` to retrieve the full column specification for this data. i
## Specify the column types or set `show_col_types = FALSE` to quiet this message.
## * `` -> `...1`
```

```
subset=read_csv(paste0(filepath,"ecostructure/studytaxa_migrants.csv"))
```

```
## Rows: 721 Columns: 4
## -- Column specification --------------------------------------------------------
```

```
## Delimiter: ","
## chr (4): FAMILY, SCINAME, Migrant, Exclude
##
## i Use `spec()` to retrieve the full column specification for this data.
## i Specify the column types or set `show_col_types = FALSE` to quiet this message.
```

```
subset=subset%>%filter(Exclude=="Include")%>%
  select(-FAMILY,-Exclude)

subnames=gsub(" ","-",subset$SCINAME)

pam.names=colnames(x)

winter.x=x[,which(pam.names%in%subnames)]

rm(x)
rm(subset)
```

```
summary(winter.x[,1:5])
```

```
##  Agelaius-phoeniceus Amblycercus-holosericeus Basileuterus-culicivorus
##  Min.   :0.0000      Min.   :0.0000           Min.   :0.0000
##  1st Qu.:1.0000      1st Qu.:1.0000           1st Qu.:1.0000
##  Median :1.0000      Median :1.0000           Median :1.0000
##  Mean   :0.7796      Mean   :0.9704           Mean   :0.9086
##  3rd Qu.:1.0000      3rd Qu.:1.0000           3rd Qu.:1.0000
##  Max.   :1.0000      Max.   :1.0000           Max.   :1.0000
##  Basileuterus-melanogenys Basileuterus-melanotis
##  Min.   :0.0000           Min.   :0.0000
##  1st Qu.:0.0000           1st Qu.:1.0000
##  Median :0.0000           Median :1.0000
##  Mean   :0.1478           Mean   :0.8118
##  3rd Qu.:0.0000           3rd Qu.:1.0000
##  Max.   :1.0000           Max.   :1.0000
```

```
winter.x[is.na(winter.x)]=0
richness=rowSums(winter.x)

hist(richness/ncol(winter.x))
```

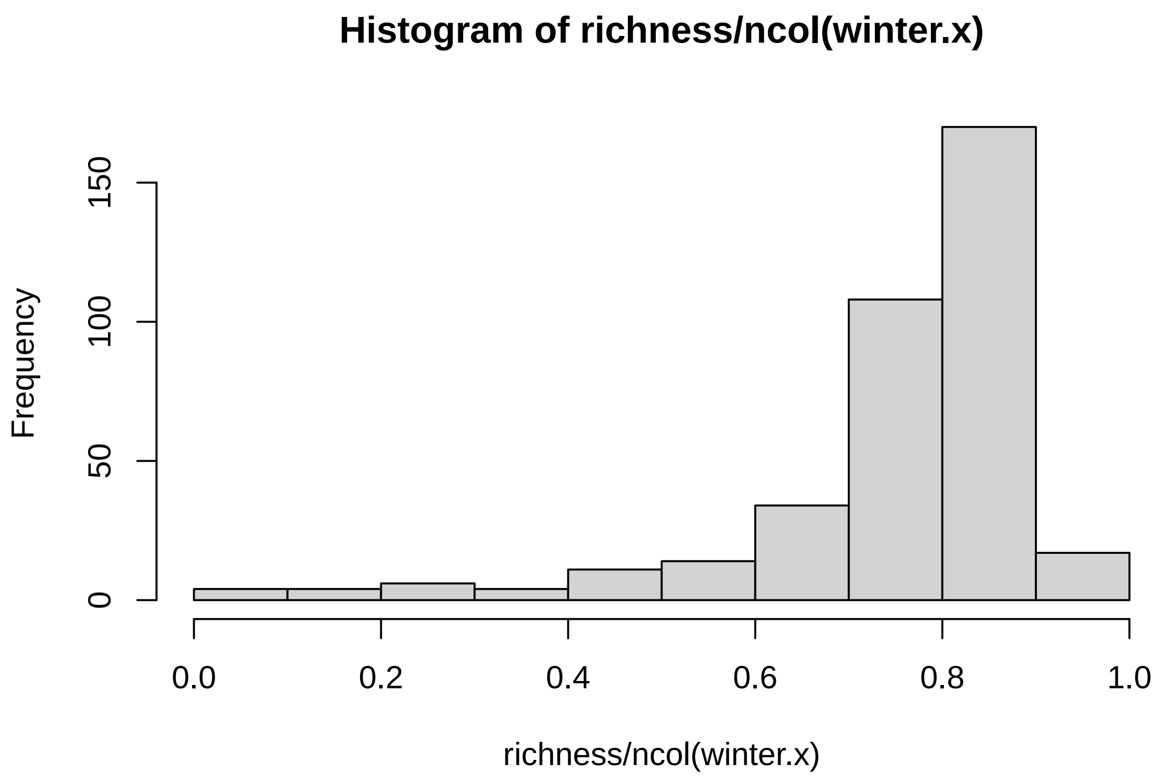


```
colnames(winter.x[,1:9])
```

```
## [1] "Agelaius-phoeniceus"      "Amblycercus-holosericeus"
## [3] "Basileuterus-culicivorus" "Basileuterus-melanogenys"
## [5] "Basileuterus-melanotis"   "Basileuterus-rufifrons"
## [7] "Cacicus-uropygialis"      "Camptostoma-imberbe"
## [9] "Camptostoma-obsoletum"
```

```
x2=winter.x
```

Clustering Code

Now, to create a code that will process and cluster these data.

```
x=as.data.frame(read_csv(paste0(filepath,"SDMs/MadreSelva_december/eco_PAM.csv")))
```

```
## New names:
## Rows: 372 Columns: 684
## -- Column specification
## -------------------------------------------------------- Delimiter: "," chr
## (1): ...1 dbl (683): Acanthidops-bairdi, Accipiter-bicolor, Accipiter-cooperii,
## Accipi...
## i Use `spec()` to retrieve the full column specification for this data. i
## Specify the column types or set `show_col_types = FALSE` to quiet this message.
## * `` -> `...1`
```

```
subset=read_csv(paste0(filepath,"ecostructure/studytaxa.csv"))
```

```
## Rows: 721 Columns: 4
```

```
## -- Column specification --------------------------------------------------------
## Delimiter: ","
## chr (4): FAMILY, SCINAME, Migrant, Exclude
##
## i Use `spec()` to retrieve the full column specification for this data.
## i Specify the column types or set `show_col_types = FALSE` to quiet this message.
```

```
subset=subset%>%filter(Exclude=="Include")%>%
  select(-FAMILY,-Exclude)
subnames=gsub(" ","-",subset$SCINAME)
pam.names=colnames(x)
winter.x=x[,which(pam.names%in%subnames)]
rm(x)
rm(subset)

winter.x[is.na(winter.x)]=0
richness=rowSums(winter.x)

x2=winter.x
```

```
xdata=x2

ncluster=11

# writepath=paste0(filepath,"SDMs/MadreSelva_december/")

level="M"

# removed ncluster variable
# now determines best group number

clustertaxa=function(ncluster,xdata,writepath,level){

  if(exists('ncluster')==F){ncluster=5}

  x3=xdata# %>%
    #select(-`X1`)

  xnames=colnames(x3)

  #x4=x3[,-c(1:5)]

  #col.x=colnames(x3)
  x4=as.data.frame(unclass(t(x3)))

  #colnames(x4)=xnames

  #for(i in 1:ncol(x4)){
  #  x4[,i]=as.numeric(as.character(x4[,i]))
  #}

  name.vector=unique(xnames)

  #colnames(x4)=name.vector
```

```
  wss=(nrow(x4)-1)*sum(apply(x4,2,var))
  for(v in 2:40){
    wss[v]=sum(kmeans(x4,centers=v)$withinss)
  }

  #plot(1:40,wss,type="b",xlab="Number of Clusters",
  #   ylab="Within groups sum of squares")

  set.seed(123)

  print(fviz_nbclust(x4,kmeans,nstart=2,method="gap_stat",
               nboot=100,k.max=30)+
    labs(subtitle = "Gap Statistic"))

  # ncluster.det=which(wss==min(wss))

  # defined from above plot

  clust.x=hclust(dist(x4),method="average")
  plot(clust.x)
  rect.hclust(clust.x,k=ncluster)

  set.seed(20)

  x.tree=as.phylo(clust.x)

  write.tree(x.tree,file=paste0(writepath,"hclust_",
                              level,"_K",ncluster,".tre"))

  #for(k in 11){
  #  xclust=kmeans(x4,ncluster[k],nstart=20)
  #  return(xclust)
  #}

  xclust=kmeans(x4,ncluster)
  #return(xclust)

  assignments=as.data.frame(xclust$cluster)

  write.csv(assignments,
            paste0(writepath,"clusters_",level,
                   "_K",ncluster,".csv"),
            row.names = T,quote = F)

  #set.seed(1000)
  #fit=ecos_fit(x6,K=2,tol=0.1,num_trials=10)
  #print(fit)
}
```

```
writepath=paste0(filepath,"ecostructure/git_code/")
clustertaxa(xdata=x2,ncluster=13,level="M",writepath=writepath)
```

```
## Warning: did not converge in 10 iterations
```

```
## Warning: did not converge in 10 iterations

## Warning: did not converge in 10 iterations

## Warning: did not converge in 10 iterations
```

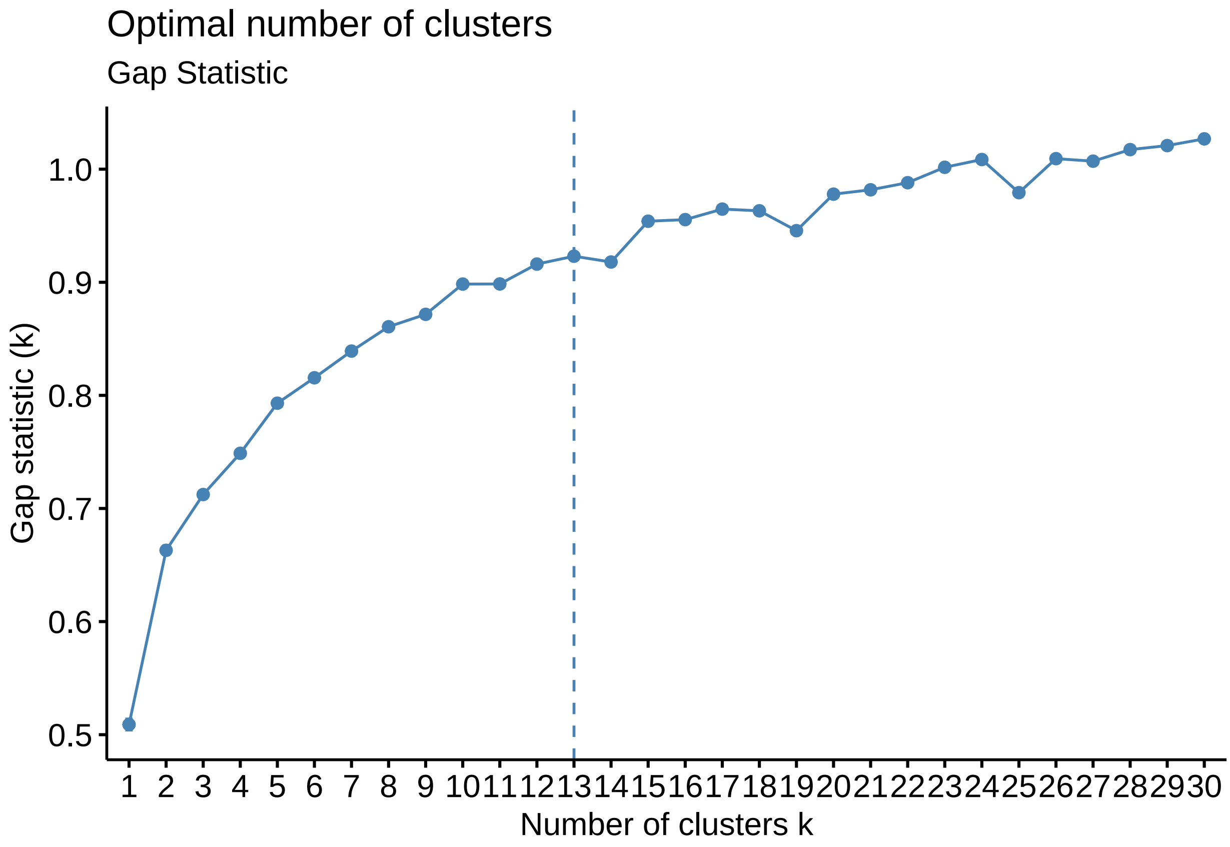

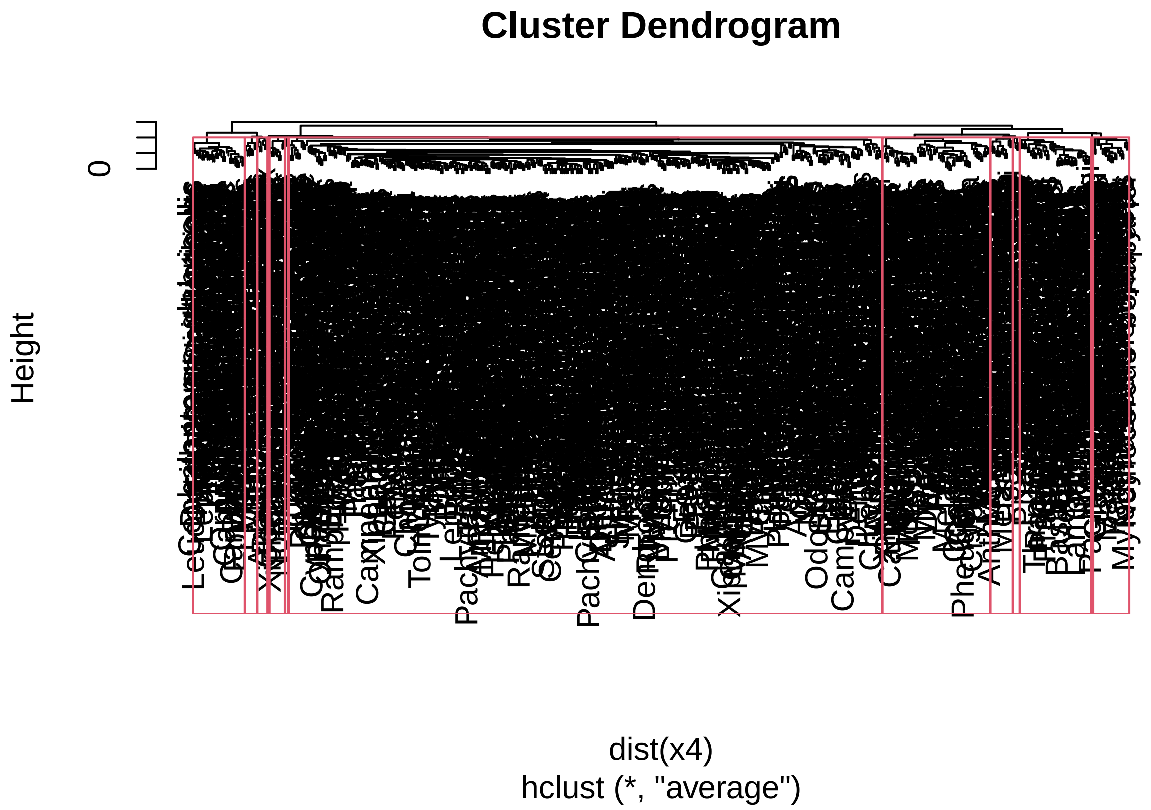


```
# read file from actual analysis
x=as.data.frame(read_csv(paste0(filepath,"SDMs/MadreSelva_december/eco_null_PAM.csv")))
```

```
## New names:
## Rows: 372 Columns: 684
## -- Column specification
## -------------------------------------------------------- Delimiter: "," chr
## (1): ...1 dbl (683): Acanthidops-bairdi, Accipiter-bicolor, Accipiter-cooperii,
## Accipi...
## i Use `spec()` to retrieve the full column specification for this data. i
## Specify the column types or set `show_col_types = FALSE` to quiet this message.
## * `` -> `...1`
```

```
pam.names=colnames(x)

winter.x=x[,which(pam.names%in%subnames)]

rm(x)
rm(subset)
```

```
## Warning in rm(subset): object 'subset' not found
```

```
summary(winter.x[,1:5])
```

```
##  Acanthidops-bairdi Accipiter-bicolor Accipiter-cooperii Accipiter-striatus
##  Min.   :0.0000     Min.   :0.0000    Min.   :0.000      Min.   :0.0000
##  1st Qu.:0.0000     1st Qu.:1.0000    1st Qu.:0.000      1st Qu.:1.0000
##  Median :0.0000     Median :1.0000    Median :1.000      Median :1.0000
```

```
##  Mean   :0.3414     Mean   :0.9301    Mean   :0.586      Mean   :0.9435
##  3rd Qu.:1.0000     3rd Qu.:1.0000    3rd Qu.:1.000      3rd Qu.:1.0000
##  Max.   :1.0000     Max.   :1.0000    Max.   :1.000      Max.   :1.0000
##  Accipiter-superciliosus
##  Min.   :0.0000
##  1st Qu.:0.0000
##  Median :1.0000
##  Mean   :0.7258
##  3rd Qu.:1.0000
##  Max.   :1.0000
```

```
winter.x[is.na(winter.x)]=0
richness=rowSums(winter.x)

hist(richness/ncol(winter.x))
```

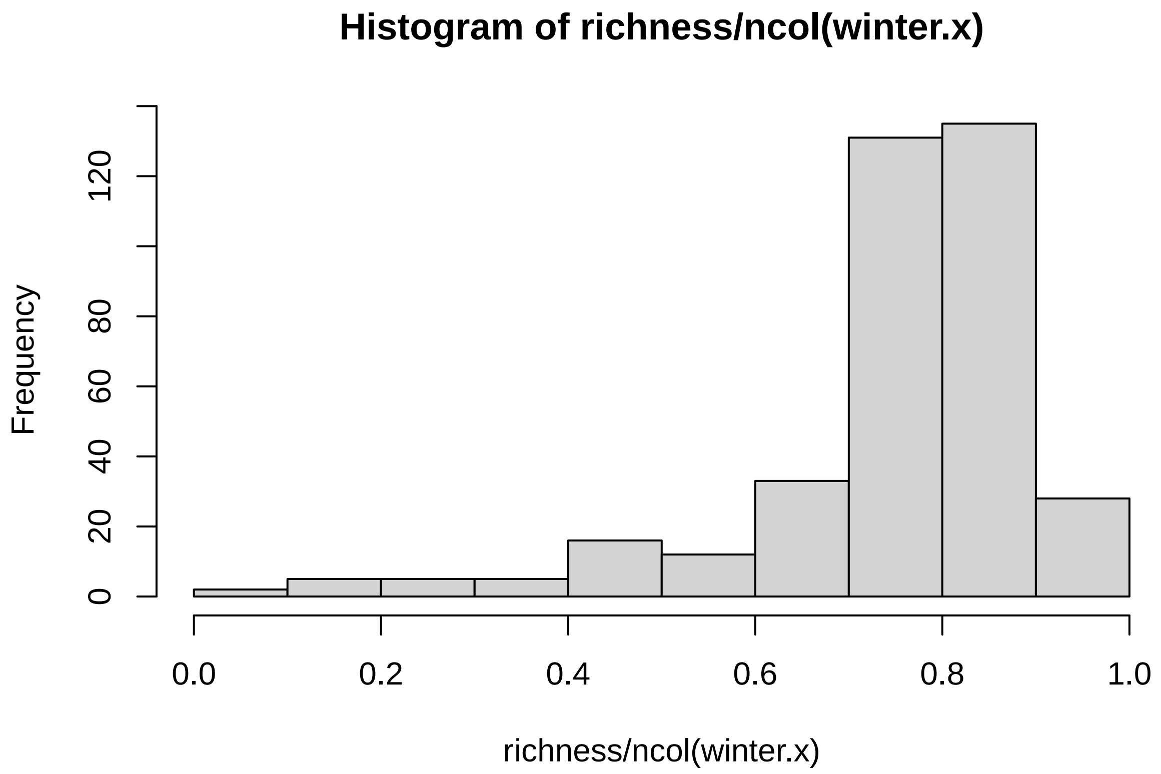


```
colnames(winter.x[,1:9])
```

```
## [1] "Acanthidops-bairdi"         "Accipiter-bicolor"
## [3] "Accipiter-cooperii"         "Accipiter-striatus"
## [5] "Accipiter-superciliosus"    "Agelaius-phoeniceus"
## [7] "Amblycercus-holosericeus"   "Anabacerthia-variegaticeps"
## [9] "Anthracothorax-prevostii"
```

```
x2=winter.x
```

6 Cluster comparisons

Using the output from the clusters of summer and winter data, we can compare cluster assignments. The following examplar is comparing clusters from neutral assignments.

```
filepath="~/Dropbox/motmots/SDMs/"
```

6.1 Loading models, assigning class

```
winter=read_csv(paste0(filepath,"MadreSelva_december/clusters_null_K11.csv"))
```

```
## New names:
## Rows: 538 Columns: 2
## -- Column specification
## -------------------------------------------------------- Delimiter: "," chr
## (1): ...1 dbl (1): xclust$cluster
## i Use `spec()` to retrieve the full column specification for this data. i
## Specify the column types or set `show_col_types = FALSE` to quiet this message.
## * `` -> `...1`
```

```
summer=read_csv(paste0(filepath,"SanJose_june/clusters_null_K18.csv"))
```

```
## New names:
## Rows: 464 Columns: 2
## -- Column specification
## -------------------------------------------------------- Delimiter: "," chr
## (1): ...1 dbl (1): xclust$cluster
## i Use `spec()` to retrieve the full column specification for this data. i
## Specify the column types or set `show_col_types = FALSE` to quiet this message.
## * `` -> `...1`
```

```
colnames(summer)=colnames(winter)=c("Name","Cluster")
```

First, we need to correct for different cluster names and for species that are present in one analysis but not in the other. `outersect` function from this blog post.

```
sum.names=summer$Name
win.names=winter$Name

name.list=Reduce(intersect,list(sum.names,win.names))

outersect <- function(x, y) {
  sort(c(x[!x%in%y],
         y[!y%in%x]))
}

miss.list=Reduce(outersect,list(sum.names,win.names))

sum.red=summer[which(summer$Name%in%name.list),]
win.red=winter[which(winter$Name%in%name.list),]
```

```
# Missing Taxa

print(miss.list)
```

Now, to identify core species that are together.

```
# Cluster x is summer, y is winter
x=inner_join(sum.red,win.red,by='Name')

#Rows, Cols
# summer, winter
table(x$Cluster.x,x$Cluster.y)
```

```
##
##       1  2  3  4  5  6  7  8  9 10 11
##   1   4  0  0 11  1  0  0  0  0  1  2
##   2   0  1  7  0  5  0  0  1 13  0 10
##   3   0  3  2  0  2  0  2 12  0  1  0
##   4   0  0  0  1  0  4  2  1  0  0  0
##   5   0  2 13  0  0  0  1  1  6  0  4
##   6   0  1  3  1  0  0  1 12  1  0  0
##   7   0  0  0  0  2  0 10  1  0  0  0
##   8   0  1  0  3  4  0  9  1  2  0  0
##   9   0  5  6  0 12  0  2  0  2  0  2
##   10  8  1  0  2  1  0  0  0  1  0  4
##   11  1  1  3  6  1  0  0  0  0  2  0
##   12  1  0  1  1  0  0  0  0  2  6  2
##   13  0  0  0  0  1  0 17  2  0  0  0
##   14  0  0  2  0  0 30  0  4  0  2  0
##   15  0 36  8  0  0  0  0  1 24  0  3
##   16  0  2  3  0  0  2  0  1  1  3  1
##   17  0  0 15  0  0  6  0 19  1  0  1
##   18  0  3  0  0  1  0  0  1 16  0 16
```

The above table shows all the combinations of groups, as they occur within the data table and within the dataset. The eigenvector corresponds to groups that have the same numerical assignment between summer and winter. In this instance, `Cluster.x` (summer) group 4 corresponds mostly to `Cluster.y` (winter) group 6. It appears as though groups are split between time periods, and that there is large discordance between these time periods.

We can define clusters as **stable**, **split**, or **dispersed**. We define this as groups that are 66% or more in the same cluster, retain at least 33% in at least two clusters, or lack 33% in more than one group. We define these based on the **summer** clustering, where more groups exist.

```
clust.table=as.matrix(table(x$Cluster.x,x$Cluster.y))

for(i in 1:nrow(clust.table)){
  x.ord=clust.table[i,]
  x.ord=x.ord[order(x.ord,decreasing = T)]
  x.max=x.ord[1]
  x.sec=x.ord[2]
  x.sum=sum(x.ord)

  if(x.max/x.sum>0.66){
    print(paste0("Cluster ",i,": STABLE."))
    next
  }
  if(x.max/x.sum>0.33){
    if(x.sec/x.sum>0.33){
      print(paste0("Cluster ",i,": SPLIT."))
      next
```

```
    }else{
      print(paste0("Cluster ",i,": DIFFUSE."))
      next
    }
  }else{
    print(paste0("Cluster ",i,": DIFFUSE."))
    next
  }
}
```

```
## [1] "Cluster 1: DIFFUSE."
## [1] "Cluster 2: DIFFUSE."
## [1] "Cluster 3: DIFFUSE."
## [1] "Cluster 4: DIFFUSE."
## [1] "Cluster 5: DIFFUSE."
## [1] "Cluster 6: DIFFUSE."
## [1] "Cluster 7: STABLE."
## [1] "Cluster 8: DIFFUSE."
## [1] "Cluster 9: DIFFUSE."
## [1] "Cluster 10: DIFFUSE."
## [1] "Cluster 11: DIFFUSE."
## [1] "Cluster 12: DIFFUSE."
## [1] "Cluster 13: STABLE."
## [1] "Cluster 14: STABLE."
## [1] "Cluster 15: SPLIT."
## [1] "Cluster 16: DIFFUSE."
## [1] "Cluster 17: SPLIT."
## [1] "Cluster 18: SPLIT."
```

So, from this we can assess the following:

The following is an assessment from summer *into* winter.

Stable Clusters: Cluster 7 (as 7); Cluster 13 (as 7); Cluster 14 (as 6)

Split Clusters: Cluster 15, Cluster 17, Cluster 18

Dispersed Clusters: All other clusters (1:6,8:12,16)

Because of this, it is clear that these communities are not very well maintained in time. but we can learn about them individually.

```
# dataframe=x

summer.look=function(dataframe,cluster){
  dataframe$Name=as.character(dataframe$Name)
  sub.dat=dataframe[which(dataframe$Cluster.x==cluster),]
  #print(sub.dat)

  sub.names=sub.dat$Name
  sub.files=paste0(sub.names,"_cropped.tif")

  y=stack(paste0(filepath,"SanJose_june/clipped_SDM/",sub.files))
  #y[is.na(y)]=0
  y2=calc(y,fun=sum)

  y3=as.data.frame(y2,xy=T)
```

```
  a=ggplot()+
    geom_raster(data=y3,
                aes(x=x,y=y,fill=layer))+
    labs(x="Longitude",y="Latitude",fill="Richness",
         title=paste0("Summer: Cluster ",cluster))+
    scale_fill_viridis_c(option="viridis")+
    theme_classic()+
    theme(plot.title=element_text(hjust=0.5))+
    coord_quickmap()

  x=stack(paste0(filepath,
                 "MadreSelva_december/clipped_SDM/",sub.files))
  #x[is.na(x)]=0
  x2=calc(x,fun=sum)

  x3=as.data.frame(x2,xy=T)


  b=ggplot()+
    geom_raster(data=x3,
                aes(x=x,y=y,fill=layer))+
    labs(x="Longitude",y="Latitude",fill="Richness",
         title=paste0("Winter: Cluster ",cluster))+
    scale_fill_viridis_c(option="viridis")+
    theme_classic()+
    theme(plot.title=element_text(hjust=0.5))+
    coord_quickmap()

  grid.arrange(a,b,ncol=2)
}
```

Stable Clusters

```
for(i in 1:length(stable)){
  val=stable[i]
  print(x[x$Cluster.x==val,])
  summer.look(dataframe = x,cluster = val)
}
```

```
## # A tibble: 13 x 3
##    Name                        Cluster.x Cluster.y
##    <chr>                           <dbl>     <dbl>
##  1 Camptostoma-imberbe                 7         7
##  2 Crypturellus-cinnamomeus            7         7
##  3 Icterus-pectoralis                  7         7
##  4 Icterus-pustulatus                  7         7
##  5 Leptotila-plumbeiceps               7         5
##  6 Megascops-cooperi                   7         8
##  7 Ortalis-vetula                      7         7
##  8 Parabuteo-unicinctus                7         7
##  9 Rostrhamus-sociabilis               7         5
## 10 Sublegatus-arenarum                 7         7
## 11 Trogon-elegans                      7         7
```

```
## 12 Vireo-pallens                   7          7
## 13 Xiphorhynchus-flavigaster       7          7
```

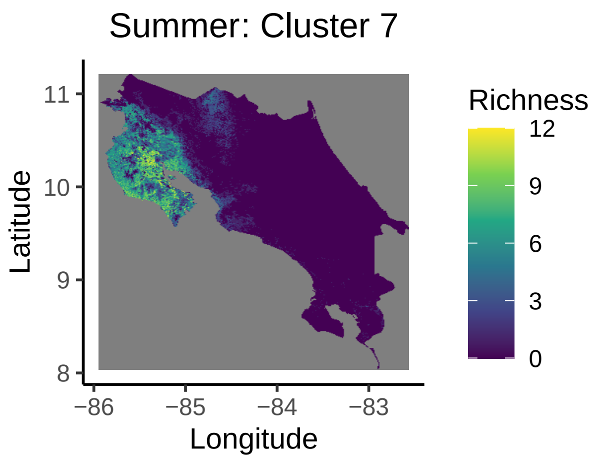

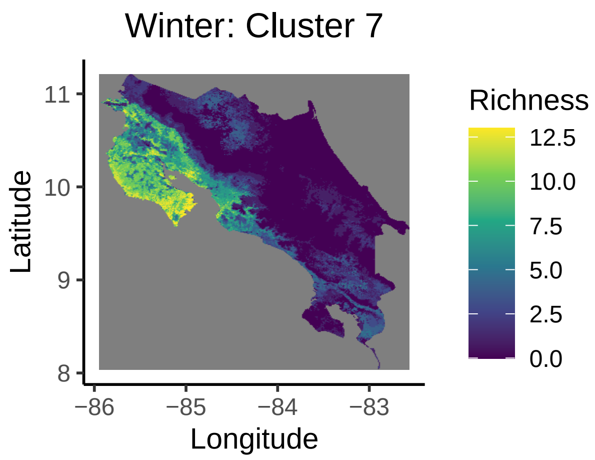


```
## # A tibble: 20 x 3
##    Name                        Cluster.x Cluster.y
##    <chr>                           <dbl>     <dbl>
##  1 Arremonops-rufivirgatus            13         7
##  2 Calocitta-formosa                  13         7
##  3 Campylorhynchus-rufinucha          13         7
##  4 Chiroxiphia-linearis               13         7
##  5 Chlorostilbon-canivetii            13         7
##  6 Colinus-cristatus                  13         8
##  7 Eumomota-superciliosa              13         7
##  8 Euphonia-affinis                   13         7
##  9 Geranospiza-caerulescens           13         5
## 10 Glaucidium-brasilianum             13         7
## 11 Morococcyx-erythropygus            13         7
## 12 Myiarchus-nuttingi                 13         7
## 13 Myiarchus-tyrannulus               13         7
## 14 Pachyramphus-aglaiae               13         8
## 15 Passerina-caerulea                 13         7
## 16 Peucaea-ruficauda                  13         7
## 17 Platyrinchus-cancrominus           13         7
## 18 Polioptila-albiloris               13         7
## 19 Thryophilus-pleurostictus          13         7
## 20 Trogon-melanocephalus              13         7
```

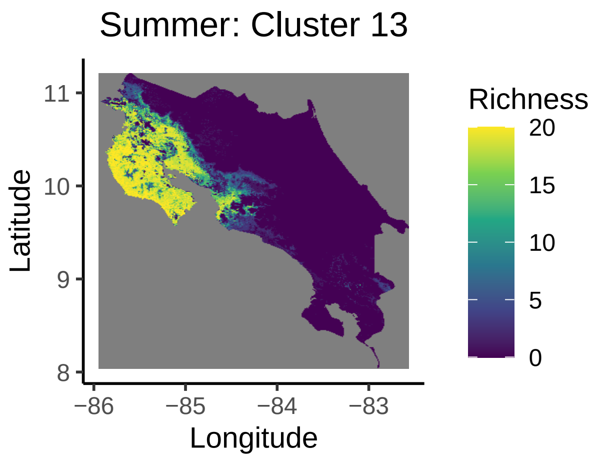

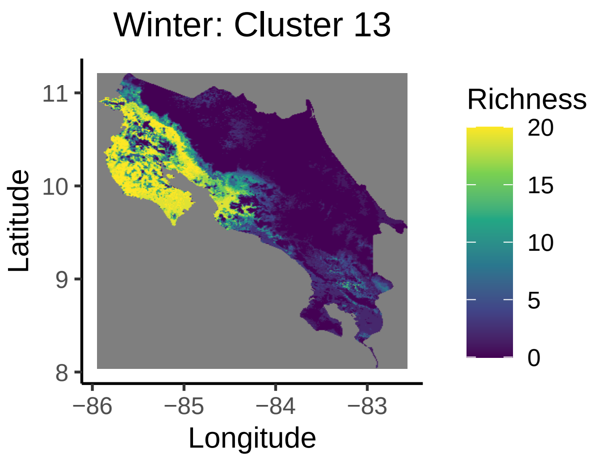


```
## # A tibble: 38 x 3
##    Name                        Cluster.x Cluster.y
##    <chr>                           <dbl>     <dbl>
##  1 Acanthidops-bairdi                 14         6
##  2 Atlapetes-tibialis                 14         6
##  3 Basileuterus-melanogenys           14         6
##  4 Buteo-jamaicensis                  14         3
##  5 Catharus-frantzii                  14         6
##  6 Catharus-gracilirostris            14         6
##  7 Chlorospingus-pileatus             14         6
##  8 Contopus-lugubris                  14         6
##  9 Dendrortyx-leucophrys              14         6
## 10 Diglossa-plumbea                   14         6
## # i 28 more rows
```

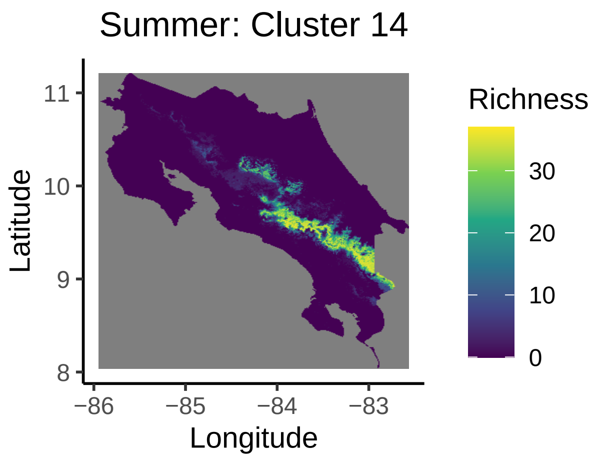

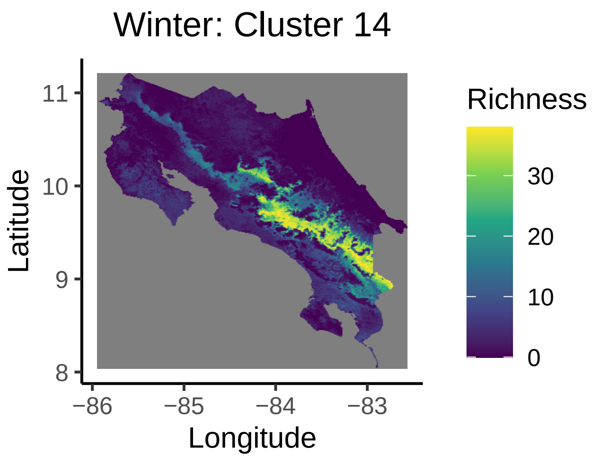

Dispersed Clusters

```
for(i in 1:length(dispersed)){
  val=dispersed[i]
  print(x[x$Cluster.x==val,])
  summer.look(dataframe = x,cluster = val)
}
```

```
## # A tibble: 19 x 3
##    Name                         Cluster.x Cluster.y
##    <chr>                            <dbl>     <dbl>
##  1 Cantorchilus-zeledoni                1        11
##  2 Celeus-castaneus                     1         4
##  3 Chalybura-urochrysia                 1         1
##  4 Cymbilaimus-lineatus                 1         4
##  5 Epinecrophylla-fulviventris          1        11
##  6 Glaucidium-griseiceps                1         4
##  7 Leucopternis-semiplumbeus            1         4
##  8 Monasa-morphoeus                     1         4
##  9 Myiornis-atricapillus                1         4
## 10 Myrmotherula-axillaris               1         1
## 11 Notharchus-tectus                    1         4
## 12 Ornithion-brunneicapillus            1         1
## 13 Querula-purpurata                    1         4
## 14 Saltator-grossus                     1         5
## 15 Tachyphonus-delatrii                 1         1
```

```
## 16 Tangara-inornata                      1         4
## 17 Todirostrum-nigriceps                 1         4
## 18 Tolmomyias-assimilis                  1         4
## 19 Zentrygon-lawrencii                   1        10
```

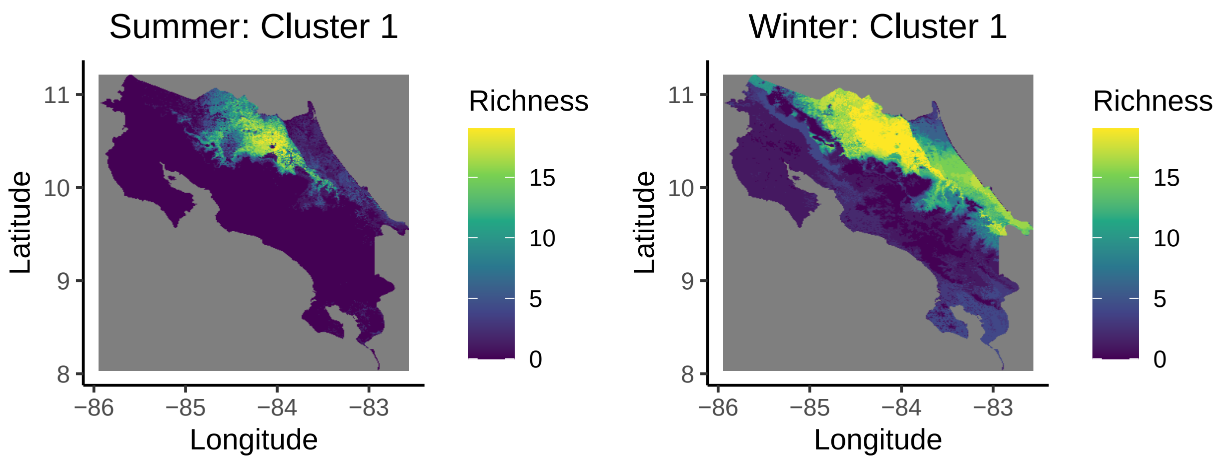


```
## # A tibble: 37 x 3
##    Name                         Cluster.x Cluster.y
##    <chr>                            <dbl>     <dbl>
##  1 Asio-clamator                        2         3
##  2 Ciccaba-nigrolineata                 2         5
##  3 Dacnis-cayana                        2         9
##  4 Elanoides-forficatus                 2         9
##  5 Florisuga-mellivora                  2        11
##  6 Galbula-ruficauda                    2         3
##  7 Gymnopithys-bicolor                  2         9
##  8 Heliomaster-longirostris             2         3
##  9 Henicorhina-leucosticta              2        11
## 10 Klais-guimeti                        2        11
## # i 27 more rows
```

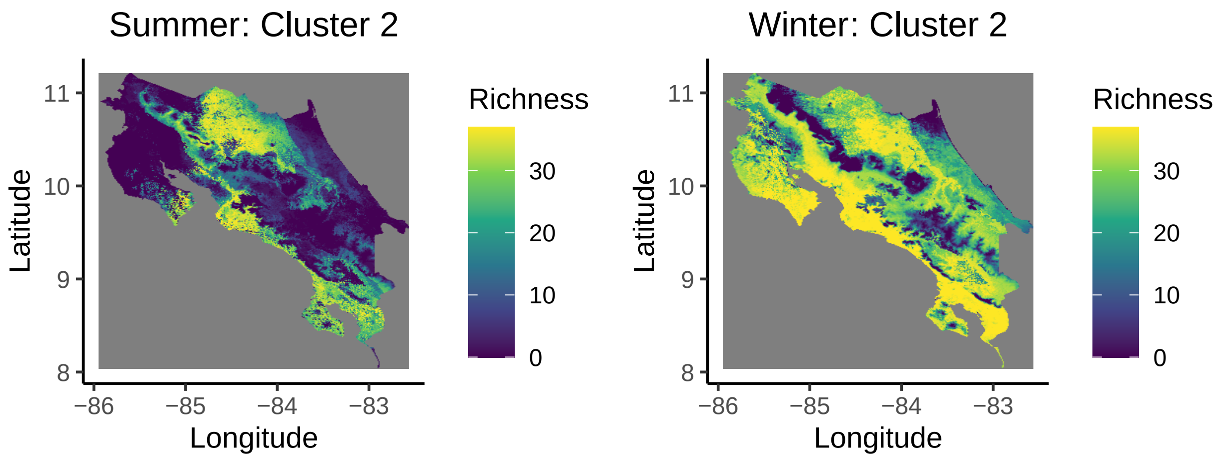


```
## # A tibble: 22 x 3
##    Name                       Cluster.x Cluster.y
##    <chr>                          <dbl>     <dbl>
##  1 Arremon-costaricensis              3         8
##  2 Camptostoma-obsoletum              3         8
##  3 Cantorchilus-elutus                3         2
##  4 Cantorchilus-semibadius            3         7
##  5 Chlorostilbon-assimilis            3         8
##  6 Dendrocincla-anabatina             3         7
##  7 Elaenia-chiriquensis               3         2
##  8 Euphonia-imitans                   3         8
##  9 Euphonia-laniirostris              3         2
## 10 Hylocharis-eliciae                 3         8
## # i 12 more rows
```

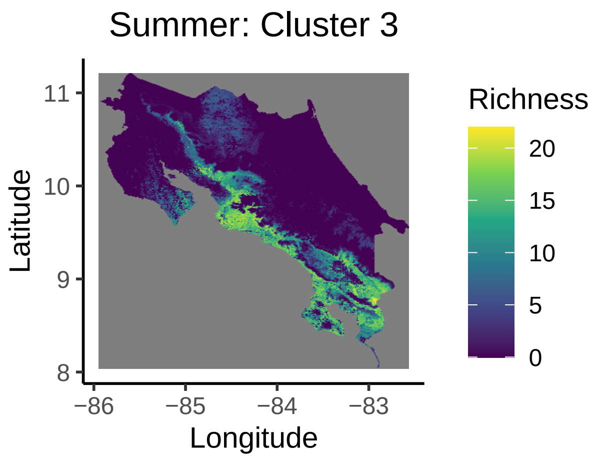

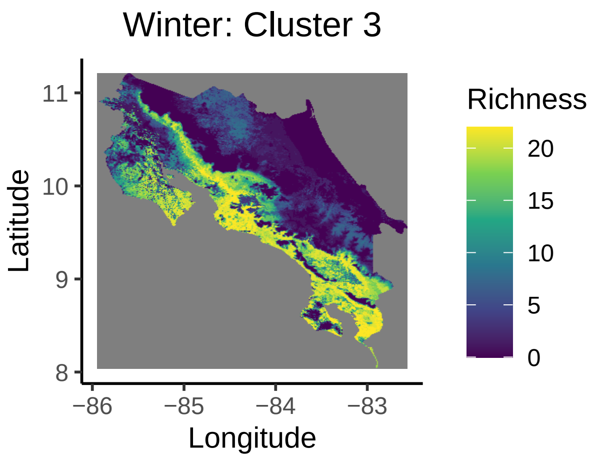


```
## # A tibble: 8 x 3
##   Name                   Cluster.x Cluster.y
##   <chr>                      <dbl>     <dbl>
## 1 Automolus-exsertus             4         8
## 2 Cyanolyca-cucullata            4         6
## 3 Habia-atrimaxillaris           4         7
## 4 Hylomanes-momotula             4         4
## 5 Melozone-cabanisi              4         6
## 6 Melozone-leucotis              4         6
## 7 Phaeomyias-murina              4         7
## 8 Rhodinocichla-rosea            4         6
```

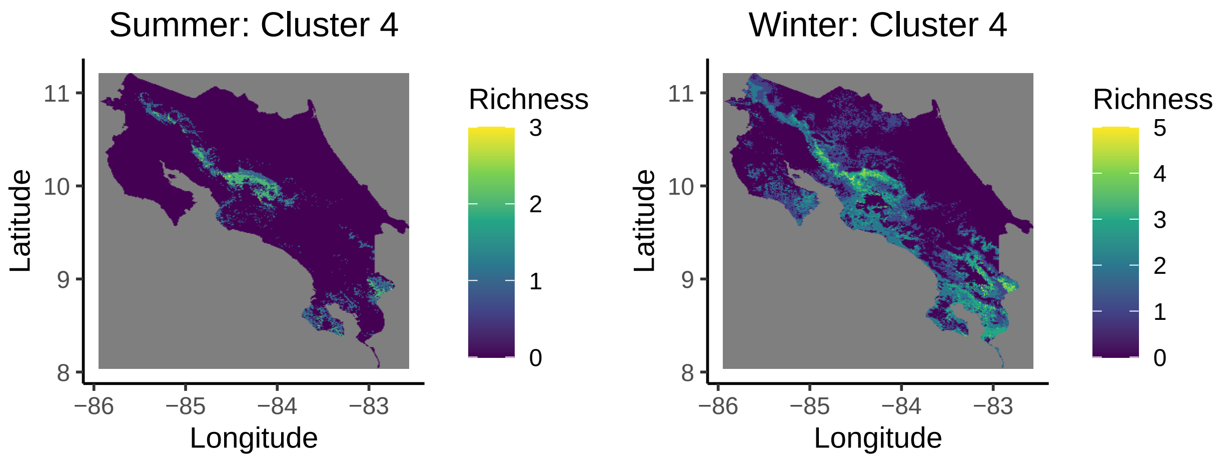


```
## # A tibble: 27 x 3
##    Name                        Cluster.x Cluster.y
##    <chr>                           <dbl>     <dbl>
##  1 Arremon-brunneinucha                5         3
##  2 Aulacorhynchus-prasinus             5         3
##  3 Automolus-subulatus                 5         9
##  4 Campylorhamphus-pusillus            5         3
##  5 Chlorospingus-flavopectus           5         3
##  6 Colaptes-rubiginosus                5         3
##  7 Corapipo-altera                     5        11
##  8 Dacnis-venusta                      5         9
##  9 Dryobates-fumigatus                 5         3
## 10 Euphonia-minuta                     5         9
## # i 17 more rows
```

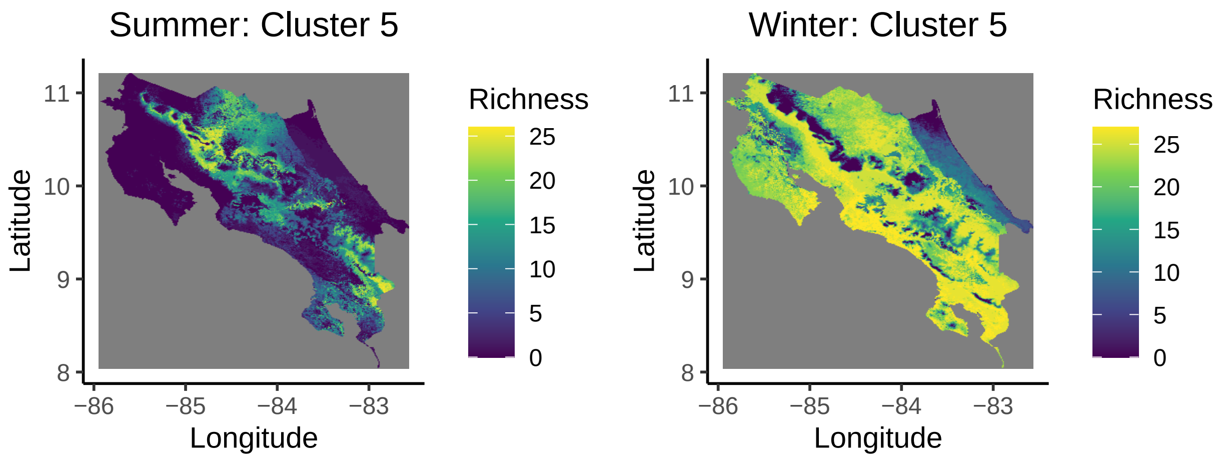


```
## # A tibble: 19 x 3
##    Name                        Cluster.x Cluster.y
##    <chr>                           <dbl>     <dbl>
##  1 Basileuterus-rufifrons              6         8
##  2 Cantorchilus-modestus               6         8
##  3 Columbina-inca                      6         7
##  4 Cyclarhis-gujanensis                6         8
##  5 Cypseloides-niger                   6         9
##  6 Dendrocincla-homochroa              6         8
##  7 Euphonia-hirundinacea               6         3
##  8 Habia-rubica                        6         8
##  9 Heliomaster-constantii              6         8
## 10 Leptotrygon-veraguensis             6         4
## 11 Melanerpes-hoffmannii               6         8
## 12 Pheugopedius-rutilus                6         8
## 13 Philodice-bryantae                  6         8
## 14 Phyllomyias-burmeisteri             6         3
## 15 Pygochelidon-cyanoleuca             6         3
## 16 Thryophilus-rufalbus                6         8
## 17 Vireo-flavoviridis                  6         2
## 18 Zenaida-asiatica                    6         8
## 19 Zenaida-macroura                    6         8
```

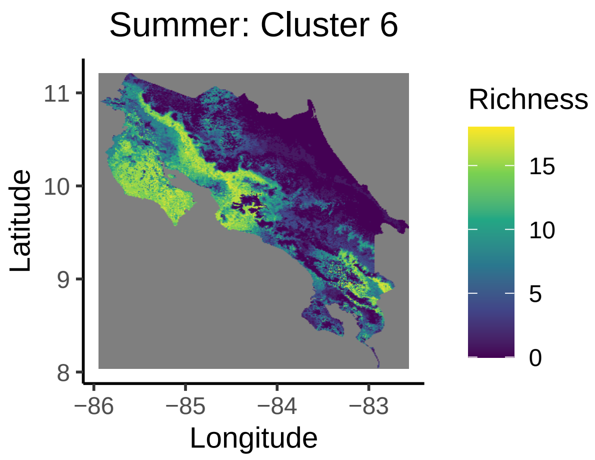

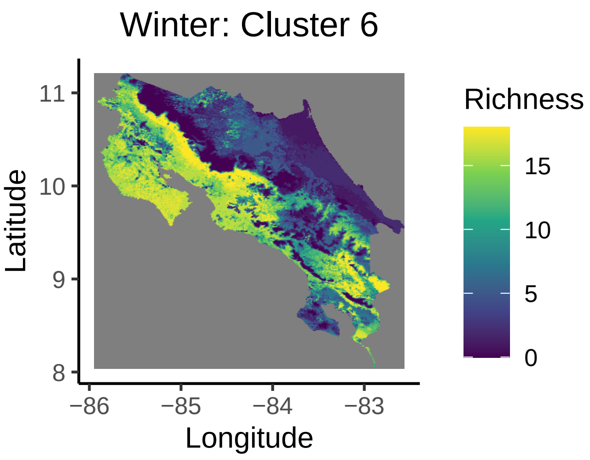


```
## # A tibble: 20 x 3
##    Name                        Cluster.x Cluster.y
##    <chr>                           <dbl>     <dbl>
##  1 Buteo-nitidus                       8         4
##  2 Cathartes-burrovianus               8         7
##  3 Ceratopipra-mentalis                8         5
##  4 Chaetura-fumosa                     8         7
##  5 Cotinga-ridgwayi                    8         7
##  6 Deconychura-longicauda              8         9
##  7 Hylopezus-perspicillatus            8         5
##  8 Hylophilus-flavipes                 8         7
##  9 Lanio-leucothorax                   8         5
## 10 Melanerpes-chrysauchen              8         7
## 11 Myiozetetes-cayanensis              8         4
## 12 Ornithion-semiflavum                8         8
## 13 Panyptila-cayennensis               8         2
## 14 Piculus-simplex                     8         9
## 15 Sclerurus-guatemalensis             8         5
## 16 Sporophila-minuta                   8         4
## 17 Thamnophilus-bridgesi               8         7
## 18 Trogon-bairdii                      8         7
## 19 Tyrannulus-elatus                   8         7
## 20 Tyrannus-savana                     8         7
```

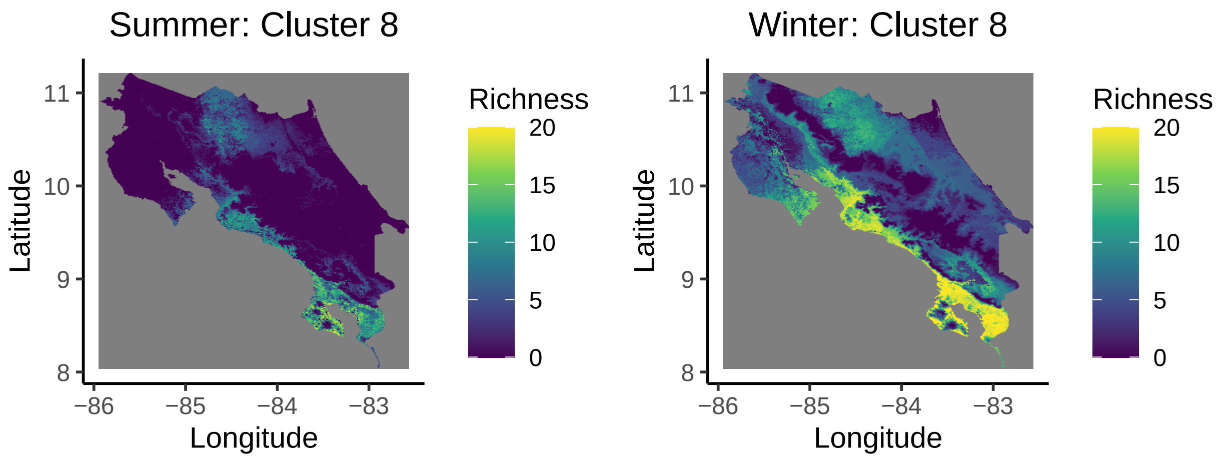


```
## # A tibble: 29 x 3
##    Name                        Cluster.x Cluster.y
##    <chr>                           <dbl>     <dbl>
##  1 Agelaius-phoeniceus                 9         5
##  2 Buteo-albonotatus                   9         3
##  3 Buteo-plagiatus                     9         2
##  4 Buteogallus-anthracinus             9         5
##  5 Campephilus-guatemalensis           9         9
##  6 Caracara-cheriway                   9         3
##  7 Claravis-pretiosa                   9         5
##  8 Columbina-minuta                    9         7
##  9 Columbina-passerina                 9         7
## 10 Crax-rubra                          9         9
## # i 19 more rows
```

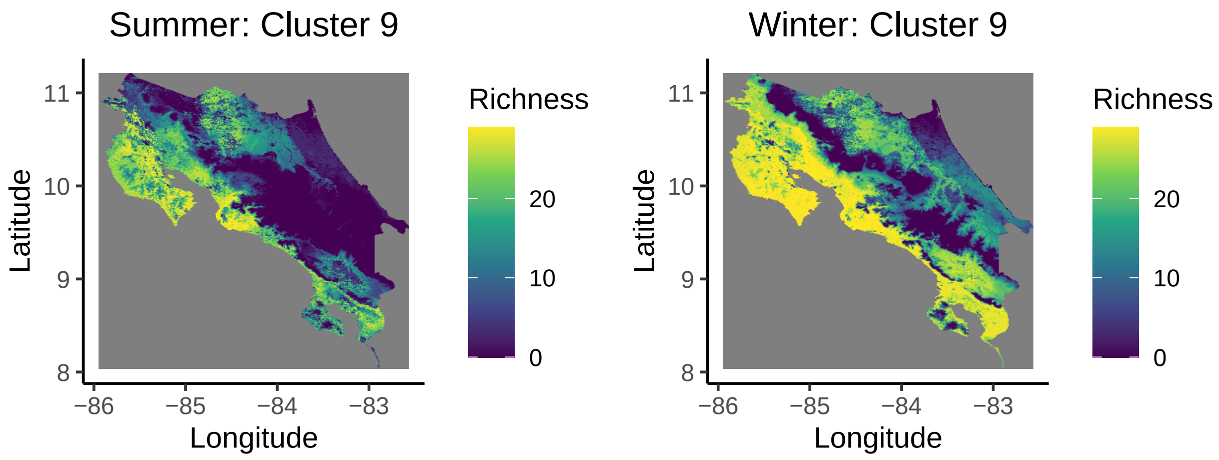


```
## # A tibble: 17 x 3
##    Name                          Cluster.x Cluster.y
##    <chr>                             <dbl>     <dbl>
##  1 Baryphthengus-martii                 10         1
##  2 Cacicus-uropygialis                  10         5
##  3 Cantorchilus-nigricapillus           10        11
##  4 Cantorchilus-thoracicus              10         1
##  5 Carpodectes-nitidus                  10         4
##  6 Celeus-loricatus                     10         1
##  7 Colonia-colonus                      10        11
##  8 Conopias-albovittatus                10         2
##  9 Electron-platyrhynchum               10         1
## 10 Euphonia-gouldi                      10        11
## 11 Glaucis-aeneus                       10         9
## 12 Habia-fuscicauda                     10         1
## 13 Manacus-candei                       10         1
## 14 Melanerpes-pucherani                 10        11
## 15 Nyctibius-grandis                    10         4
## 16 Pheugopedius-atrogularis             10         1
## 17 Thamnophilus-atrinucha               10         1
```

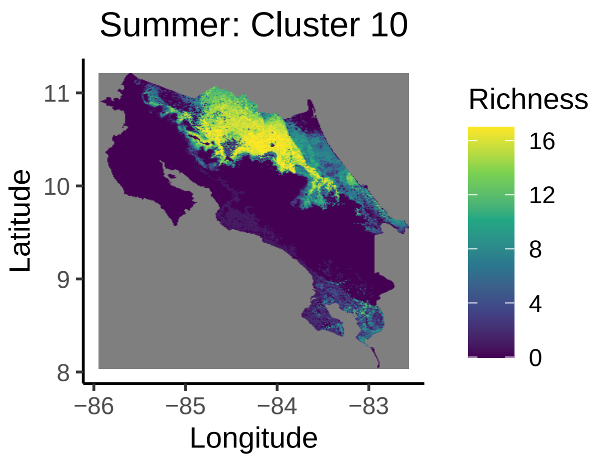

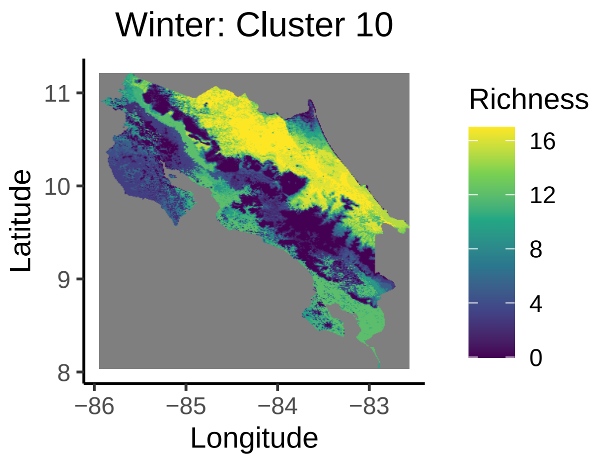


```
## # A tibble: 14 x 3
##    Name                        Cluster.x Cluster.y
##    <chr>                           <dbl>     <dbl>
##  1 Bangsia-arcaei                     11        10
##  2 Chlorothraupis-carmioli            11        10
##  3 Cyphorhinus-phaeocephalus          11         5
##  4 Dysithamnus-mentalis               11         3
##  5 Dysithamnus-striaticeps            11         4
##  6 Electron-carinatum                 11         4
##  7 Hylopezus-dives                    11         4
##  8 Hylophylax-naevioides              11         2
##  9 Lophornis-helenae                  11         4
## 10 Microcerculus-philomela            11         4
## 11 Phaenostictus-mcleannani           11         3
## 12 Piranga-flava                      11         3
## 13 Sipia-laemosticta                  11         4
## 14 Tangara-lavinia                    11         1
```

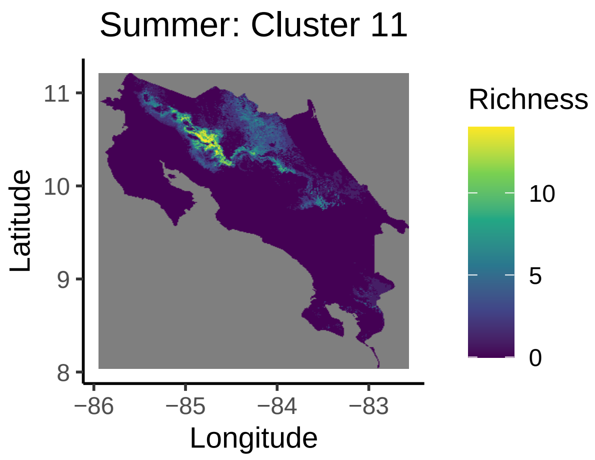

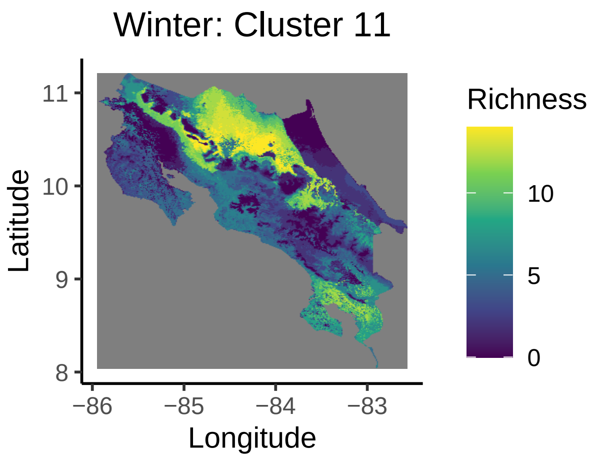


```
## # A tibble: 13 x 3
##    Name                         Cluster.x Cluster.y
##    <chr>                            <dbl>     <dbl>
##  1 Automolus-ochrolaemus               12        10
##  2 Campylorhynchus-zonatus             12        10
##  3 Caryothraustes-poliogaster          12        11
##  4 Chaetura-cinereiventris             12         9
##  5 Chrysothlypis-chrysomelas           12        10
##  6 Dendrocincla-fuliginosa             12        11
##  7 Euphonia-anneae                     12        10
##  8 Microchera-albocoronata             12         3
##  9 Mitrospingus-cassinii               12         4
## 10 Ramphocelus-sanguinolentus          12         1
## 11 Saltator-atriceps                   12         9
## 12 Tangara-florida                     12        10
## 13 Turdus-obsoletus                    12        10
```

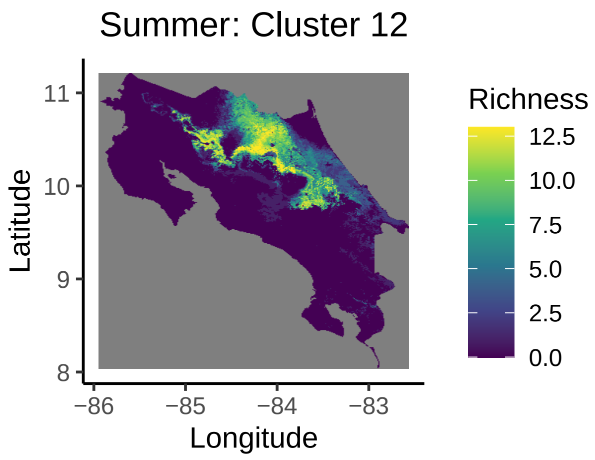

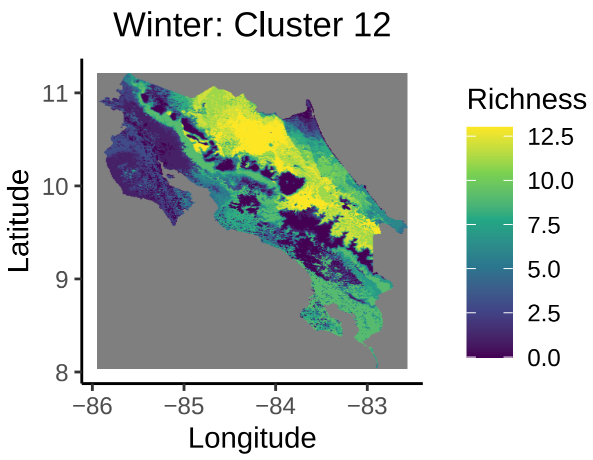


```
## # A tibble: 13 x 3
##    Name                         Cluster.x Cluster.y
##    <chr>                            <dbl>     <dbl>
##  1 Accipiter-bicolor                   16         2
##  2 Anabacerthia-variegaticeps          16        10
##  3 Arremon-crassirostris               16         8
##  4 Catharus-mexicanus                  16         6
##  5 Colibri-delphinae                   16        10
##  6 Discosura-conversii                 16         3
##  7 Elvira-cupreiceps                   16         6
##  8 Eupherusa-nigriventris              16        11
##  9 Hafferia-zeledoni                   16        10
## 10 Lampornis-hemileucus                16         9
## 11 Leptopogon-superciliaris            16         3
## 12 Mitrephanes-phaeocercus             16         3
## 13 Procnias-tricarunculatus            16         2
```

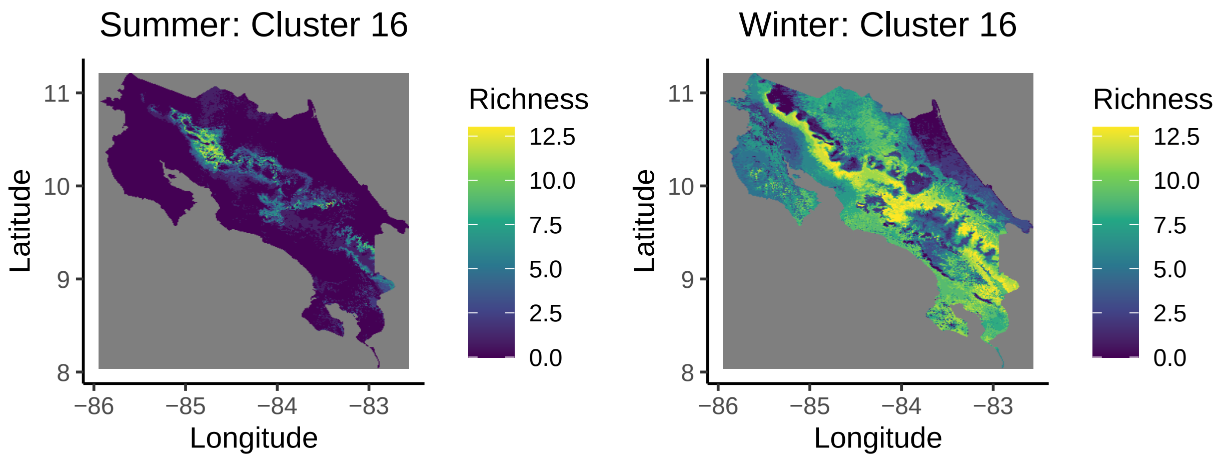

Split Clusters

```
for(i in 1:length(split)){
  val=split[i]
  print(x[x$Cluster.x==val,])
  summer.look(dataframe = x,cluster = val)
}
```

```
## # A tibble: 72 x 3
##    Name                      Cluster.x Cluster.y
##    <chr>                         <dbl>     <dbl>
##  1 Amblycercus-holosericeus         15         9
##  2 Buteo-brachyurus                 15         2
##  3 Buteogallus-urubitinga           15         9
##  4 Capsiempis-flaveola              15        11
##  5 Cathartes-aura                   15         2
##  6 Chaetura-vauxi                   15         9
##  7 Ciccaba-virgata                  15         2
##  8 Coereba-flaveola                 15         9
##  9 Columbina-talpacoti              15         2
## 10 Contopus-cinereus                15         9
## # i 62 more rows
```

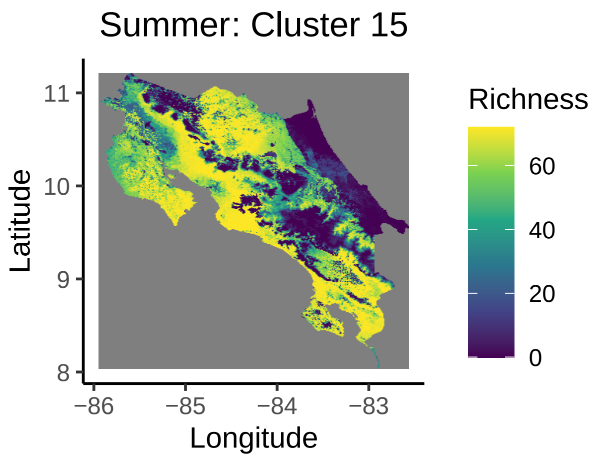

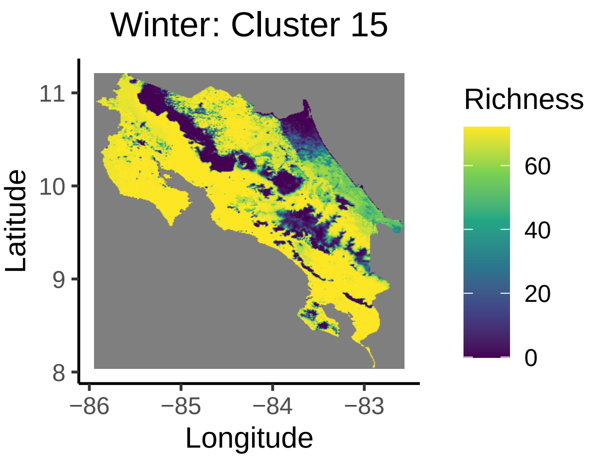


```
## # A tibble: 42 x 3
##    Name                         Cluster.x Cluster.y
##    <chr>                            <dbl>     <dbl>
##  1 Atlapetes-albinucha                 17         8
##  2 Basileuterus-culicivorus            17         3
##  3 Basileuterus-melanotis              17         8
##  4 Campylopterus-hemileucurus          17         8
##  5 Catharus-aurantiirostris            17         8
##  6 Catharus-fuscater                   17         8
##  7 Chamaepetes-unicolor                17         3
##  8 Chlorophonia-callophrys             17         8
##  9 Colibri-cyanotus                    17         8
## 10 Cranioleuca-erythrops               17         3
## # i 32 more rows
```

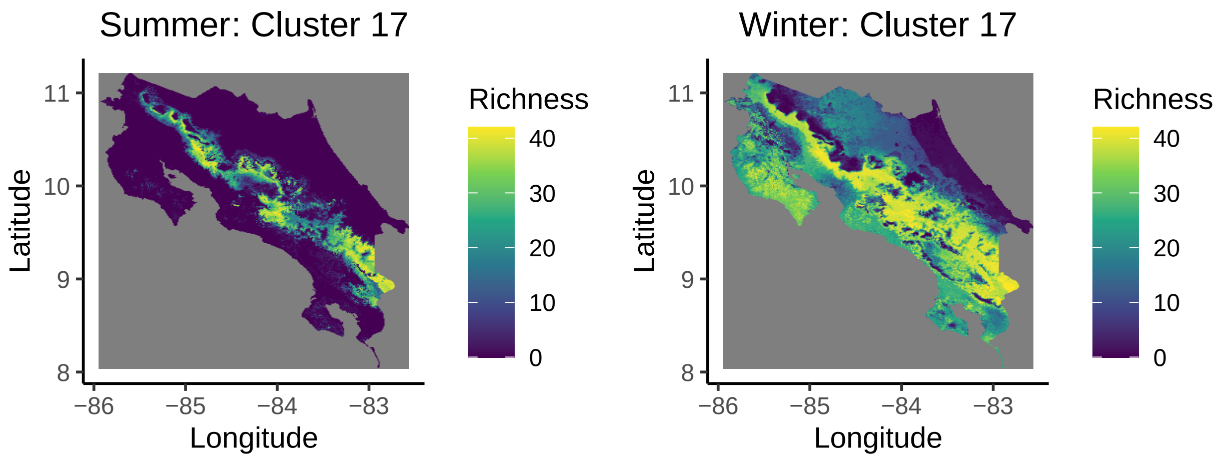


```
## # A tibble: 37 x 3
##    Name                         Cluster.x Cluster.y
##    <chr>                            <dbl>     <dbl>
##  1 Anthracothorax-prevostii            18         8
##  2 Arremon-aurantiirostris             18         9
##  3 Arremonops-conirostris              18         9
##  4 Cercomacroides-tyrannina            18         9
##  5 Chondrohierax-uncinatus             18         2
##  6 Chordeiles-acutipennis              18         5
##  7 Cyanerpes-lucidus                   18        11
##  8 Cyanoloxia-cyanoides                18        11
##  9 Dendrocolaptes-sanctithomae         18        11
## 10 Harpagus-bidentatus                 18        11
## # i 27 more rows
```

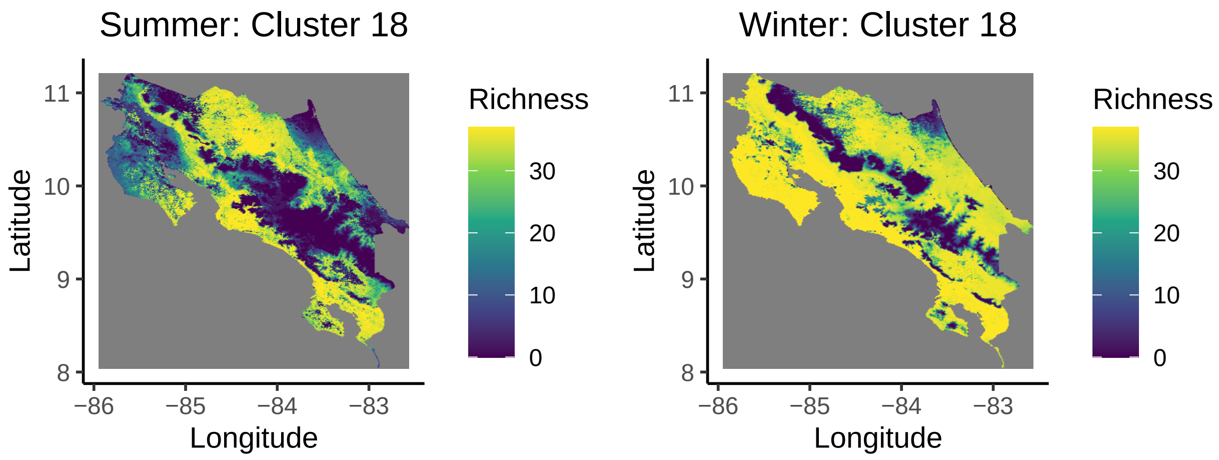

Differences in range between seasons

Note that the continuous rasters exist for Costa Rica and Panama; they must all be cropped to the area of Costa Rica for proper comparisons.

```
# note - same for both PDFs, since the training area is applied after

d.stat=NULL

y=readOGR(paste0(shp.path,"Acanthidops-bairdi.gpkg"))

# get Schoener's D values
# use continuous rasters
for(i in 1:length(name.list)){
  file=name.list[i]

  sub.files=paste0(file,"_all-pts.tif")

  r1=raster(paste0(filepath,"SanJose_june/output/all/",sub.files))
  cr1=crop(r1,y)
  cr2=raster::mask(cr1,y)

  r2=raster(paste0(filepath,"MadreSelva_december/output_all/all/",sub.files))
  c2r1=crop(r2,y)
  c2r2=raster::mask(c2r1,y)
```

```
  d.stat[i]=nicheOverlap(r1,r2,stat="D")
}

new.df=cbind(x,d.stat)

write_csv(new.df,paste0(filepath,"clust_d-stats_null.csv"))
```

```
x=read_csv(paste0(filepath,"clust_d-stats.csv"))
```

```
## Rows: 460 Columns: 4
## -- Column specification --------------------------------------------------------
## Delimiter: ","
## chr (1): Name
## dbl (3): Cluster.x, Cluster.y, d.stat
##
## i Use `spec()` to retrieve the full column specification for this data.
## i Specify the column types or set `show_col_types = FALSE` to quiet this message.
```

Now we can look at this differences a little more closely.

```
brks=seq(0,1,0.05)
hist(x$d.stat,breaks=brks)
```

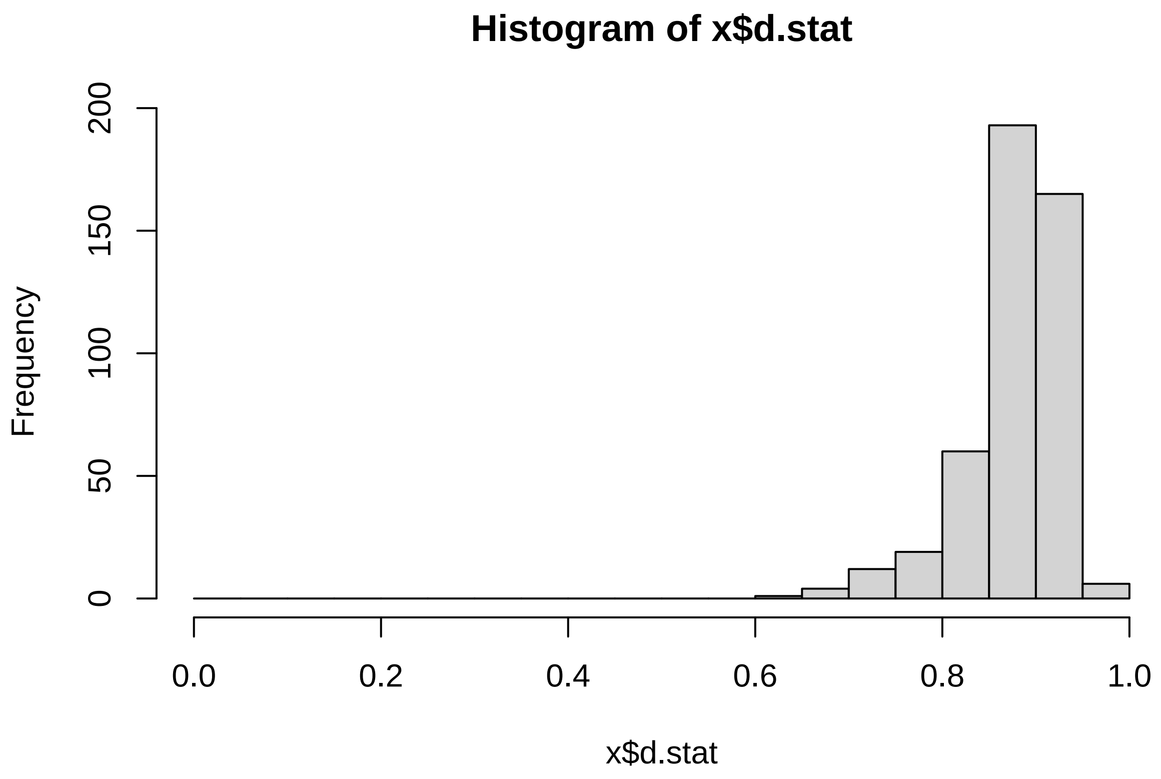

Most species are fairly similar in their D statistic, and we don't see any bimodality occurring. Now we can look at differences between groups.

```
sub.x=x[x$Cluster.x==stable,]
```

```
## Warning in x$Cluster.x == stable: longer object length is not a multiple of
## shorter object length
```

```
hist(sub.x$d.stat,breaks=brks,main="Stable")
```

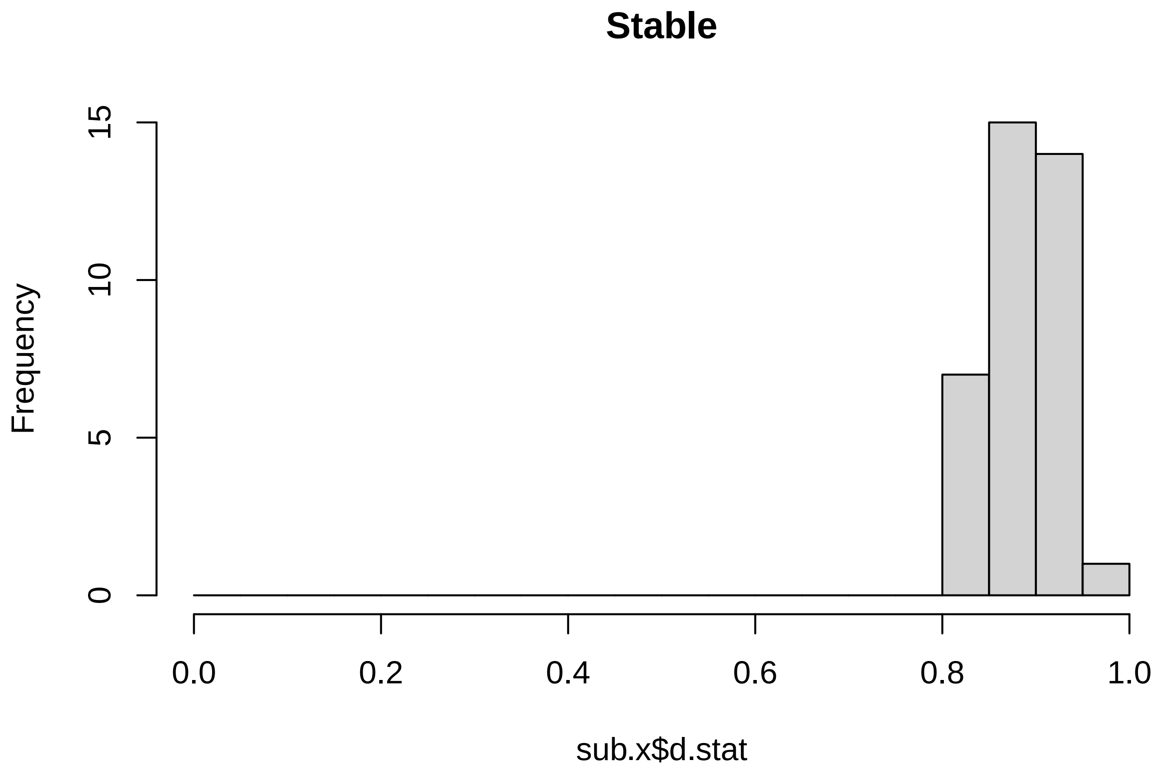


```
sub.x=x[x$Cluster.x==dispersed,]
```

```
## Warning in x$Cluster.x == dispersed: longer object length is not a multiple of
## shorter object length
```

```
hist(sub.x$d.stat,breaks=brks,main="Diffuse")
```

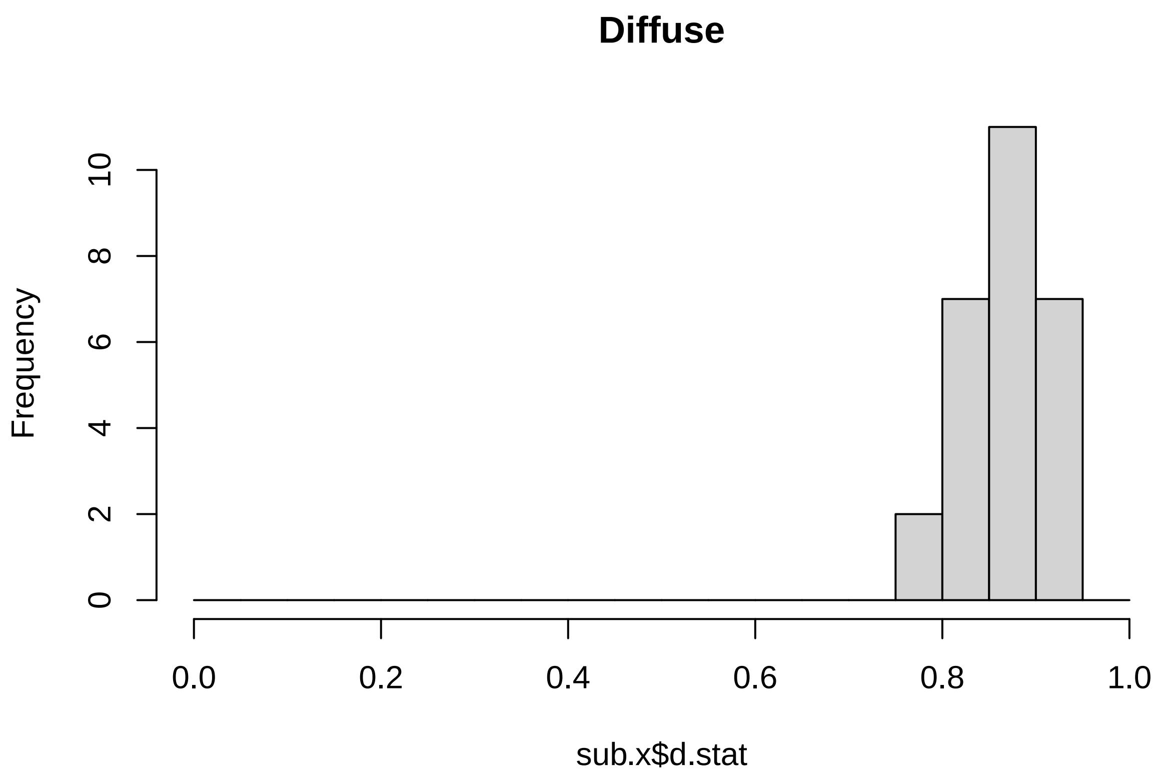


```
sub.x=x[x$Cluster.x==split,]
```

```
## Warning in x$Cluster.x == split: longer object length is not a multiple of
## shorter object length
```

```
hist(sub.x$d.stat,breaks=brks,main="Split")
```

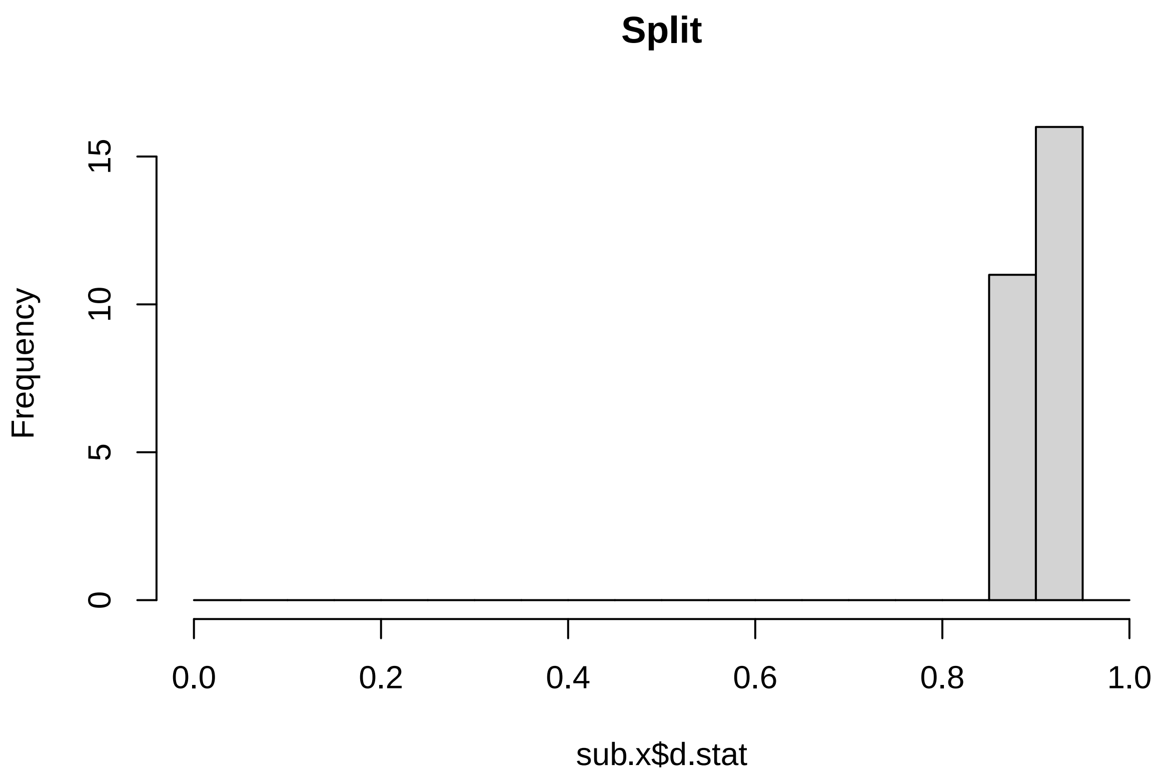

Interestingly, no groups show major trends towards niche shifting. This may imply species are using different parts of their niches at different times of the year.

```
stable.x=x[x$Cluster.x==stable,]
```

```
## Warning in x$Cluster.x == stable: longer object length is not a multiple of
## shorter object length
```

```
diffuse.x=x[x$Cluster.x==dispersed,]
```

```
## Warning in x$Cluster.x == dispersed: longer object length is not a multiple of
## shorter object length
```

```
split.x=x[x$Cluster.x==split,]
```

```
## Warning in x$Cluster.x == split: longer object length is not a multiple of
## shorter object length
```

```
# Stable vs. Diffuse
wilcox.test(stable.x$d.stat,diffuse.x$d.stat)
```

```
##
##  Wilcoxon rank sum exact test
##
## data:  stable.x$d.stat and diffuse.x$d.stat
## W = 653, p-value = 0.0369
## alternative hypothesis: true location shift is not equal to 0
```

```
# Split vs. Diffuse
wilcox.test(split.x$d.stat,diffuse.x$d.stat)
```

```
##
##  Wilcoxon rank sum exact test
##
## data:  split.x$d.stat and diffuse.x$d.stat
## W = 582, p-value = 9.933e-05
## alternative hypothesis: true location shift is not equal to 0
```

```
# Stable vs. Split
wilcox.test(stable.x$d.stat,split.x$d.stat)
```

```
##
##  Wilcoxon rank sum exact test
##
## data:  stable.x$d.stat and split.x$d.stat
## W = 354, p-value = 0.0482
## alternative hypothesis: true location shift is not equal to 0
```

```
## [1] "Stable: 0.888+/-0.014"
```

```
## [1] "Diffuse: 0.865+/-0.017"
```

```
## [1] "Split: 0.908+/-0.017"
```

```
x=x[order(x$d.stat),]

x[which(x$d.stat<0.8),]
```

```
## # A tibble: 36 x 4
##    Name                          Cluster.x Cluster.y d.stat
##    <chr>                             <dbl>     <dbl>  <dbl>
##  1 Celeus-castaneus                      1         1  0.645
##  2 Myiobius-atricaudus                   6         7  0.657
##  3 Phyllomyias-burmeisteri               5         3  0.664
##  4 Rhodinocichla-rosea                   4         6  0.683
##  5 Phaeomyias-murina                     4         7  0.695
##  6 Asio-clamator                         5         3  0.700
##  7 Cyanolyca-cucullata                   4         6  0.706
##  8 Anabacerthia-variegaticeps            4         6  0.713
##  9 Arremon-costaricensis                 6         7  0.718
## 10 Microchera-albocoronata              11         3  0.720
## # i 26 more rows
```

```
x[which(x$d.stat>0.935),]
```

```
## # A tibble: 37 x 4
##    Name                        Cluster.x Cluster.y d.stat
##    <chr>                           <dbl>     <dbl>  <dbl>
##  1 Melanerpes-rubricapillus            6         7  0.935
##  2 Pitangus-sulphuratus               15         2  0.936
##  3 Pheugopedius-atrogularis           12        10  0.936
##  4 Cantorchilus-thoracicus            12        10  0.936
##  5 Zonotrichia-capensis                5         8  0.937
##  6 Panterpe-insignis                  14         6  0.937
##  7 Molothrus-aeneus                   15         3  0.937
##  8 Catharus-fuscater                  16         8  0.937
##  9 Catharus-frantzii                  14         6  0.938
## 10 Patagioenas-flavirostris           15         3  0.938
## # i 27 more rows
```

Randomized D Stats

```r
# note - this is also the same as previous, since it is just for post-hoc
d.stat=NULL

y=readOGR(paste0(shp.path,"Acanthidops-bairdi.gpkg"))

# get Schoener's D values
# use continuous rasters

n=length(name.list)

for(i in 1:(n*10)){
  file1=name.list[round(runif(1,1,n),0)]
  file2=name.list[round(runif(1,1,n),0)]

  sub.files1=paste0(file1,"_all-pts.tif")
  sub.files2=paste0(file2,"_all-pts.tif")

  r1=raster(paste0(filepath,"SanJose_june/output/all/",sub.files1))
  cr1=crop(r1,y)
  cr2=raster::mask(cr1,y)

  r2=raster(paste0(filepath,"MadreSelva_december/output_all/all/",sub.files2))
  c2r1=crop(r2,y)
  c2r2=raster::mask(c2r1,y)

  d.stat[i]=nicheOverlap(r1,r2,stat="D")
}

new.df=cbind('x',d.stat)

write_csv(as.data.frame(new.df),paste0(filepath,"clust_d-stats_random.csv"))
```

```r
xx=read_csv(paste0(filepath,"clust_d-stats_random.csv"))
```

```
## Rows: 4600 Columns: 2
## -- Column specification --------------------------------------------------------
## Delimiter: ","
## chr (1): V1
## dbl (1): d.stat
##
## i Use `spec()` to retrieve the full column specification for this data.
## i Specify the column types or set `show_col_types = FALSE` to quiet this message.
```

```r
hist(xx$d.stat)
```

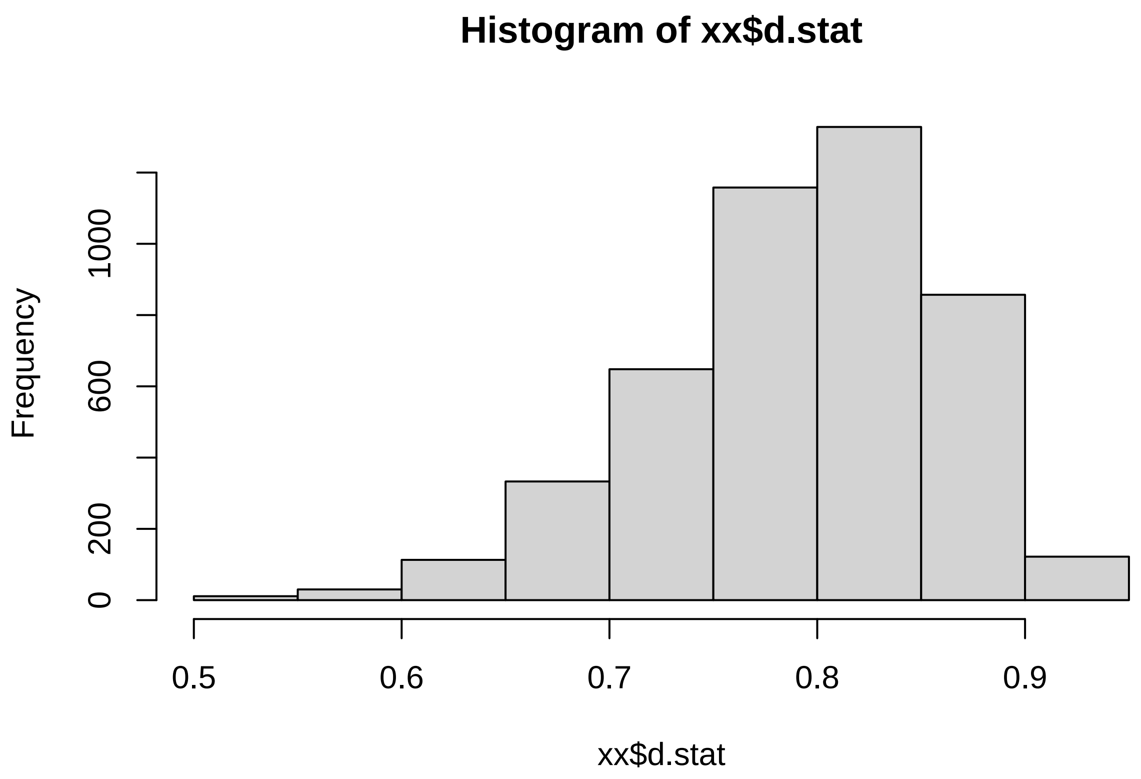


```
# randomized
summary(xx$d.stat)
```

```
##    Min. 1st Qu.  Median    Mean 3rd Qu.    Max.
##  0.5090  0.7510  0.8002  0.7924  0.8433  0.9471
```

```
# nonrandom
summary(x$d.stat)
```

```
##    Min. 1st Qu.  Median    Mean 3rd Qu.    Max.
##  0.6449  0.8556  0.8878  0.8773  0.9140  0.9745
```

```
t.test(xx$d.stat,x$d.stat)
```

```
##
##  Welch Two Sample t-test
##
## data:  xx$d.stat and x$d.stat
## t = -32.06, df = 627.11, p-value < 2.2e-16
## alternative hypothesis: true difference in means is not equal to 0
## 95 percent confidence interval:
##  -0.09014435 -0.07973858
## sample estimates:
## mean of x mean of y
## 0.7923693 0.8773108
```

The D statistics of the actual observed species are significantly higher than expected at random.

Number of points

Calculate the difference in points between summer and winter.

```
sum.pts=NULL
win.pts=NULL
dif.pts=NULL
name.vector=NULL

s.point.files=summer.point.files
w.point.files=winter.point.files

for(i in 1:length(name.list)){
  name=name.list[i]
  x=read_csv(s.point.files[which(s.point.files%like%name)])
  y=read_csv(w.point.files[which(w.point.files%like%name)])

  name.vector[i]=name
  sum.pts[i]=nrow(x)
  win.pts[i]=nrow(y)
}

dif.pts=sum.pts-win.pts

pts.frame=as.data.frame(cbind(name.vector,sum.pts,win.pts,dif.pts))
```

```
## Rows: 460 Columns: 4
## -- Column specification --------------------------------------------------------
## Delimiter: ","
## chr (1): Name
## dbl (3): sum.pts, win.pts, dif.pts
##
## i Use `spec()` to retrieve the full column specification for this data.
## i Specify the column types or set `show_col_types = FALSE` to quiet this message.
## Rows: 460 Columns: 4
## -- Column specification --------------------------------------------------------
## Delimiter: ","
## chr (1): Name
## dbl (3): Cluster.x, Cluster.y, d.stat
##
## i Use `spec()` to retrieve the full column specification for this data.
## i Specify the column types or set `show_col_types = FALSE` to quiet this message.
```

```
pts.d=inner_join(pts.frame,d.stats,by="Name")

plot(x=pts.d$d.stat,y=pts.d$dif.pts,pch=19)
```

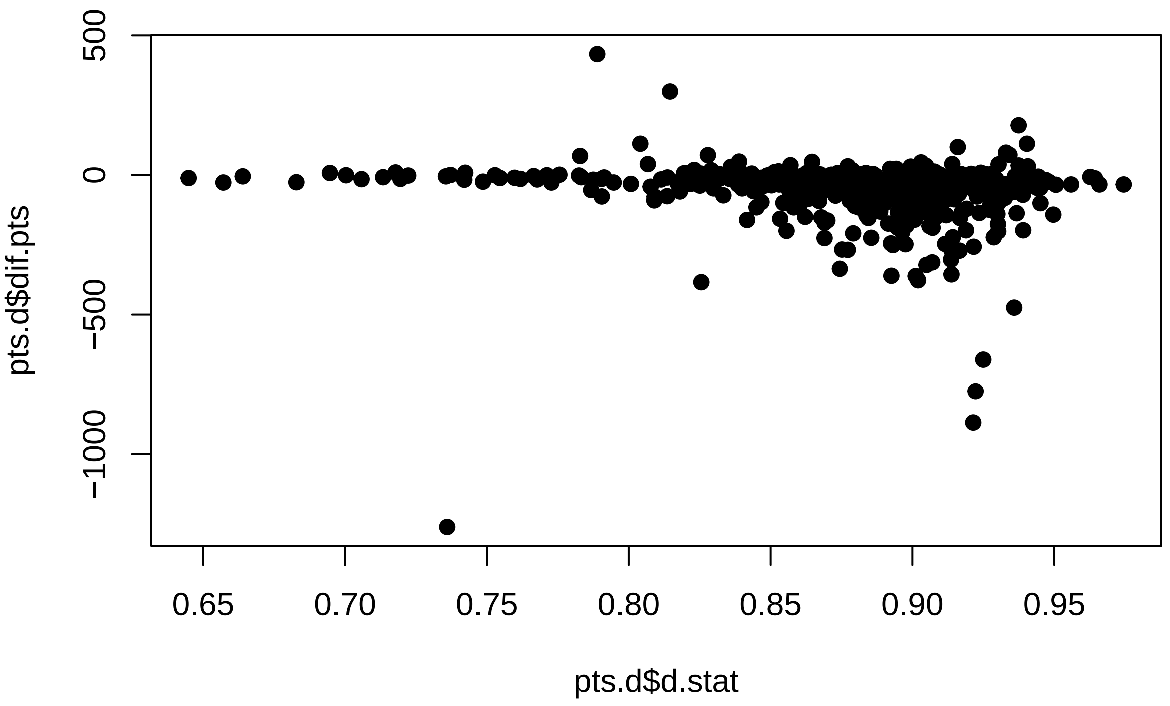
500
0
−500
−1000
pts.d$dif.pts
0.65
0.70
0.75
0.80
0.85
0.90
0.95
pts.d$d.stat
