# Supplementary figures and images for: What constitutes a community? A co-occurrence exploration of the Costa Rican avifauna
Source: Neotrop Biodivers. Author manuscript; Available in PMC 2023 Jun 2. (PMC10237366; doi:10.1080/23766808.2023.2204549)

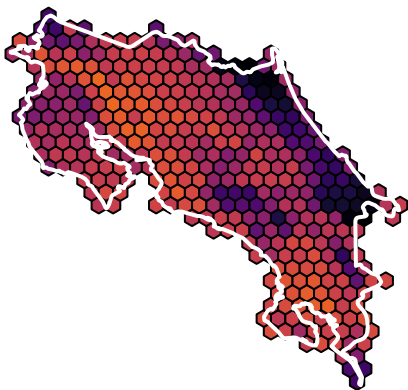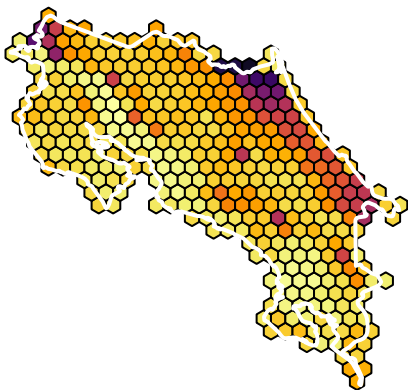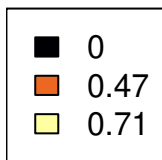

Supplement: Supplementary Material [file NIHMS1893357-supplement-Supplementary_Material.zip › beta_diversity-eps-converted-to.pdf]

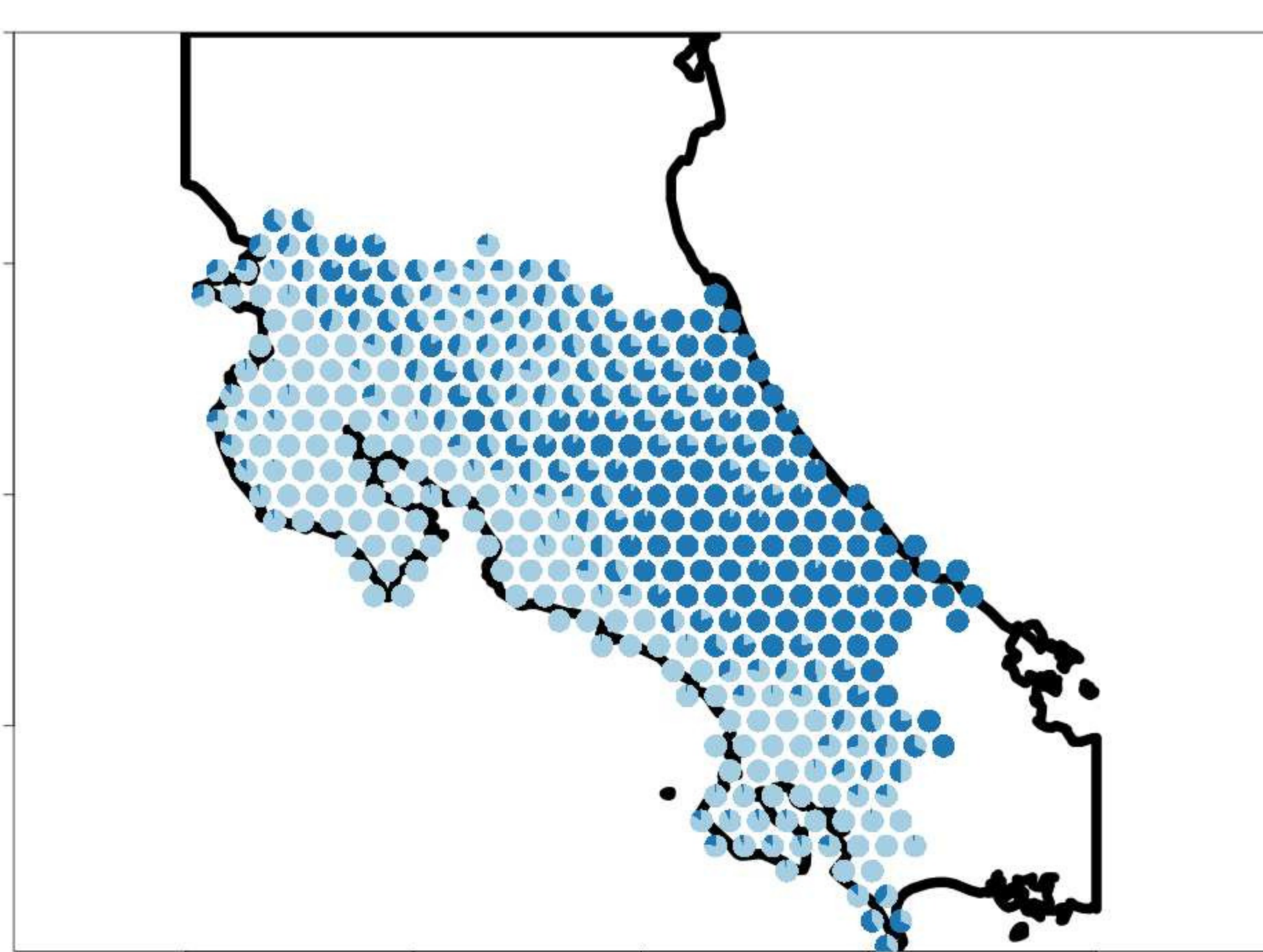

86°W 85°W 84°W 83°W 82°W

12°N

11°N

10°N

9°N

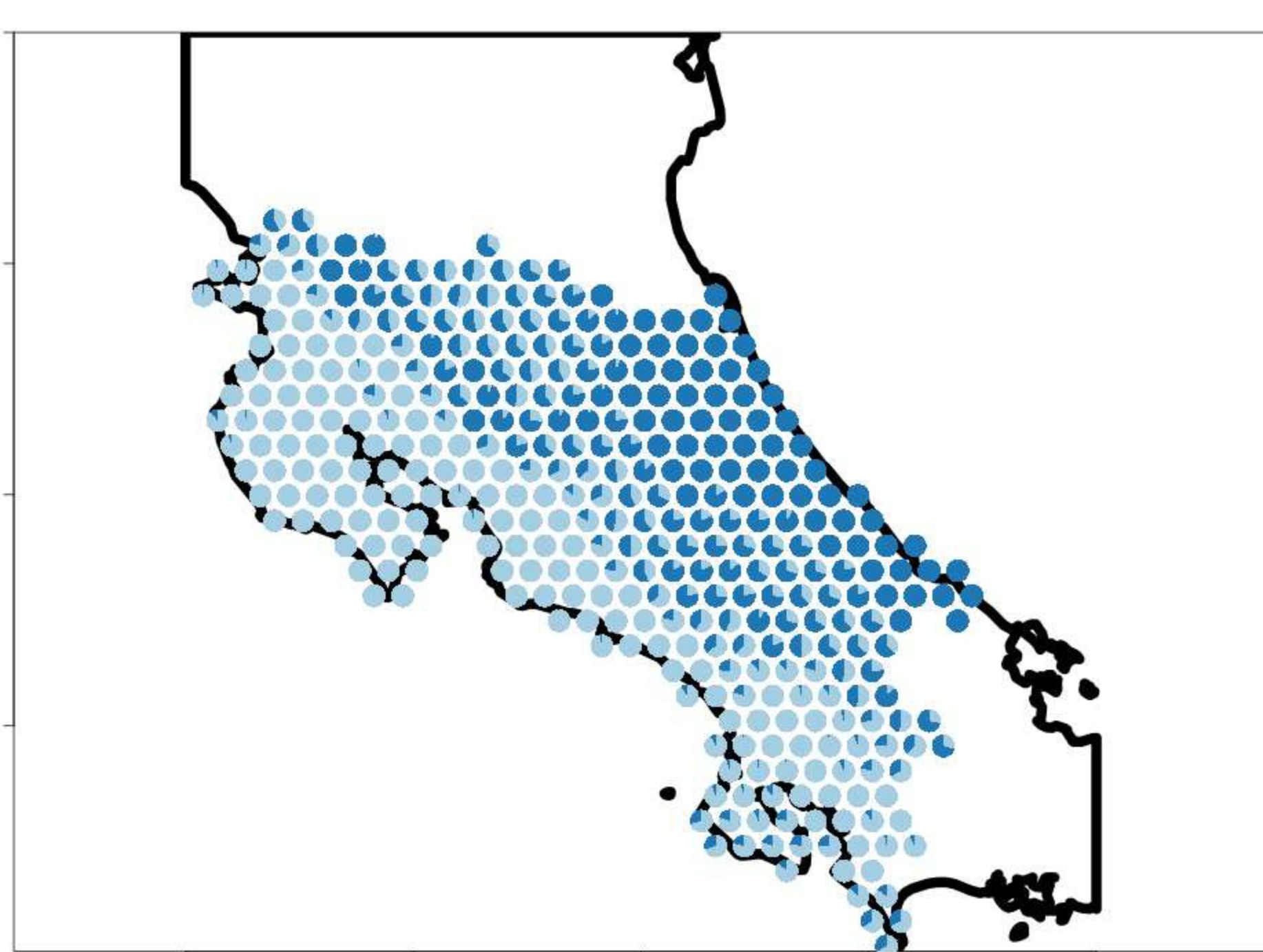

86°W 85°W 84°W 83°W 82°W

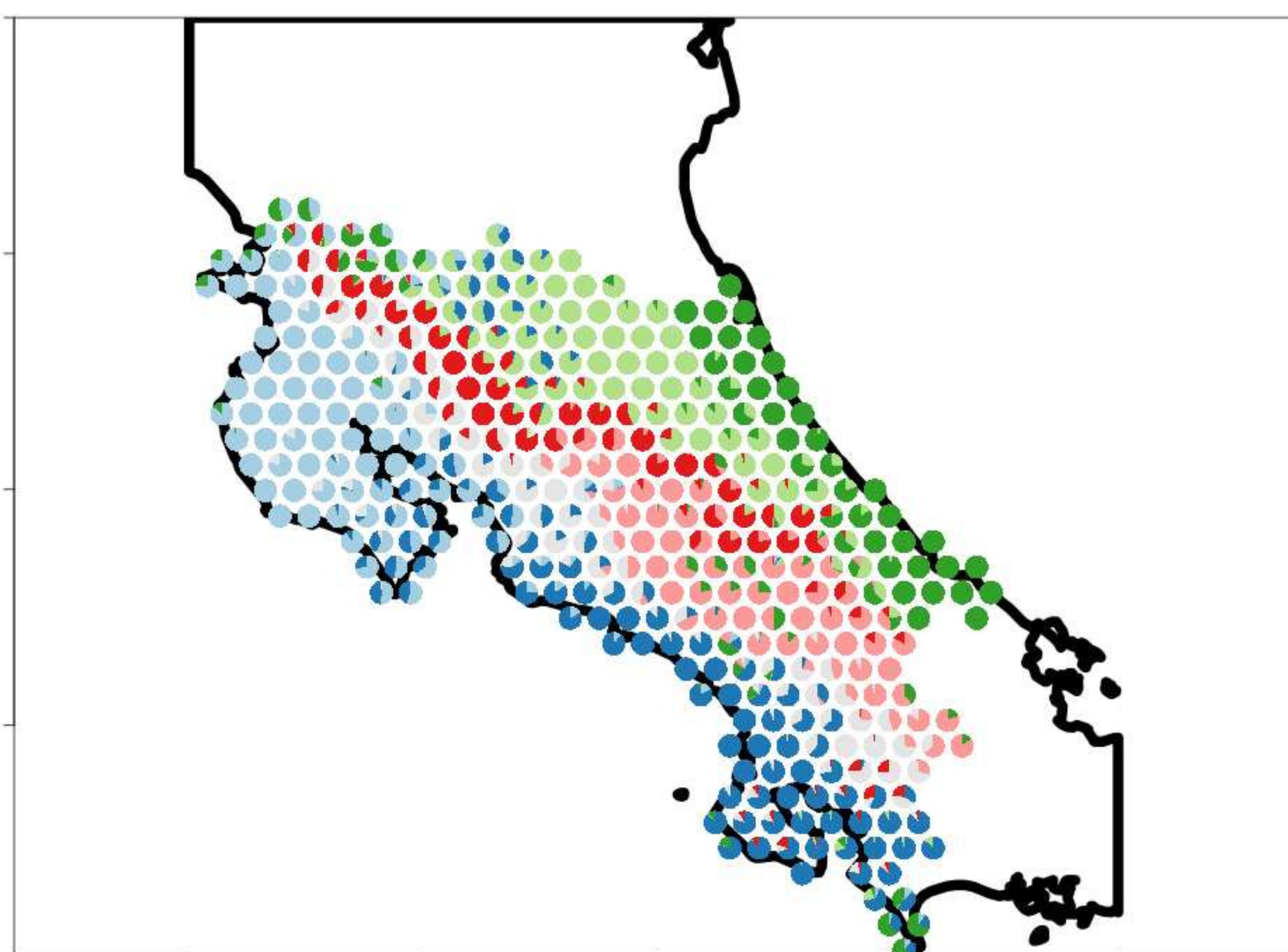

86°W 85°W 84°W 83°W 82°W

12°N

11°N

10°N

9°N

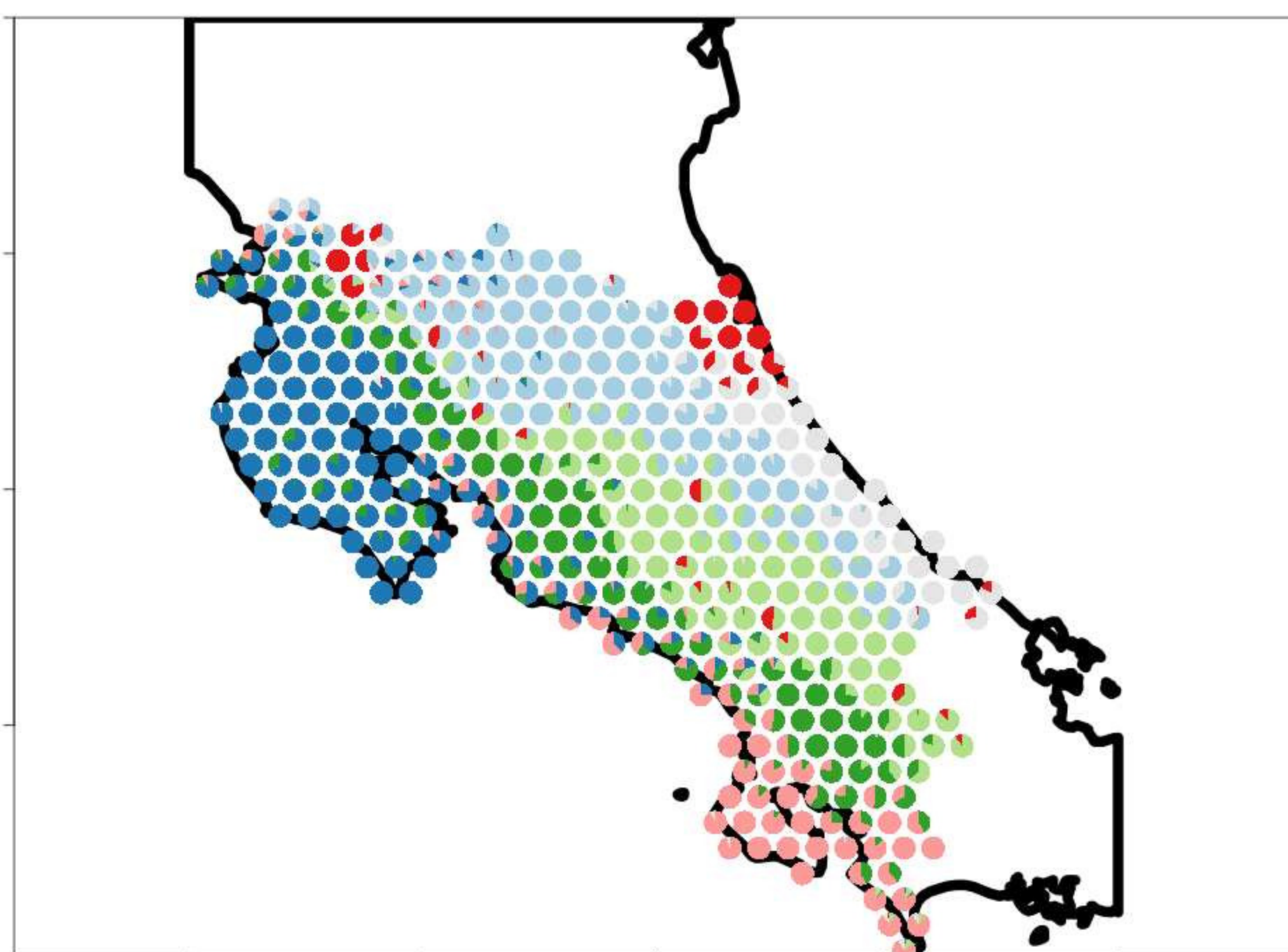

86°W 85°W 84°W 83°W 82°W

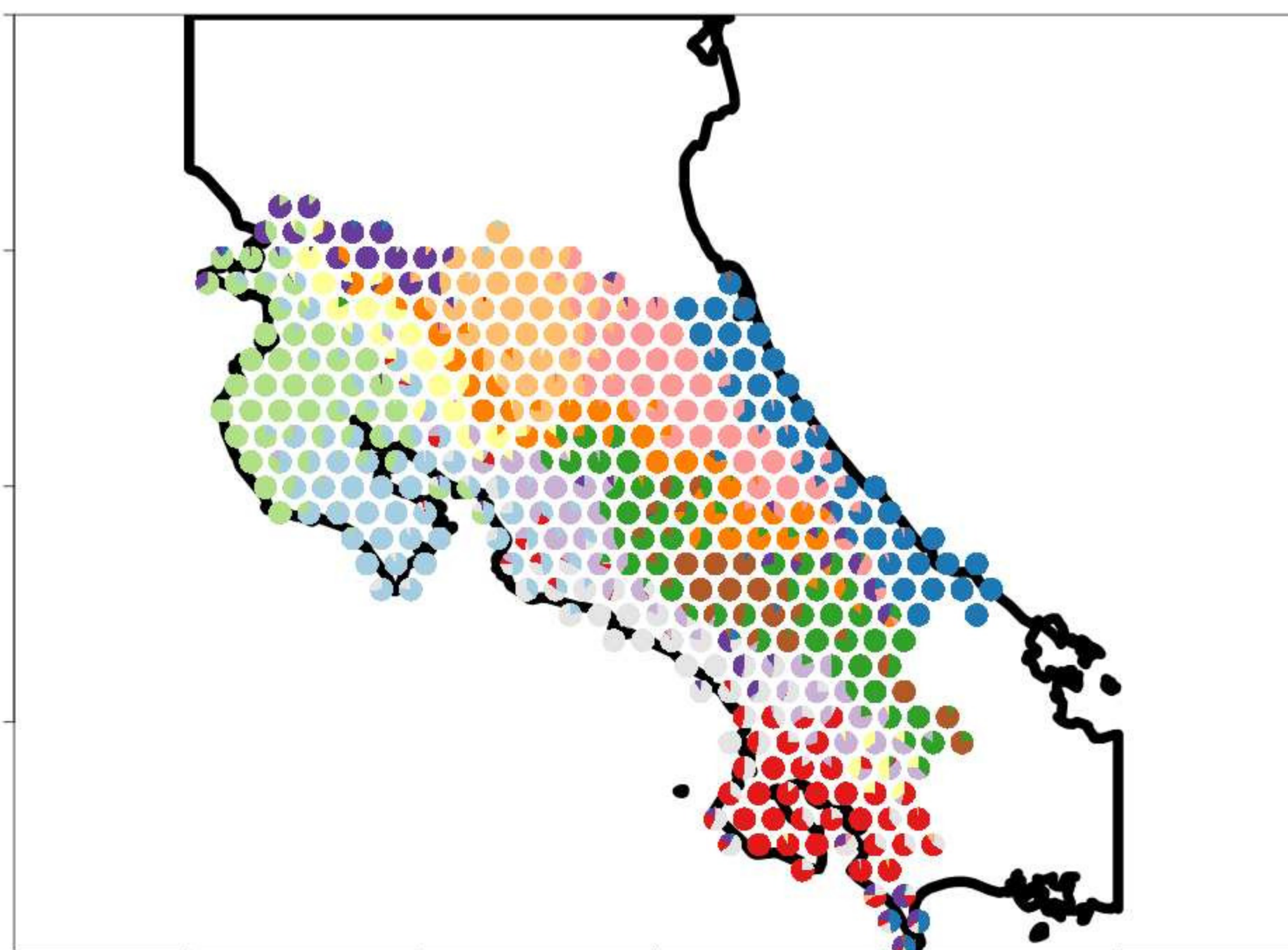

86°W 85°W 84°W 83°W 82°W

12°N

11°N

10°N

9°N

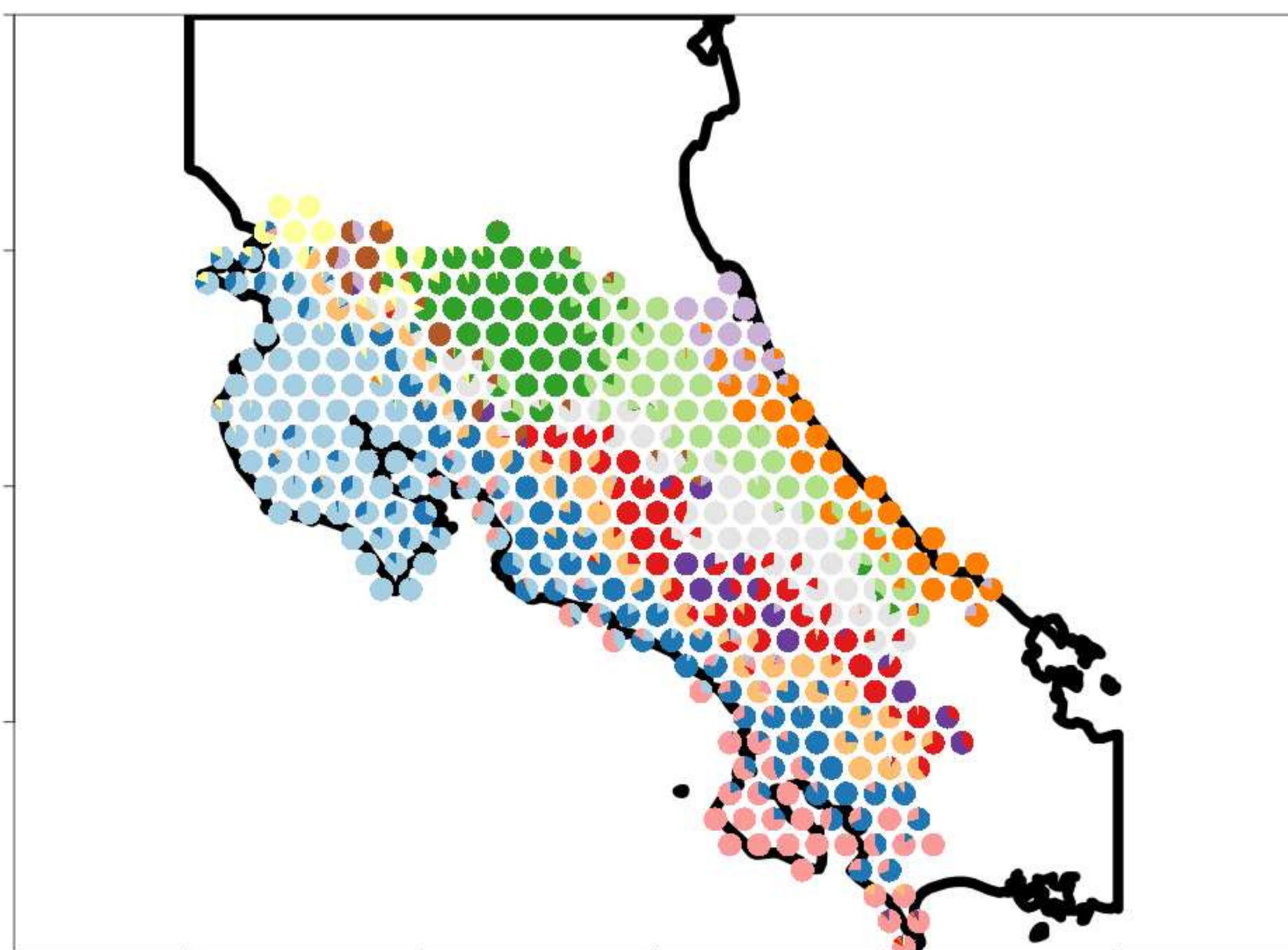

86°W 85°W 84°W 83°W 82°W

Supplement: Supplementary Material [file NIHMS1893357-supplement-Supplementary_Material.zip › eco_figure-eps-converted-to.pdf]

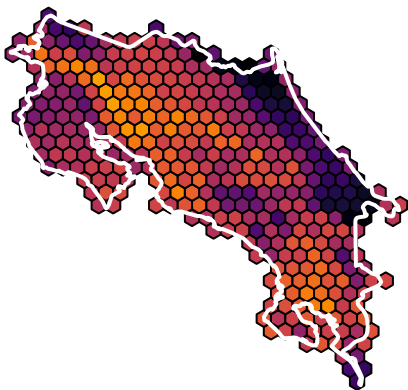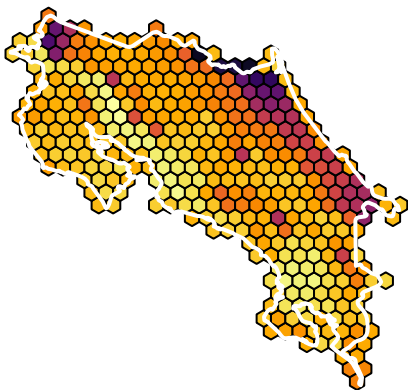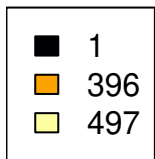

Supplement: Supplementary Material [file NIHMS1893357-supplement-Supplementary_Material.zip › richness-eps-converted-to.pdf]
